# SnRK1α1-mediated RBOH1 phosphorylation regulates reactive oxygen species to enhance tolerance to low nitrogen in tomato

**DOI:** 10.1093/plcell/koae321

**Published:** 2024-12-12

**Authors:** Xuelian Zheng, Hongfei Yang, Jinping Zou, Weiduo Jin, Zhenyu Qi, Ping Yang, Jingquan Yu, Jie Zhou

**Affiliations:** Department of Horticulture, Zhejiang Key Laboratory of Horticultural Crop Quality Improvement, Zijingang Campus, Zhejiang University, Hangzhou 310058, China; Key Laboratory of Horticultural Plants Growth, Development and Quality Improvement, Ministry of Agriculture and Rural Affairs of China, Hangzhou 310058, China; Department of Horticulture, Zhejiang Key Laboratory of Horticultural Crop Quality Improvement, Zijingang Campus, Zhejiang University, Hangzhou 310058, China; Key Laboratory of Horticultural Plants Growth, Development and Quality Improvement, Ministry of Agriculture and Rural Affairs of China, Hangzhou 310058, China; Department of Horticulture, Zhejiang Key Laboratory of Horticultural Crop Quality Improvement, Zijingang Campus, Zhejiang University, Hangzhou 310058, China; Key Laboratory of Horticultural Plants Growth, Development and Quality Improvement, Ministry of Agriculture and Rural Affairs of China, Hangzhou 310058, China; Department of Horticulture, Zhejiang Key Laboratory of Horticultural Crop Quality Improvement, Zijingang Campus, Zhejiang University, Hangzhou 310058, China; Key Laboratory of Horticultural Plants Growth, Development and Quality Improvement, Ministry of Agriculture and Rural Affairs of China, Hangzhou 310058, China; Agricultural Experiment Station, Zhejiang University, Hangzhou 310058, China; Agricultural Experiment Station, Zhejiang University, Hangzhou 310058, China; Department of Horticulture, Zhejiang Key Laboratory of Horticultural Crop Quality Improvement, Zijingang Campus, Zhejiang University, Hangzhou 310058, China; Key Laboratory of Horticultural Plants Growth, Development and Quality Improvement, Ministry of Agriculture and Rural Affairs of China, Hangzhou 310058, China; Department of Horticulture, Zhejiang Key Laboratory of Horticultural Crop Quality Improvement, Zijingang Campus, Zhejiang University, Hangzhou 310058, China; Key Laboratory of Horticultural Plants Growth, Development and Quality Improvement, Ministry of Agriculture and Rural Affairs of China, Hangzhou 310058, China

## Abstract

Nitrogen is essential for plant growth and development. SNF1-related protein kinase 1 (SnRK1) is an evolutionarily conserved protein kinase pivotal for regulating plant responses to nutrient deficiency. Here, we discovered that the expression and activity of the SnRK1 α-catalytic subunit (SnRK1α1) increased in response to low-nitrogen stress. *SnRK1α1* overexpression enhanced seedling tolerance, nitrate uptake capacity, apoplastic reactive oxygen species (ROS) accumulation, and NADPH oxidase activity in tomato (*Solanum lycopersicum* L.) under low-nitrogen stress compared to wild type plants, while *snrk1α1* mutants exhibited the opposite phenotypes. Mutation of the NADPH oxidase gene *Respiratory burst oxidase homolog 1* (*RBOH1*) suppressed numerous nitrate uptake and metabolism genes during low-nitrogen stress. *rboh1* mutants displayed lower NADPH oxidase activity, apoplastic ROS production, and seedling tolerance to low nitrogen. Silencing *RBOH1* expression also compromised SnRK1α1-mediated seedling tolerance to low-nitrogen stress. SnRK1α1 interacts with and activates RBOH1 through phosphorylation of three N-terminal serine residues, leading to increased apoplastic ROS production and enhanced tolerance to low nitrogen conditions. Furthermore, RBOH1-dependent ROS oxidatively modified the transcription factor TGA4 at residue Cys-334, which increased *NRT1.1* and *NRT2.1* expression under low-nitrogen stress. These findings reveal a SnRK1α1-mediated signaling pathway and highlight the essential role of RBOH1-dependent ROS production in enhancing plant tolerance to low nitrogen.

## Teaser

A kinase and an NADPH oxidase mediate reactive oxygen species production, which enhances the expression of two nitrate transporter genes and promotes nitrate uptake in tomato.

## Introduction

Nitrogen is a crucial macronutrient for plant growth, health, and yield. Despite its limited availability in natural soils, plants have evolved sophisticated mechanisms to optimize nitrogen utilization, particularly under low nitrogen conditions (Kiba and Krapp 2016). This adaptation involves enhancing nitrogen uptake and usage efficiency to ensure effective nitrogen management for plant growth (Hermans et al. 2006; Nacry et al. 2013). Nitrogen is taken up through specific plasma membrane-localized transporters in the root in the form of ammonium and nitrate. Ammonium transport is mediated by the AMT/MEP/Rh superfamily (Ludewlg et al. 2007), while nitrate uptake is managed by two transporter families: nitrate transporter 1 (NRT1) and NRT2. NRT1.1 (also known as NRT1/PTR FAMILY 6.3 [NPF6.3] and CHLORINA 1 [CHL1]) possesses dual-affinity transport and nitrate sensing capabilities and plays a crucial role in nitrate uptake (Ho et al. 2009), while NRT2-type transporters, particularly NRT2.1, are high-affinity nitrate transporters essential for efficient uptake under low nitrogen conditions (Kotur et al. 2012; Lezhneva et al. 2014). Transcription factors such as TGACG MOTIF-BINDING FACTOR 1 (TGA1) and TGA4, along with TEOSINTE BRANCHED 1/CYCLOIDEA/PROLIFERATING CELL FACTOR 20 (TCP20), enhance the expression of *NRT1.1* and *NRT2.1* by binding to their promoters and promoting lateral root development under low nitrate conditions (Alvarez et al. 2014; Guan et al. 2014). Soil pH also affects nitrate transporter gene expression; the transcription factor SENSITIVE TO PROTON RHIZOTOXICITY1 (STOP1) can activate *NRT1.1* transcription under acidic conditions, thereby enhancing nitrate uptake under specific environmental conditions (Ye et al. 2021).

During nutritional stress, plants adjust their energy metabolism through the utilization of target of rapamycin (TOR) and sucrose-nonfermenting 1-related kinase 1 (SnRK1) signaling pathways (Rodriguez et al. 2019). In plants, SnRK1 and TOR generally exhibit antagonistic behaviors (Belda-Palazón et al. 2022) by sensing and reacting to nutritional abundance (Fu et al. 2020; Liu et al. 2021b). In the root apical meristem, glucose activates TOR, initiating a cascade that upregulates the transcription of genes involved in cell cycle progression and DNA synthesis, activating the root meristem (Xiong et al. 2013). SnRK1, functionally like the yeast (*Saccharomyces cerevisiae*) sucrose nonfermenting 1 (SNF1) and mammalian AMP-activated protein kinase (AMPK), acts as a central hub for energy and metabolism, coordinating plant acclimation to stress (Chen et al. 2017; Wang et al. 2020b; Muralidhara et al. 2021). In plants, SnRK1 forms a heterotrimeric complex consisting of catalytic α-subunits, substrate-interacting β-subunits, and regulatory γ-subunits (Emanuelle et al. 2015; Wang et al. 2020b). As a kinase, SnRK1 phosphorylates and activates proteins involved in carbon, nitrogen, and lipid metabolisms, such as sucrose phosphate synthase and trehalose-6-phosphate synthase (Tre6P) (Wang et al. 2020b). During carbon starvation in the root, SnRK1 also phosphorylates BASIC LEUCINE ZIPPER63 (bZIP63), which directly binds to the promoter of and activates the expression of the key regulatory factor gene, *AUXIN-RESPONSE FACTOR 19* (*ARF19*), during lateral roots initiation (Muralidhara et al. 2021). Additionally, knockout of *SnRK1β1* suppresses the expression of *NRT1.8*, which primarily affects long-distance nitrate transport from root to shoot in Arabidopsis (*Arabidopsis thaliana*) (Wang et al. 2020b). SnRK1α1 orchestrates the balance between carbon and nitrogen in plants by phosphorylating NIN-LIKE PROTEIN 7 (NLP7), the principal regulator of the nitrate signaling pathway, and facilitating its translocation to the cytoplasm for subsequent degradation (Wang et al. 2022). In Arabidopsis, SnRK1α1 enhances plant tolerance to nutritional stress by interacting with the AUTOPHAGY 1 (ATG1)–ATG13 protein complex, which can activate autophagy in nitrogen and carbon starvation (Chen et al. 2017). Overall, the multifaceted functions of SnRK1 underscore its importance in plant acclimation to varying environmental conditions.

Reactive oxygen species (ROS) are crucial to plant stress responses (Gilroy et al. 2014). They can be produced in various organelles, such as the chloroplasts, mitochondria, peroxisomes, and by the plasma membrane redox enzyme system (Mittler et al. 2022). In plants, NADPH oxidase/respiratory burst oxidase homolog (RBOH) proteins generate localized bursts of ROS, which are essential for regulating growth, development, and stress responses (Mittler et al. 2022). In Arabidopsis, the RBOH family consists of 10 members (RBOHA to RBOHJ), each with specific roles in development and stress response (Sagi and Fluhr 2006). Arabidopsis *RBOHD* and *RBOHF*, which are highly expressed in root tissues, play key roles in modulating root growth and responses to environmental stimuli (Li et al. 2015; Chapman et al. 2019). Arabidopsis RBOHF is significant in abiotic stress responses, such as abscisic acid (ABA) signaling and salinity stress tolerance (Kwak et al. 2003; Ma et al. 2012; Jiang et al. 2013). In tomato (*Solanum lycopersicum* L.), the RBOH family comprises eight members (RBOH1, RBOHA–F, and RBOHH), with RBOH1 (Solyc08g081690) most similar to Arabidopsis RBOHF. Tomato *RBOH1* is highly expressed in the root (Yan et al. 2020) and is involved in heat and oxidative stress response through H_2_O_2_ signaling (Zhou et al. 2014a, 2014b). However, the role of RBOHs in nutrient stress tolerance and their regulatory mechanisms in plants remain largely unexplored. RBOH activation is primarily achieved through N-terminal phosphorylation (Wang et al. 2020a). In Arabidopsis, BOTRYTIS-INDUCED KINASE 1 (BIK1) interacts with RBOHD and directly phosphorylates its N-terminal domain at the serine (S) residues S39, S343, and S347, activating RBOHD for ROS signaling in response to microbial pathogens (Kadota et al. 2014; Li et al. 2014). CBL-INTERACTING PROTEIN KINASE 26 (CIPK26) interacts with and phosphorylates the N terminus of RBOHF, negatively modulating its ROS-producing activity by inducing Ca^2+^ influx into HEK293T cells (Kimura et al. 2012). The ABA-activated Arabidopsis SnRK2 protein kinase OPEN STOMATA 1 (OST1, also reported as SRK2E and SnRK2.6) phosphorylates the N terminus of RBOHF at residues S13 and S174 to positively regulate ABA signaling (Sirichandra et al. 2009). Thus, NADPH oxidases are subject to complex regulation under diverse environmental stress stimuli, triggering activation by various kinases at multiple amino acids. However, whether ROS production under nutrient deficiency is related to the phosphorylated state of RBOHs has not yet been investigated.

ROS accumulation during stress affects the redox state of various proteins, including enzymes, receptors, and small molecules. This accumulation can activate, modify, or integrate multiple stress-response signal transduction pathways, thereby altering gene expression and enhancing plant resilience to stress (Zou et al. 2015; Nietzel et al. 2020; Sies and Jones 2020; Liu et al. 2021a). Hydrogen peroxide (H_2_O_2_) is the most enduring ROS due to its comparatively long half-life within living cells (Bi et al. 2022). H_2_O_2_ can directly oxidize proteins through oxidative post-translational modifications (Oxi-PTMs), primarily targeting methionine and cysteine residues. These modifications can alter protein conformation, subcellular localization, and activity, including that of transcription factors, thus triggering H_2_O_2_ signaling pathways (Zhou et al. 2023; Zhang et al. 2024). For example, H_2_O_2_ facilitates the oxidation of inactive C-REPEAT BINDING TRANSCRIPTION FACTOR (CBF) oligomers and monomers, suppressing the expression of cold-responsive genes under cold stress (Lee et al. 2021). Additionally, H_2_O_2_-induced Oxi-PTM enhances BRASSINAZOLE-RESISTANT 1 (BZR1) transcriptional activity, regulating plant growth by promoting its interaction with key regulators in the auxin- and light-signaling pathways, including ARF6 and PHYTOCHROME INTERACTING FACTOR4 (PIF4) (Tian et al. 2018). However, it is currently unclear whether ROS-induced Oxi-PTMs affect proteins related to nitrogen uptake and metabolism under low nitrogen stress.

Previous studies have shown that nitrogen deficiency promotes ROS production in roots (Jung et al. 2018; Safi et al. 2021) and confirmed the crucial role of ROS in root response to nitrogen deprivation (Shin et al. 2005; Jung et al. 2018; Safi et al. 2021), but the upstream mechanism driving ROS production and the relationship between ROS and nitrogen uptake remain unclear. Additionally, while SnRK1α1, the most functionally active subunit of SnRK1, improves tolerance to low-nitrogen stress by activating autophagy in Arabidopsis (Chen et al. 2017), it is unknown whether SnRK1α1 regulates low-nitrogen stress tolerance through other pathways. In the present study, we found that SnRK1α1 responds to nitrate deficiency by activating RBOH1-dependent ROS production. ROS directly influence protein activation through H_2_O_2_-mediated Oxi-PTMs on cysteine residues. Specifically, the Oxi-PTM of the TGA4 protein at Cys-334 enhances its binding to the promoters of *NRT1.1* and *NRT2.1*, thereby enhancing their expression and promoting nitrate uptake. These findings provide insights into the regulatory mechanisms underlying low-nitrogen stress tolerance in plants.

## Results

### Role of SnRK1α1 in tomato low-nitrogen stress tolerance

**Figure 1.**
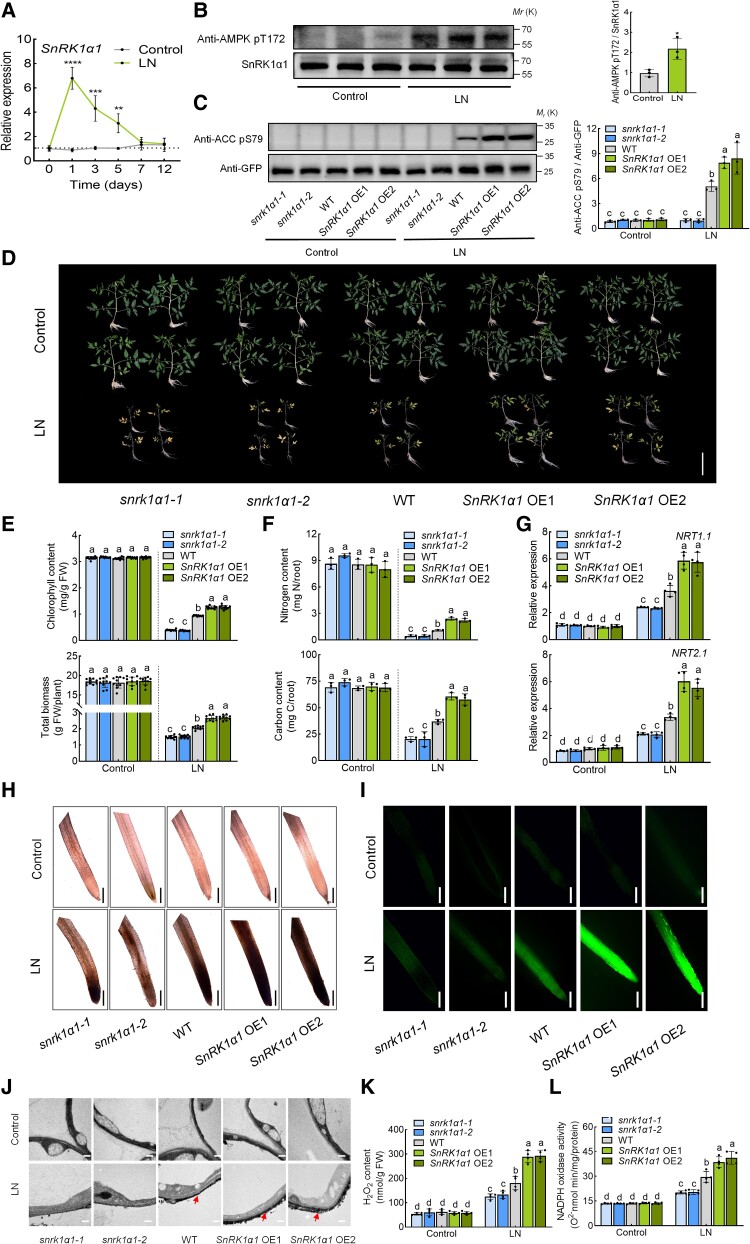
The role of SnRK1α1 in tomato response to low nitrogen stress. **A)** Expression of *SnRK1α1* under control and low nitrogen conditions. The relative expression of wild type (WT) under low nitrogen conditions at 0 d was set to “1”. **B)** In vivo activity of SnRK1α1 in WT under control and low nitrogen conditions. Root proteins were separated by SDS-PAGE, and the phosphorylation of SnRK1α1 was detected using immunoblotting with anti-AMPK pT172 antibody. SnRK1α1 protein was used as a loading control. Left, immunoblots showing the phosphorylation of SnRK1α1. Right, amounts of the phosphorylation of SnRK1α1 determined by densitometry of protein bands from three experiments. Band intensity was quantified by ImageJ. The ratio of anti-AMPK pT172/SnRK1α1 in the control was set to 1. **C)** In vivo activity of SnRK1α1 in WT, *snrk1α1* mutants and *SnRK1α1* overexpression (*SnRK1α1* OE) plants under control and low nitrogen conditions. Root proteins were separated by SDS-PAGE and the ACC reporter phosphorylation was detected using immunoblotting with anti-ACC pS79 antibody. GFP-ACC (peptide) was used as a loading control with anti-GFP antibody. Left, immunoblots showing the ACC reporter phosphorylation. Right, amounts of the ACC reporter phosphorylation determined by densitometry of protein bands from three experiments. Band intensity was quantified by ImageJ. The ratio of anti-ACC pS79/anti-GFP in the WT under control conditions was set to 1. **D)** Phenotypes of WT, *snrk1α1* mutants and *SnRK1α1* OE plants under low nitrogen stress. Bars: 10 cm. Images were digitally extracted for comparison. **E)** Chlorophyll content (upper panel) and biomass (lower panel) of WT, *snrk1α1* mutants, and *SnRK1α1* OE plants. **F)** Nitrogen (upper panel) and carbon (lower panel) contents in the roots of WT, *snrk1α1* mutants, and *SnRK1α1* OE plants under low nitrogen stress. Contents were calculated by multiplying the dry weight of the roots with their nitrogen and carbon concentrations. **G)** Gene expression of *NRT1.1* (upper panel) and *NRT2.1* (lower panel) in the roots of WT, *snrk1α1* mutants, and *SnRK1α1* OE plants under low nitrogen stress. Relative expression of WT under control conditions was set to “1”. **H)** In situ detection of ROS by 3,3′-diaminobenzidine (DAB) staining of roots. Bars for DAB staining: 300 *μ*m. Images were digitally extracted for comparison. **I)** H_2_DCF-DA fluorescence (DCF) staining of roots. Bars: 300 *μ*m. **J)** Detection of apoplastic H_2_O_2_ by CeCl_3_ staining under low nitrogen stress. Bars: 0.5 *μ*m. Arrows indicate the accumulation of H_2_O_2_. **K)** H_2_O_2_ content in the roots of WT, *snrk1α1* mutants, and *SnRK1α1* OE plants under low nitrogen stress. **L)** NADPH oxidase activity in the roots of WT, *snrk1α1* mutants, and *SnRK1α1* OE plants under low nitrogen stress. Error bars represent SD; data are means ± SD of 3 biological replicates in **(B, C, F)**, 4 biological replicates in **(A, G, K, L)**, and 10 biological replicates in **(D, E, H, I, J)**. Experiments were repeated three times with similar results. Asterisks above the bars represent significant differences by Student's *t*-tests in **(A, B)**. ****, *P* < 0.001; ***, *P* < 0.001; **, *P* < 0.01; *, *P* < 0.05. Distinct letters above the bars signify significant differences at *P* < 0.05 level, as determined by one-way ANOVA **(E, F)** and two-way ANOVA **(C, G, K, L)** with Tukey's multiple comparison test. Precise *P*-values from these statistical tests are detailed in the Supplementary Data Set 10.

To identify *SlSnRK1α1*, we analyzed the amino acid sequence from Arabidopsis and putative tomato *SnRK1α1* genes using phylogenetic analysis (Supplementary Fig. S1, Supplementary Files 1 and 2). We identified SnRK1α1 (encoded by Solyc02g067030) as the homolog of AtSnRK1α1 from Arabidopsis (Supplementary Fig. S1). To investigate the function of SnRK1α1 under low nitrogen conditions, we performed RT-qPCR to measure its expression. Low nitrogen treatment induced a 6.4-fold increase in *SnRK1α1* expression levels at 1 d into treatment, with a consistent and significant elevation from 3 to 5 d compared to the control condition (Fig. 1A). Consistent with its expression pattern, SnRK1α1 activity was significantly induced after 3 d of low nitrogen treatment (Fig. 1B).

To assess the function of SnRK1α1 under low nitrogen, we generated knockout mutants (*snrk1α1-1* and *snrk1α1-2*) and overexpressing lines (*SnRK1α1* OE1 and *SnRK1α1* OE2) (Supplementary Fig. S2, A to C). Interestingly, while *snrk1α1* mutants did not show significant growth differences compared to the wild type (WT) under normal conditions, they failed to set fruit normally after flowering (Supplementary Fig. S3). Thus, we obtained homozygous *snrk1α1* mutants by heterozygote isolation in this study. *snrk1α1* mutants exhibited almost complete loss of SnRK1α1 activity, while *SnRK1α1* OE lines showed significantly increased activity compared to WT under low nitrogen conditions (Fig. 1C). Under normal conditions, WT, *snrk1α1* mutants and *SnRK1α1* OE plants exhibited similar growth (Fig. 1D). However, after 3 weeks of low nitrogen treatment, *snrk1α1* mutants displayed severe leaf yellowing, while WT plants showed milder chlorosis. By contrast, *SnRK1α1* OE plants maintained predominantly green leaves (Fig. 1D). Chlorophyll content and total biomass were significantly lower in *snrk1α1* plants than in WT under low-nitrogen stress; In comparison, *SnRK1α1* OE plants had higher chlorophyll content and biomass compared to WT (Fig. 1E). These results indicate that SnRK1α1 is critical for response to low-nitrogen stress in tomato.

Then, we measured nitrogen and carbon contents in the shoots and roots of these plants. Under nitrogen-sufficient conditions, there were no significant differences in nitrogen or carbon contents among the roots of WT, *snrk1α1* mutants, and *SnRK1α1* OE plants (Fig. 1F). However, after 3 weeks of low nitrogen treatment, the nitrogen and carbon contents in the *snrk1α1* mutant roots decreased significantly compared to WT, while *SnRK1α1* OE plants had higher nitrogen and carbon contents than WT (Fig. 1F). A similar pattern was observed for the carbon and nitrogen contents of the shoots (Supplementary Fig. S4). Furthermore, we evaluated the transcript levels of nitrate transporter genes *NRT1.1* and *NRT2.1* in the roots. While the expression of *NRT1.1* and *NRT2.1* in WT, *snrk1α1*, and *SnRK1α1* OE roots did not differ significantly under sufficient nitrogen conditions, both transporter genes were significantly upregulated in WT plants after 3 d of low nitrogen treatment (Fig. 1G). The induction of *NRT1.1* and *NRT2.1* was lower in *snrk1α1* mutants than in WT and further elevated in *SnRK1α1* OE plants (Fig. 1G).

To investigate if ROS are produced during nitrogen starvation, we used DAB and DCF staining to reveal a marked increase in ROS accumulation in WT roots after 1 d of low nitrogen treatment (Fig. 1, H and I). Additionally, we found that ROS accumulation was decreased in *snrk1α1* mutants but increased in *SnRK1α1* OE lines compared to WT (Fig. 1, H and I). To further explore the low nitrogen stress–induced subcellular localization of ROS generation, we employed a CeCl_3_-based procedure to detect apoplastic H_2_O_2_, the main species of ROS. We discovered that low-nitrogen stress triggers the accumulation of apoplastic H_2_O_2_ in the root of WT plants. Apoplastic H_2_O_2_ accumulation was significantly higher in *SnRK1α1* OE roots and significantly lower in *snrk1α1* (Fig. 1J). A quantitative analysis of H_2_O_2_ content supported these observations, confirming SnRK1α1-mediated ROS accumulation in roots following low nitrogen stress (Fig. 1K). Apoplastic H_2_O_2_ is produced by NADPH oxidases at the cell membrane (Zhou et al. 2014a, 2014b). Thus, we examined NADPH oxidase activity in the roots after low-nitrogen stress. Low nitrogen conditions induced NADPH oxidase activity in WT roots; this activity was further enhanced in *SnRK1α1* OE but suppressed in *snrk1α1* (Fig. 1L). These results suggest that RBOHs/NADPH oxidase-dependent ROS may be crucial in SnRK1α1-mediated tolerance to low nitrogen stress.

### Role of RBOH1-dependent ROS in tomato responses to low nitrogen stress

**Figure 2.**
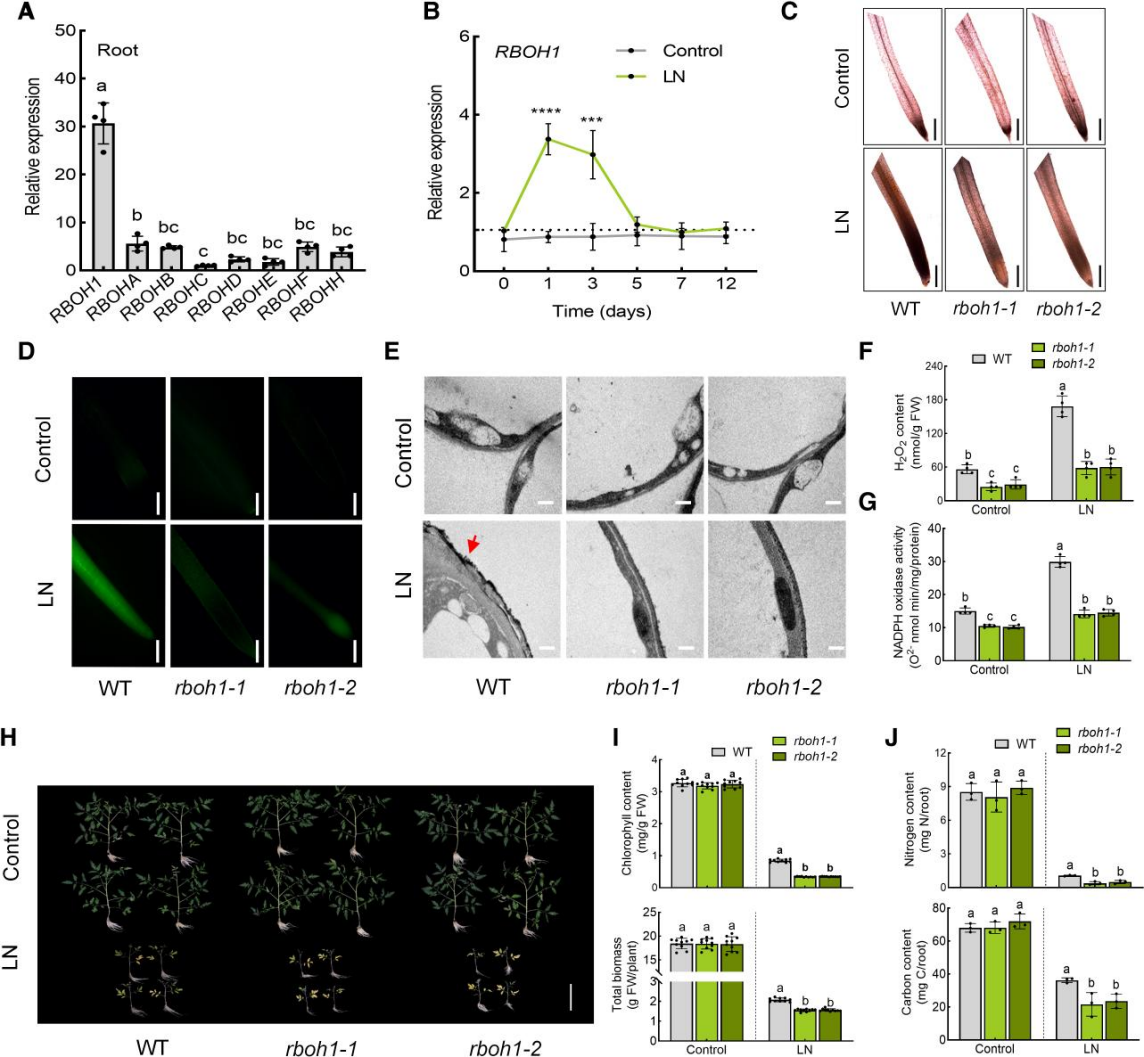
The effect of RBOH1 on tomato response to low nitrogen stress. **A)** Expression of *RBOH*s (*RBOH1*, *RBOHA*, *RBOHB*, *RBOHC*, *RBOHD*, *RBOHE*, *RBOHF*, *RBOHH*) in roots under normal conditions. Relative expression of *RBOH*s in roots is shown, with *RBOHC* set to “1”. **B)** Expression of *RBOH1* under control and low nitrogen conditions. The relative expression of wild type (WT) under low nitrogen conditions at 0 d was set to “1”. **C)***In situ* detection of ROS by 3,3′-diaminobenzidine (DAB) staining of roots. Bars: 300 *μ*m. Images were digitally extracted for comparison. **D)** H_2_DCF-DA fluorescence (DCF) staining of roots. Bars: 300 *μ*m. **E)** Detection of apoplastic H_2_O_2_ by CeCl_3_ staining in the roots. Bars: 0.5 *μ*m. Arrows indicate the accumulation of H_2_O_2_. **F)** H_2_O_2_ content and **(G)** NADPH oxidase activity in the roots of wild type (WT) and *rboh1* mutants under low nitrogen stress. **H)** Phenotypes of WT and *rboh1* mutants under low nitrogen stress. Bars: 10 cm. Images were digitally extracted for comparison. **I)** Chlorophyll content (upper panel) and biomass (lower panel) of WT and *rboh1* mutants under low nitrogen stress. **J)** Nitrogen (upper panel) and carbon (lower panel) contents in the roots of WT and *rboh1* mutants under low nitrogen stress. Contents were calculated by multiplying the dry weight of the roots with their nitrogen and carbon concentrations. Error bars represent SD; data are means ± SD of 3 biological replicates in **(J)**, 4 biological replicates in **(A, B, F, G)**, and 10 biological replicates in **(C, D, E, H, I)**. Experiments were repeated three times with similar results. Distinct letters above the bars signify significant differences at the *P* < 0.05 level, as determined by one-way ANOVA **(A, I, J)** and two-way ANOVA **(F, G)** with Tukey's multiple comparison test. Asterisks above the bars represent significant differences by Student's *t*-tests in **(B)**. ****, *P* < 0.001; ***, *P* < 0.001. Precise *P*-values from these statistical tests are detailed in the Supplementary Data Set 10.

To elucidate the role of RBOHs/NADPH oxidase-dependent ROS in tomato response to low-nitrogen stress, we examined the expression of all 8 tomato *RBOH* genes in the roots (Fig. 2A). Under normal conditions, *RBOH1* was highly transcribed compared to the other seven *RBOH* genes in tomato roots. Furthermore, *RBOH1* expression was significantly higher in tomato roots and flowers compared to other tissues, highlighting its role in tissue-specific ROS production (Yan et al. 2020). Under low-nitrogen stress, *RBOH1* expression was significantly induced 1 to 3 d into treatment (Fig. 2B). Consequently, we generated *RBOH1* knockout mutants (*rboh1-1* and *rboh1-2*) to investigate the role of ROS production on low-nitrogen stress tolerance (Supplementary Fig. S5). Following low-nitrogen stress, ROS accumulation was significantly diminished in *rboh1* compared to WT roots (Fig. 2, C to E). Consistently, low nitrogen-induced H_2_O_2_ production and NADPH oxidase activity were compromised in the roots of *rboh1* plants (Fig. 2, F and G), suggesting that RBOH1 mediates ROS production under low-nitrogen stress. We further examined the function of RBOH1 in tomato during low nitrogen. Under normal nitrogen conditions, no noticeable differences in growth phenotypes were observed between WT and *rboh1* mutants (Fig. 2H). However, after 3 weeks of low nitrogen treatment, *rboh1* mutants displayed severe leaf yellowing, compared to WT plants, which exhibited mild chlorosis (Fig. 2H). The chlorophyll content and total biomass of *rboh1* mutants were significantly lower than those of WT plants under low nitrogen stress (Fig. 2I). Under normal conditions, the nitrogen and carbon contents of both roots and shoots were similar between WT and *rboh1* plants (Fig. 2J and Supplementary Fig. S6), but low nitrogen led to lower nitrogen and carbon contents in the roots and shoots of *rboh1* mutants compared to WT (Fig. 2J and Supplementary Fig. S6), highlighting the crucial role of RBOH1-dependent ROS in the responses to low-nitrogen stress.

**Figure 3.**
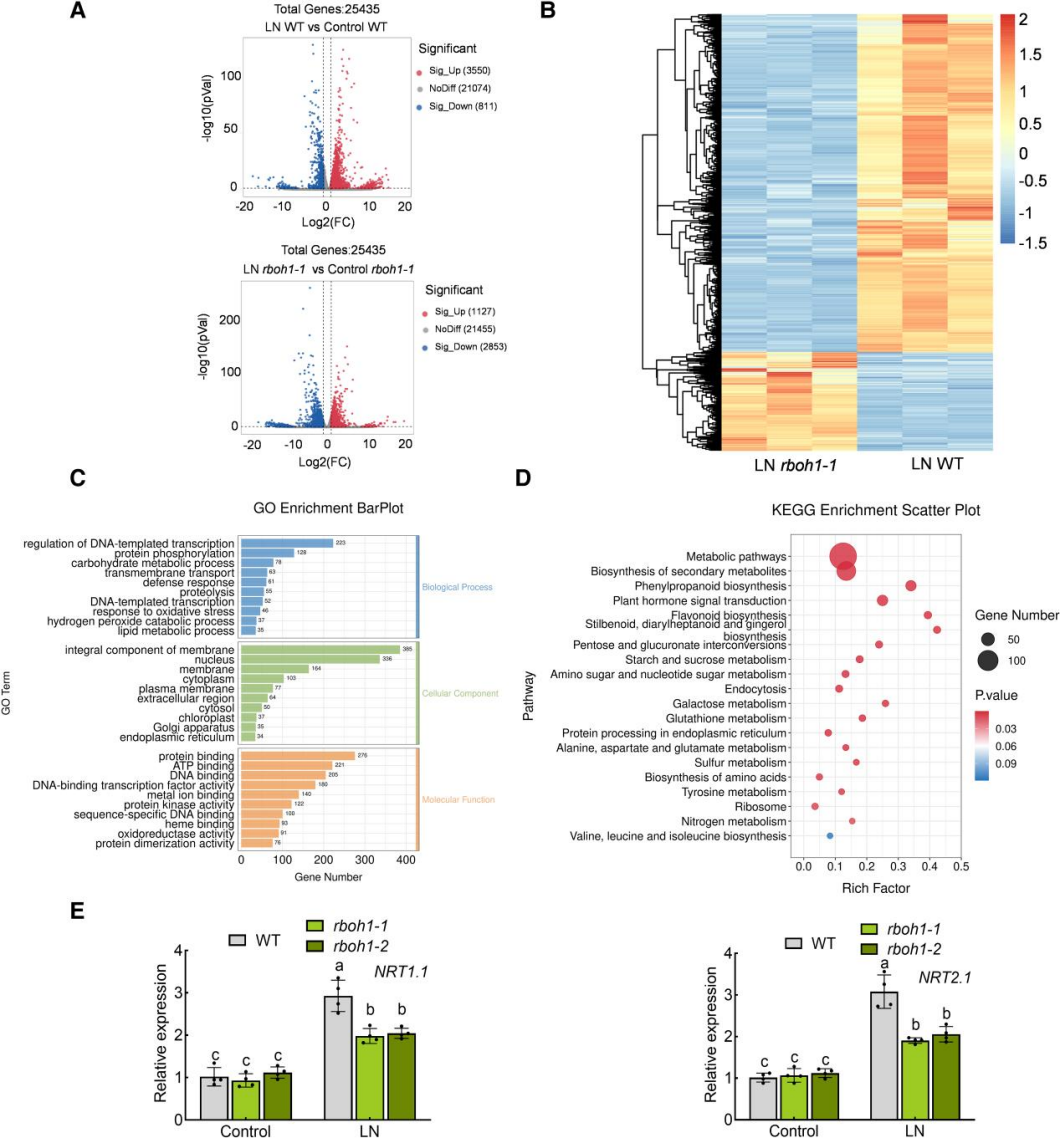
RNA-seq analysis of wild type (WT) and *rboh1* roots under low nitrogen stress. **A)** Volcano plot of differentially expressed genes (DEGs) between WT and *rboh1* under low nitrogen stress. **B)** Heatmap of DEGs between WT and *rboh1* under low nitrogen stress. The color scale indicates different log_2_ (FPKM) values of DEGs. **C)** Gene ontology (GO) annotation and categorization of genes downregulated in *rboh1* mutants compared to WT under low nitrogen stress. **D)** Kyoto Encyclopedia of Genes and Genomes (KEGG) enrichment analysis of genes downregulated in *rboh1* mutants compared to WT under low nitrogen stress. **E)** Gene expression of *NRT1.1* (left panel) and *NRT2.1* (right panel) in the roots. The relative expression of WT under control conditions was set to “1”. Error bars represent SD; data are means ± SD of *n* = 4 biological replicates in **(E)**. Experiments were repeated three times in **(E)** with similar results. Distinct letters above the bars signify significant differences at the *P* < 0.05 level, as determined by two-way ANOVA **(E)** with Tukey's multiple comparison test. Data are provided in the Supplementary Data Sets 1 to 4 and 6. Precise *P*-values from these statistical tests are detailed in the Supplementary Data Set 10.

To investigate the extensive transcriptome changes induced by RBOH1-dependent ROS under low-nitrogen stress, we conducted transcriptome deep sequencing (RNA-seq) on both WT and *rboh1-1* mutants. After 3 d of low nitrogen treatment, root samples were collected from each condition, and RNA was extracted for transcriptome sequencing. In WT roots, 3,550 genes were significantly upregulated, while only 1,127 genes were upregulated in *rboh1* mutants after low nitrogen treatment (Fig. 3A, Supplementary Data Set 1 and S2). Conversely, 811 genes were downregulated in WT roots under low-nitrogen stress, compared to 2,853 genes in *rboh1-1* mutants (Fig. 3A, Supplementary Data Set 1 and 2). These results indicated that the loss of RBOH1 function affects the expression pattern of genes associated with the low nitrogen response. Comparing low-nitrogen (LN) *rboh1-1* with LN WT, 1,123 genes were expressed significantly higher in *rboh1-1* mutants, while 3,670 genes were expressed significantly lower (Fig. 3B, Supplementary Fig. S7A and Supplementary Data Set 3). We performed gene ontology (GO) functional annotation on these 3,670 differentially expressed genes (DEGs). GO functional annotation revealed that these DEGs are involved many cellular functions and biological processes, including transcription, translation, protein phosphorylation, carbon metabolism, and membrane transport processes (Fig. 3C, Supplementary Data Set 4). Within the enriched GO process, “membrane transport,” six nitrate transporter genes (*NRT*s) related to nitrate uptake were upregulated. *NRT1.1* and *NRT2.1* exhibited significantly decreased expression levels in *rboh1* than WT under low nitrogen conditions (*P* < 0.05) (Supplementary Fig. S8, Supplementary Data Set 5). Notably, *NRT1.1* and *NRT2.1* exhibited higher expression in both WT and *rboh1-1*, compared to other *NRT*s (Supplementary Fig. S8). Kyoto Encyclopedia of Genes and Genomes (KEGG) pathway analysis of the DEGs also suggested that RBOH1-dependent ROS might be involved in nitrogen metabolism, various amino acid metabolic processes, and amino acid biosynthesis under low-nitrogen stress (Fig. 3D, Supplementary Data Set 6). We selected *NRT1.1* and *NRT2.1* for RT-qPCR validation. As shown in Fig. 3E, after 3 d of low nitrogen treatment, the expression levels of *NRT1.1* and *NRT2.1* were significantly upregulated in WT plants. However, this induction was compromised in *rboh1* mutants compared to WT, aligning with the transcriptomic data (Fig. 3E). The combined results from GO enrichment analysis and KEGG analysis indicate that RBOH1-dependent ROS play a critical role in influencing nitrate uptake via NRTs, nitrogen metabolism, and various amino acid metabolic processes under low nitrogen stress.

In addition, we compared Control *rboh1-1* and Control WT and found that *rboh1-1* has 1,804 significantly higher and 239 significantly lower expressed genes than WT under control conditions. GO enrichment and KEGG analysis of these 2,043 genes revealed associations with the plasma membrane, cell wall, hydrolase activity, and secondary metabolite synthesis (Supplementary Fig. S7, Supplementary Data Set 7 to 9). This comprehensive analysis highlights the role of RBOH1 under stress and during plant homeostasis.

To ascertain the pivotal role of RBOH1-dependent ROS production in tomato responses to low-nitrogen stress, we subjected WT plants to varying concentrations of H_2_O_2_. Under normal nitrogen conditions, plant growth was largely unaffected by H_2_O_2_ concentrations below 1 mM (Supplementary Fig. S9). However, higher H_2_O_2_ concentrations (≥5 mM) caused significant stress symptoms, such as leaf yellowing, decreased chlorophyll contents and biomass (Supplementary Fig. S9). Under low nitrogen stress, moderate H_2_O_2_ applications (500 *µ*M) improved stress symptoms associated low nitrogen (Supplementary Fig. S9). We also examined the effect of H_2_O_2_ treatment on tolerance to low nitrogen in *snrk1α1* and *rboh1* mutants (Supplementary Fig. S10). We found that 500 *µ*M H_2_O_2_ decreased stress-associated phenotypes in the *rboh1* mutant (chlorophyll content, biomass, nitrogen, and carbon content), but could not fully rescue *snrk1α1* mutants under low nitrogen stress, suggesting that while RBOH1-dependent ROS are critical for tomato tolerance to low nitrogen, SnRK1α1 may facilitate nitrogen uptake through additional pathways.

### RBOH1 acts downstream of SnRK1α1 in tomato response to low nitrogen stress

**Figure 4.**
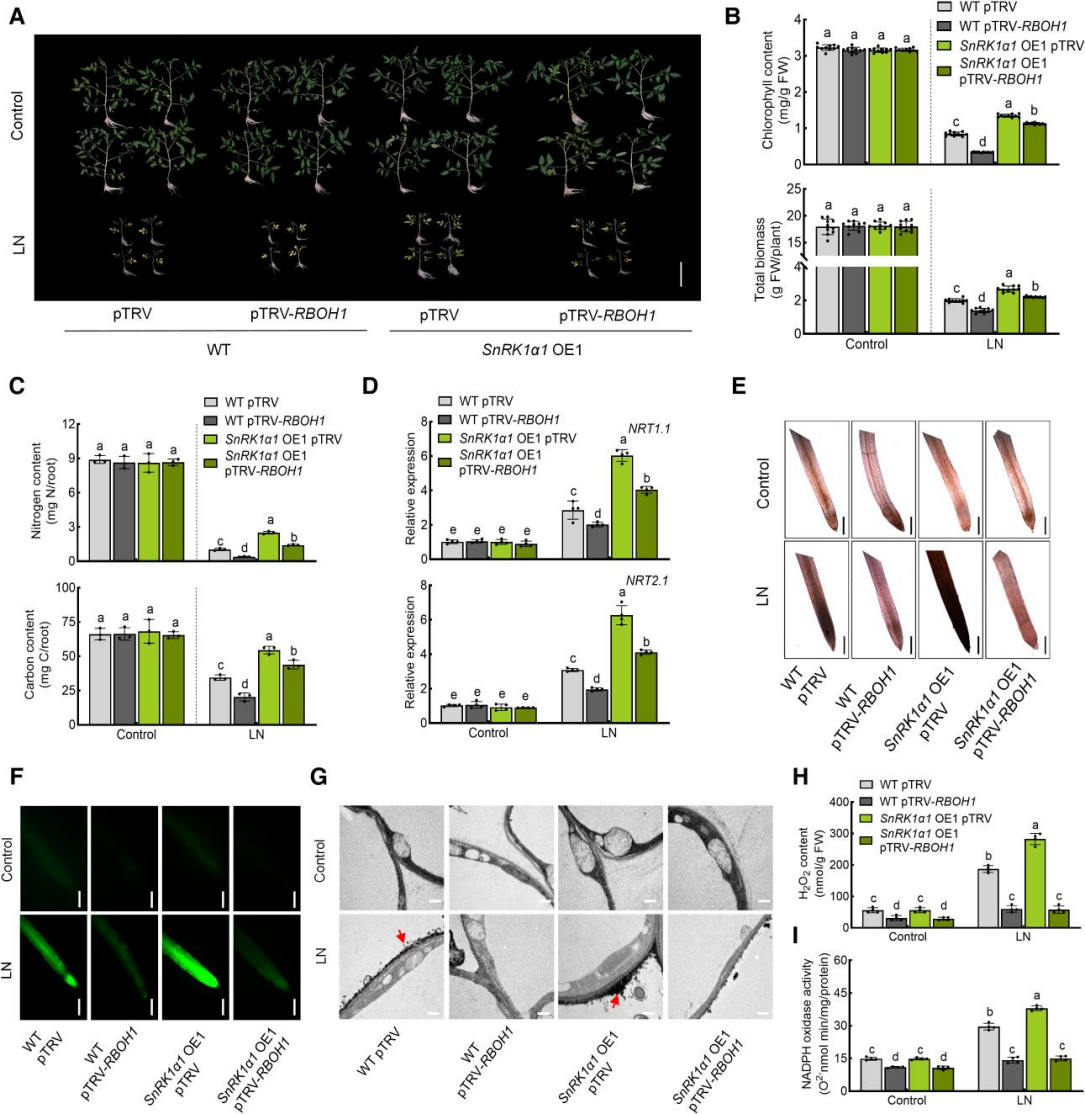
SnRK1α1 acts upstream of RBOH1. Silencing of *RBOH1* in wild type (WT) and *SnRK1α1* OE1 plants. **A)** Phenotypes of plants under low nitrogen stress in different genotypes of tomato plants. Bars: 10 cm. Images were digitally extracted for comparison. **B)** Chlorophyll content (upper panel) and biomass (lower panel) of plants under low nitrogen stress in different genotypes. **C)** Nitrogen (upper panel) and carbon (lower panel) contents in the roots of plants under low nitrogen stress in different genotypes. The contents were calculated by multiplying the dry weight of the roots with their nitrogen and carbon concentrations. **D)** Gene expression of *NRT1.1* (upper panel) and *NRT2.1* (lower panel) in the roots under low nitrogen stress in different genotypes. The relative expression of WT pTRV under control conditions was set to “1”. **E)** In situ detection of ROS by 3,3′-diaminobenzidine (DAB) staining of roots. Bars: 300 *μ*m. Images were digitally extracted for comparison. **F)** H_2_DCF-DA fluorescence (DCF) staining of roots. Bars: 300 *μ*m. **G)** Detection of apoplastic H_2_O_2_ by CeCl_3_ staining in the roots. Bars: 0.5 *μ*m. Arrows indicate the accumulation of H_2_O_2_. **H)** H_2_O_2_ content and **(I)** NADPH oxidase activity in the roots of different lines under low nitrogen stress. Error bars represent SD; data are means ± SD of 3 biological replicates in **(C)**, 4 biological replicates in **(D, H, I)**, and 10 biological replicates in **(A, B, E, F)**. Experiments were repeated three times with similar results. Distinct letters above the bars signify significant differences at the *P* < 0.05 level, as determined by one-way ANOVA **(B, C)** and two-way ANOVA **(D, H, I)** with Tukey's multiple comparison test. Precise *P*-values from these statistical tests are detailed in the Supplementary Data Set 10.

To investigate the genetic interplay between SnRK1α1 and RBOH1, we employed the virus-induced gene silencing (VIGS) technique to downregulate *RBOH1* expression in both WT and *SnRK1α1* OE1 plants. Silencing *RBOH1* led to a 70% to 80% drop in *RBOH1* transcript levels in both WT and *SnRK1α* OE1 plants (Supplementary Fig. S11A). Consistent with the observations in *rboh1* mutants, silencing *RBOH1* diminished tolerance to low nitrogen stress, along with suppressed expression of *NRT1.1* and *NRT2.1* (Fig. 4, A to D and Supplementary Fig. S11). Interestingly, silencing *RBOH1* partially compromised SnRK1α1-mediated low nitrogen tolerance in *SnRK1α1* OE lines (Fig. 4 and Supplementary Fig. S11). Additionally, we observed that the promotion of ROS production by SnRK1α1 is dependent on RBOH1, as demonstrated through DAB, DCF, and CeCl_3_ staining of roots (Fig. 4, E to G). Quantitative determination of H_2_O_2_ content in the root confirmed this finding (Fig. 4H). NADPH oxidase activity in *SnRK1α1* OE lines was significantly lower due to *RBOH1* gene silencing (Fig. 4I). These results suggest that RBOH1 acts downstream of SnRK1α1 in tomato tolerance to low nitrogen stress, with SnRK1α1 promoting ROS production in a manner dependent on RBOH1 under low nitrogen conditions.

### SnRK1α1 interacts with and phosphorylates RBOH1

**Figure 5.**
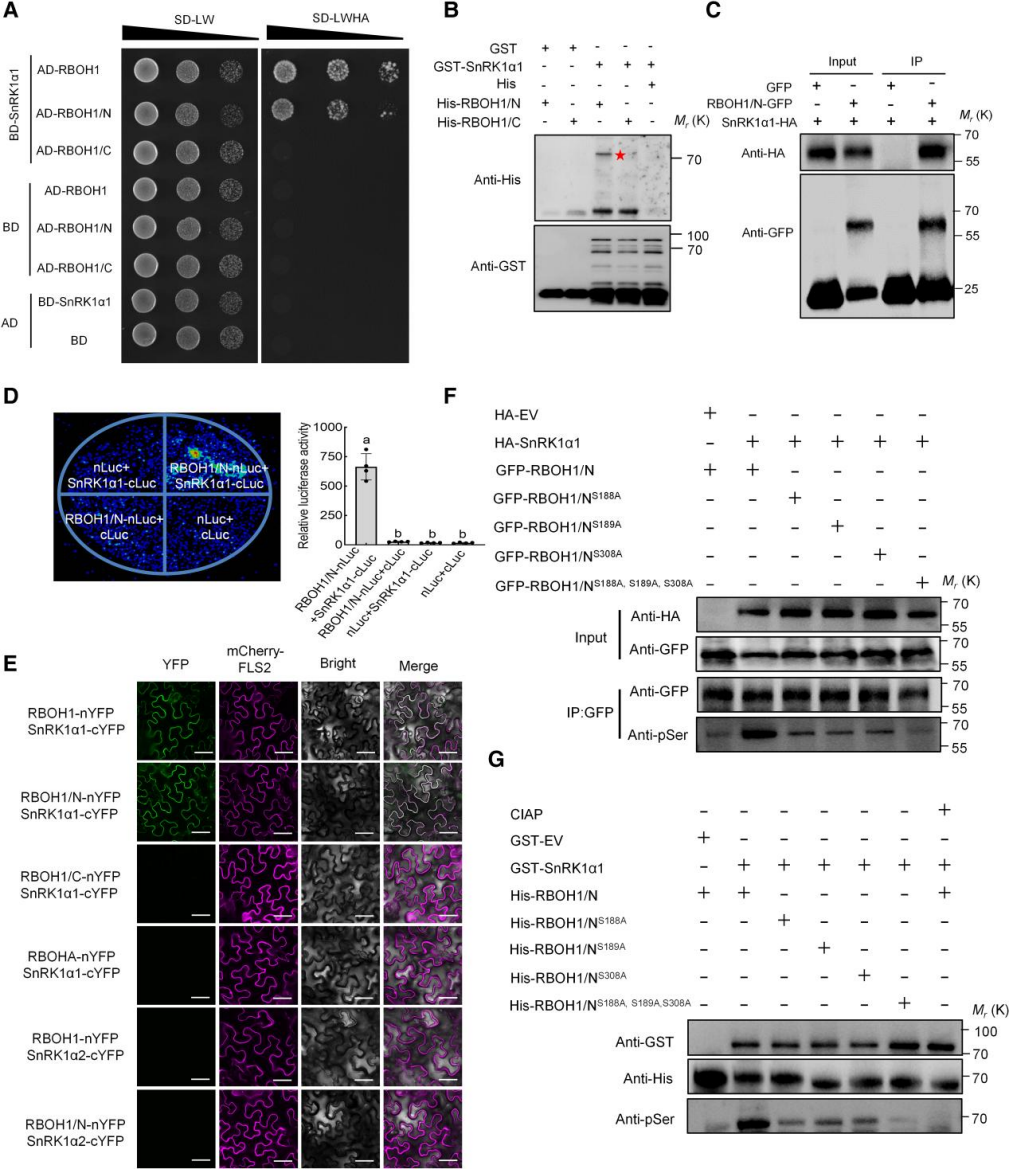
SnRK1α1 interacts with and phosphorylates RBOH1 in vivo and in vitro. **A)** Yeast two-hybrid assays confirming the interaction between SnRK1α1 and RBOH1 or the N terminus of RBOH1 (RBOH1/N). Yeast grown on SD/-Leu/-Trp (-LW) for 2 d or SD/-Leu/-Trp/-Ade/-His (-LWAH) medium for 5 d. **B)** GST pull-down assays showing the interaction between SnRK1α1 and RBOH1/N. GST protein as negative control. The star indicates His-RBOH1/N. Molecular weight (kDa) markers are on the left side of the blots. **C)** Co-immunoprecipitation (Co-IP) assays showing the SnRK1α1-RBOH1/N interaction in *N. benthamiana* leaves. The immunoprecipitated products were detected with anti-HA and anti-GFP antibodies, respectively. **D)** Split luciferase complementation assays showing the interaction between SnRK1α1 and RBOH1/N. RBOH1/N-nLUC and SnRK1α1-cLUC constructs co-transformed into *N. benthamiana* leaves, LUC signal detected after 48 h. Left panel shows representative pictures, right panel shows luciferase activity. **E)** Bimolecular fluorescence complementation (BiFC) assays showing interaction between SnRK1α1 and RBOH1. Full-length RBOH1, RBOHA, RBOH1/N, and the C terminus of RBOH1 (RBOH1/C) were fused to nYFP fragments, while SnRK1α1 and SnRK1α2 were fused to cYFP fragments, respectively. mCherry-FLS2 fusion protein served as a marker for plasma membrane localization. Bars: 25 *μ*m. **F)** In vivo phosphorylation of RBOH1/N mediated by SnRK1α1. RBOH1/N-GFP and its variants (RBOH1/N^S188A^-GFP, RBOH1/N^S189A^-GFP, RBOH1/N^S308A^-GFP, RBOH1/N^S188A, S189A, S308A^-GFP) were co-expressed with SnRK1α1-HA (empty vector as a negative control) in *N. benthamiana* leaves. Phosphorylated RBOH1/N and its variants were precipitated by GFP-Trap, and immunoblotted with anti-phospho-serine (anti-pSer) antibody. **G)** In vitro phosphorylation of RBOH1/N mediated by SnRK1α1. Recombinant GST-SnRK1α1 (empty vector as a negative control) was incubated with various His-tagged RBOH1/N and its variants in reaction buffer. Phosphorylated proteins were detected by anti-pSer antibody after separation by SDS-PAGE. Error bars represent SD; data are means ± SD of 4 biological replicates in **(D)**. Experiments were repeated three times with similar results. Distinct letters above the bars signify significant differences at the *P* < 0.05 level, as determined by one-way ANOVA **(D)** with Tukey's multiple comparison test. Precise *P*-values from these statistical tests are detailed in the Supplementary Data Set 10.

To explore the relationship between SnRK1α1 and RBOHs, we conducted yeast two-hybrid (Y2H) assays to investigate potential interactions between SnRK1α1 and 8 tomato RBOH homologous proteins and found that SnRK1α1 specifically interacts with RBOH1 (Supplementary Fig. S12A). To identify which domain of RBOH1 interacts with SnRK1α1, we divided RBOH1 into its N-terminal domain (RBOH1/N) and its C-terminal domain (RBOH1/C) and performed further assays (Supplementary Fig. S12B). The N-terminal domain of RBOH1 interacted with SnRK1α1 (Fig. 5A). We validated this interaction using GST pull-down assays with recombinant GST-SnRK1α1, His-RBOH1/N, and His-RBOH1/C proteins. GST-SnRK1α1 was able to co-precipitate with His-RBOH1/N but not with His or His-RBOH1/C (Fig. 5B), confirming a direct interaction between SnRK1α1 and the N-terminal domain of RBOH1. This interaction was further supported by co-immunoprecipitation (Co-IP) experiments, where SnRK1α1-HA was detected in the immunoprecipitated GFP-RBOH1/N complex, but not with GFP alone (Fig. 5C). We also examined this interaction in vivo using split luciferase complementation assays in *Nicotiana benthamiana* leaves. Co-expression of *SnRK1α1-cLUC* and *RBOH1/N-nLUC* resulted in strong luminescent signals, indicating that SnRK1α1 and RBOH1/N interact in vivo (Fig. 5D). To determine the subcellular localization of the interaction, we used a mCherry-FLS2 fusion protein as a marker for plasma membrane localization. The bimolecular fluorescence complementation (BiFC) signal showed that RBOH1/N-nYFP and cYFP-SnRK1α1 interact, as evidenced by the colocalization of the YFP and mCherry fluorescence signals at the plasma membrane (Fig. 5E). To test for specificity, BiFC interactions were also examined between RBOH1/C-nYFP and cYFP-SnRK1α1, RBOHA-nYFP and cYFP-SnRK1α1, RBOH1-nYFP and cYFP-SnRK1α2, as well as RBOH1/N-nYFP and cYFP-SnRK1α2, where RBOHA is closely related to RBOH1 and SnRK1α2 is closely related to SnRK1α1 (Fig. 5E). None of these combinations showed interaction at the plasma membrane (Fig. 5E). These results were confirmed by Y2H assays (Supplementary Fig. S13).

Given that post-translational modifications, such as phosphorylation, play a critical role in RBOH functionality, and considering the role of SnRK1α1 as a kinase in plant responses to nutritional stress, we hypothesized that RBOH1 is a phosphorylation target of SnRK1α1. In vitro phosphorylation analysis using recombinant His-RBOH1/N and GST-SnRK1α1 identified 3 potential SnRK1α1 phosphorylation sites on RBOH1: S188, S189, and S308 (Supplementary Fig. S14). To validate these findings in vivo, we co-expressed *SnRK1α1* and *RBOH1/N* in *N. benthamiana* leaves and performed immunoblotting with an anti-pSer antibody. We detected increased levels of phosphorylated RBOH1/N in the presence of SnRK1α1 (Fig. 5F). We further generated RBOH1/N variants with the serine residues mutated to nonphosphorylatable alanine (RBOH1/N^S188A^, RBOH1/N^S189A^, RBOH1/N^S308A^, and a triple variant RBOH1/N^S188A, S189A, S308A^) and observed significantly lower phosphorylation levels, with the triple mutant showing the most substantial decrease (Fig. 5F). In vitro phosphorylation assays confirmed that SnRK1α1 phosphorylates RBOH1/N, and that this phosphorylation dramatically declined in the presence of phosphatase (CIAP) (Fig. 5G). Despite these phosphorylation site mutations, the interaction between SnRK1α1 and RBOH1/N remained unaffected in Y2H assays (Supplementary Fig. S15). Overall, our results indicate that SnRK1α1 interacts with and phosphorylates RBOH1, specifically targeting serine residues S188, S189, and S308.

### SnRK1α1-dependent phosphorylation of RBOH1 is essential for ROS production and tomato tolerance under low nitrogen stress

**Figure 6.**
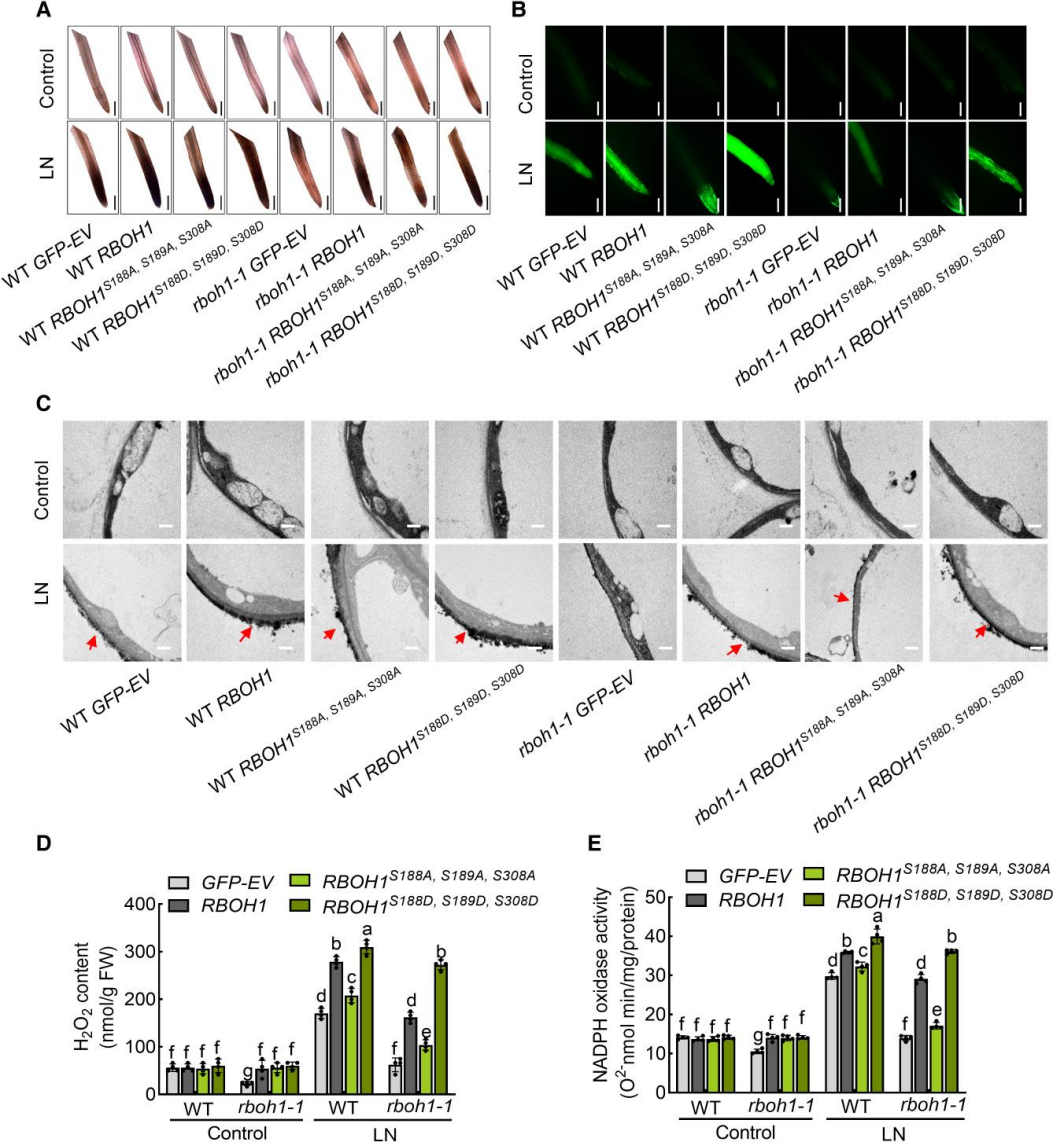
Phosphorylation of RBOH1 by SnRK1α1 activates ROS production. **A)** In situ detection of ROS by 3,3′-diaminobenzidine (DAB) and **(B)** H_2_DCF-DA fluorescence (DCF) staining in the roots of different transgenic plants: *proRBOH1:GFP-EV* (empty vector) (*GFP-EV*), *proRBOH1:GFP-RBOH1* (*RBOH1*), *proRBOH1:GFP-RBOH1^S188A, S189A, S308A^* (*RBOH1^S188A, S189A, S308A^*) and phospho-mimic variants *proRBOH1:GFP-RBOH1^S188D, S189D, S308D^* (*RBOH1^S188D, S189D, S308D^*) in WT and *rboh1-1* mutants. Bars: 300 *μ*m. Images were digitally extracted for comparison in **(A)**. **C)** Detection of apoplastic H_2_O_2_ by CeCl_3_ staining in the roots of different transgenic plants. Bars: 0.5 *μ*m. Arrows indicate the accumulation of H_2_O_2_. **D)** H_2_O_2_ content and **(E)** NADPH oxidase activity in the roots of different transgenic tomato plants under low nitrogen stress. Error bars represent SD; data are means ± SD of 4 biological replicates in **(D, E)**, 10 biological replicates in **(A, B, C)**. Experiments were repeated three times with similar results. Distinct letters above the bars signify significant differences at the *P* < 0.05 level, as determined by two-way ANOVA **(D, E)** with Tukey's multiple comparison test. Precise *P*-values from these statistical tests are detailed in the Supplementary Data Set 10.

To further elucidate the role of RBOH1 serine phosphorylation in ROS production and plant tolerance under low nitrogen stress, we generated transgenic tomato lines in WT and *rboh1* containing the *RBOH1* gene or its mutated variants driven by its own promoter. The transgenic lines included: *proRBOH1:GFP* (*GFP*-*EV*), *proRBOH1:GFP-RBOH1* (*RBOH1*), the nonphosphorylatable *RBOH1* variant, *proRBOH1:GFP-RBOH1^S188A, S189A, S308A^* (*RBOH1^S188A, S189A, S308A^*), and the phospho-mimic variant where serine residues were substituted with aspartate, *proRBOH1:GFP-RBOH1^S188D, S189D, S308D^* (*RBOH1^S188D, S189D, S308D^*). GFP signals were consistent under normal conditions and increased under low nitrogen conditions in all *proRBOH1*-driven WT lines (Supplementary Fig. S16A). With similar GFP abundance among the transgenic lines, we next assessed ROS production. When introducing the *RBOH1* gene in the *rboh1* mutant (*rboh1 RBOH1*), we were able to significantly increase ROS production and NADPH oxidase activity under low nitrogen stress compared to the *GFP-EV* control (*rboh1 GFP-EV*; Fig. 6). Notably, while ROS production and NADPH oxidase activity were lower in the *rboh1 RBOH1^S188A, S189A, S308A^* lines, where serine residues could not be phosphorylated, they were increased in the *rboh1 RBOH1^S188D, S189D, S308D^* lines (Fig. 6).

**Figure 7.**
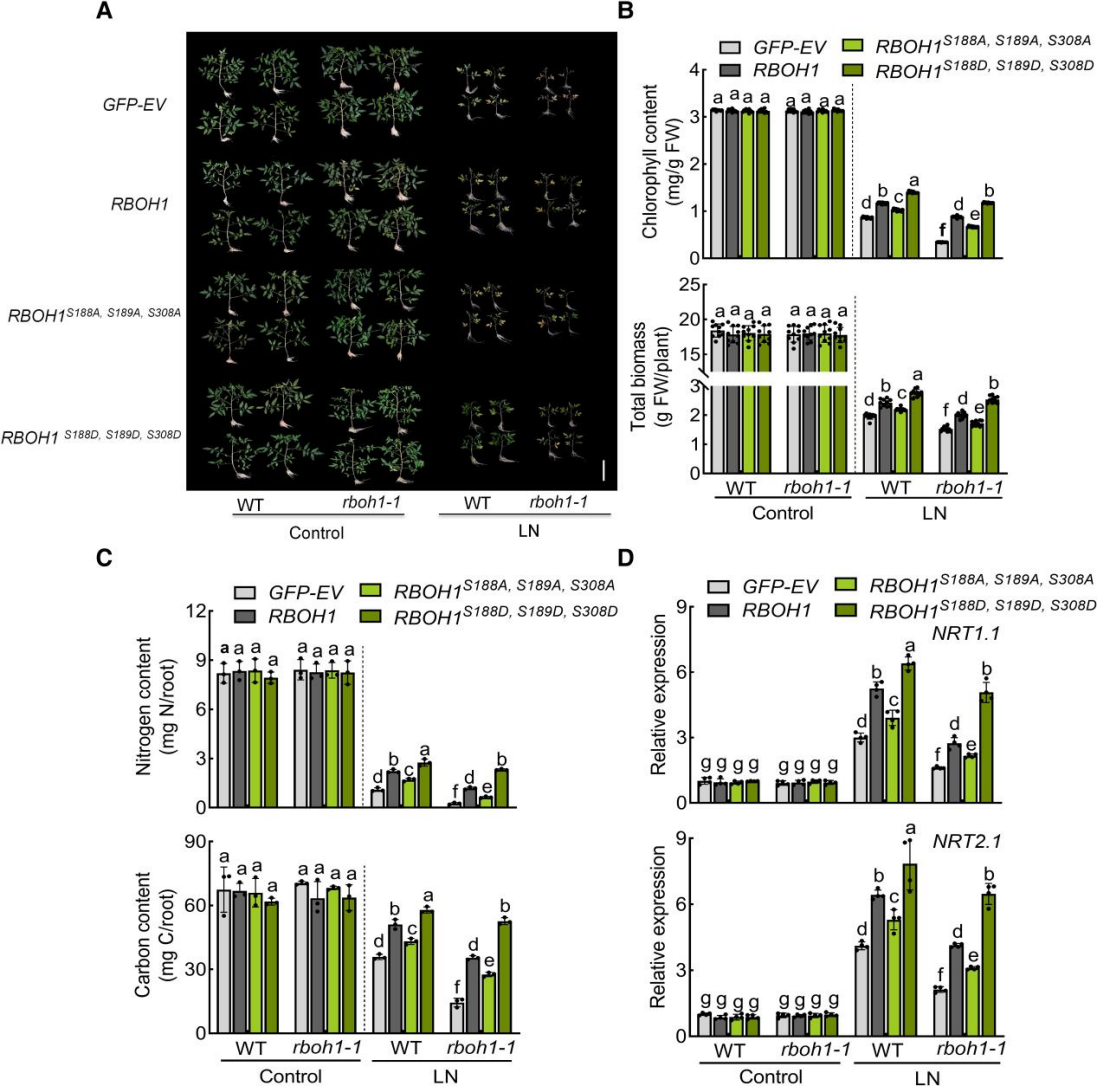
Phosphorylation of RBOH1 by SnRK1α1 enhances tolerance to low nitrogen stress. **A)** Phenotypes of different transgenic plants: *proRBOH1:GFP-EV* (empty vector) (*GFP-EV*), *proRBOH1:GFP-RBOH1* (*RBOH1*), *proRBOH1:GFP-RBOH1^S188A, S189A, S308A^* (*RBOH1^S188A, S189A, S308A^*) and phospho-mimic variants *proRBOH1:GFP-RBOH1^S188D, S189D, S308D^* (*RBOH1^S188D, S189D, S308D^*) in WT and *rboh1-1* mutants under low nitrogen stress. Bars: 10 cm. Images were digitally extracted for comparison. **B)** Chlorophyll content (upper panel) and biomass (lower panel) of different transgenic plants under low nitrogen stress. **C)** Nitrogen (upper panel) and carbon (lower panel) contents in the roots of different transgenic plants under low nitrogen stress. The contents were calculated by multiplying the dry weight of the aerial parts with their nitrogen and carbon concentrations. **D)** Gene expression of *NRT1.1* (upper panel) and *NRT2.1* (lower panel) in the roots of different transgenic plants under low nitrogen stress. The relative expression of WT *GFP-EV* under control conditions was set to “1”. Error bars represent SD; data are means ± SD of 3 biological replicates in **(C)**, 4 biological replicates in **(D)**, 10 biological replicates in **(A, B)**. Experiments were repeated three times with similar results. Distinct letters above the bars signify significant differences at the *P* < 0.05 level, as determined by one-way ANOVA **(B, C)** and two-way ANOVA analysis **(D)** with Tukey's multiple comparison test. Precise *P*-values from these statistical tests are detailed in the Supplementary Data Set 10.

Phenotypic analysis of these lines under low nitrogen stress revealed that replacing *RBOH1* in the *rboh1* mutants (*rboh1 RBOH1*) largely mitigated the phenotypic defects of the *rboh1* mutants by increasing chlorophyll content, biomass, nitrogen content, carbon content, and the expression of *NRT1.1* and *NRT2.1*, to levels similar to WT (Fig. 7). Furthermore, *rboh1 RBOH1^S188A, S189A, S308A^* plants exhibited decreased nitrate uptake and low nitrogen tolerance, while *rboh1 RBOH1^S188D, S189D, S308D^* plants showed increased nitrate uptake and improved low nitrogen tolerance compared to *rboh1 RBOH1* plants (Fig. 7 and Supplementary Fig. S16B). These results further support the notion that phosphorylation at S188, S189, and S308 of RBOH1 is crucial for ROS production, NADPH oxidase activity, nitrate uptake, and low nitrogen tolerance in tomato roots.

### H_2_O_2_-mediated TGA4 oxidation promotes the expression of *NRT1.1* and *NRT2.1*

**Figure 8.**
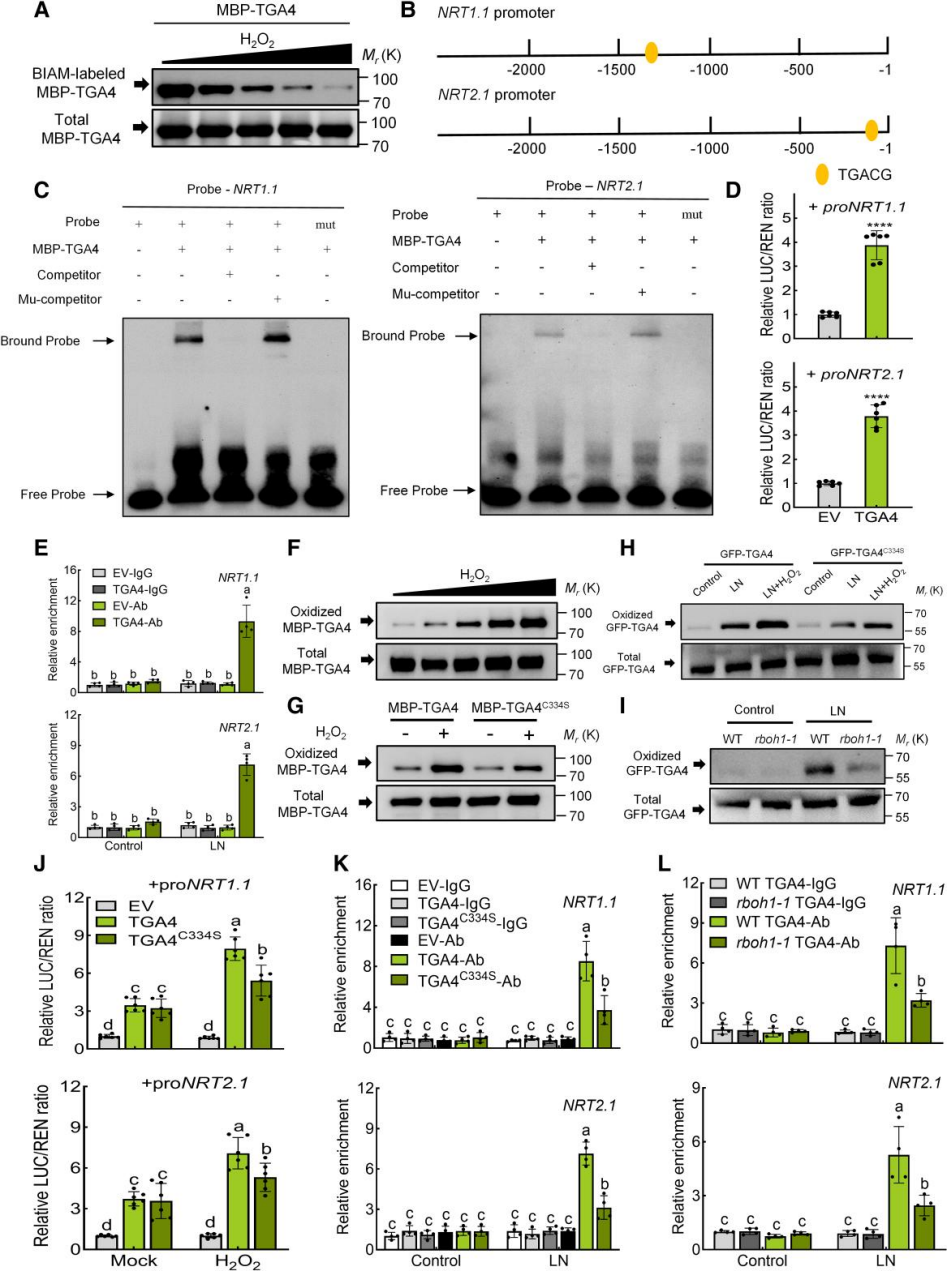
The oxidation of TGA4 by RBOH1-induced H_2_O_2_ promotes the expression of *NRT1.1* and *NRT2.1*. **A)** Oxidative modification of TGA4 was analyzed using the BIAM labeling assay. MBP-TGA4 protein was treated with different H_2_O_2_ concentrations (0 *μ*M, 1 *μ*M, 10 *μ*M, 100 *μ*M, 1 mM), labeled with BIAM and detected by immunoblotting with HRP-conjugated streptavidin and anti-MBP antibodies. **B)** Diagram showing the TGA4 binding site “TGACG” within the 2,000 bp regions of *NRT1.1* and *NRT2.1* promoters. **C)** EMSA testing the binding of MBP-TGA4 to probes from *NRT1.1* and *NRT2.1* promoters containing the TGACG motif. Mutated probes (TGACG to AAAAA) and competitors were also tested. Competitors and mutant competitors (Mu-competitor) were used at 1,000-fold. **D)** Dual-luciferase assays determined TGA4's regulatory effects on *NRT1.1* and *NRT2.1* promoters. The ratios of firefly luciferase/Renilla luciferase (LUC/REN) of the empty vector (EV) plus promoters under normal conditions were set as “1”. **E)** CUT&RUN qPCR assays tested TGA4 binding to *NRT1.1* and *NRT2.1* promoters under control and low nitrogen conditions. The relative enrichment of the WT *GFP-EV*-IgG (EV-IgG) under control conditions was set to “1”. WT, wild type. **F)** Biotin switch assay analyzed oxidative modification of MBP-TGA4. H_2_O_2_ concentrations: 0, 1, 10, 100 *μ*M, 1 mM **(G)** Effects of mutating Cys-334 on H_2_O_2_-induced cysteine oxidation in MBP-TGA4. MBP-TGA4 proteins treated with H_2_O_2_ (1 mM) were labeled with BIAM, captured with streptavidin beads, and detected by immunoblotting with anti-MBP antibody. **H)** Low nitrogen and H_2_O_2_ (500 *μ*M) treatments increased oxidative modification of TGA4. **I)** Oxidative modification of TGA4 was decreased in WT and *rboh1* mutants. Proteins in **(H, I)** were analyzed for biotin labeling. **J)** Dual-luciferase assays measured regulatory effects of TGA4 and TGA4^C334S^ on *NRT1.1* and *NRT2.1* promoters after Mock and H_2_O_2_ treatment. The ratios of LUC/REN for the EV combined with the promoters under normal conditions were set to “1”. **K)** CUT&RUN qPCR assessed TGA4 and TGA4^C334S^ binding to *NRT1.1* and *NRT2.1* promoters under control and low nitrogen conditions. The relative enrichment of the EV-IgG under control conditions was set to “1”. **L)** CUT&RUN qPCR evaluated TGA4 binding to *NRT1.1* and *NRT2.1* promoters in WT and *rboh1-1* mutants under control and low nitrogen conditions. The relative enrichment of WT *GFP-TGA4*-IgG (WT TGA4-IgG) samples under control conditions was set to “1”. Error bars represent SD; data are means ± SD of 3 biological replicates in **(A, F, G, H, I)**, 4 biological replicates in **(E, K, L)**, 6 biological replicates in **(D, J)**. Asterisks above the bars represent significant differences by Student's *t*-tests in **(D)**. ****, *P* < 0.0001. Experiments were repeated three times with similar results. Distinct letters above the bars signify significant differences at the *P* < 0.05 level, as determined by two-way ANOVA analysis **(J)** and three-way ANOVA **(E, K, L)** with Tukey's multiple comparison test. Precise *P*-values from these statistical tests are detailed in the Supplementary Data Set 10.

To explore how RBOH1-induced H_2_O_2_ influences *NRT1.1* and *NRT2.1* under low nitrogen conditions, we focused on whether H_2_O_2_ affects their expression via Oxi-PTMs of specific transcription factors. We identified five transcription factors, STOP1a, STOP1b, TGA1, TGA4, and TCP20, as potential regulators of *NRT1.1* and *NRT2.1* (Alvarez et al. 2014; Guan et al. 2014; Ye et al. 2021). Oxi-PTMs, particularly cysteine oxidative modifications, are thought to mediate these effects (Moller et al. 2007). To analyze Oxi-PTMs, we used a biotin switch assay to detect H_2_O_2_-sensitive cysteine residues and a BIAM labeling assay for H_2_O_2_-oxidized residues. Maltose-binding protein (MBP) fusions to each the transcription factors (MBP-STOP1a, MBP-STOP1b, MBP-TGA1, MBP-TGA4, MBP-TCP20) were pretreated with or without H_2_O_2_, then labeled with BIAM. SDS-PAGE and immunoblotting revealed that MBP-STOP1a and MBP-TGA4 BIAM labeling intensity decrease in a dose-dependent manner upon H_2_O_2_ treatment, indicating that H_2_O_2_ decreased the abundance of the reduced form of cysteine residues in these proteins (Fig. 8A and Supplementary Fig. S17). Although AtSTOP1 binds to the promoter of *AtNRT1.1* (Ye et al. 2021), its tomato homolog SlSTOP1a did not bind to the *SlNRT1.1* or *SlNRT2.1* promoters (Supplementary Fig. S18). TGA4, however, has a conserved “TGACG” binding site in the *NRT1.1* and *NRT2.1* promoters (Fig. 8B). Electrophoretic mobility shift assays (EMSAs) confirmed that TGA4 binds to these promoters in vitro (Fig. 8C). In vivo, luciferase (LUC) assays in *N. benthamiana* leaves with *pro35S:TGA4* as the effector and *proNRT1.1* or *proNRT2.1* as reporters showed that TGA4 significantly enhances LUC activity for both reporters (Fig. 8D). Additionally, cleavage under targets and release using nuclease (CUT&RUN) qPCR indicated that GFP-TGA4 binds more strongly to the *NRT1.1* and *NRT2.1* promoters under low nitrogen conditions compared to the GFP-EV control (Fig. 8E). Higher expression levels of *NRT1.1* and *NRT2.1* were observed in WT *GFP-TGA4* transgenic roots compared to WT *GFP-EV* under low nitrogen conditions (Supplementary Fig. S19). Immunoblot analysis showed that H_2_O_2_ induces TGA4 oxidation, with Cys-334 identified as a major oxidized residue (Fig. 8F and Supplementary Fig. S20). Mutation of Cys-334 significantly decreased the level of TGA4 oxidation after H_2_O_2_ treatment in vitro (Fig. 8G). Furthermore, in vivo assays demonstrated that nitrogen deprivation significantly induces TGA4 oxidation, which is further enhanced by H_2_O_2_, while mutation of Cys-334 led to significantly lower TGA4 oxidation under these conditions (Fig. 8H). *rboh1* mutants also showed lower TGA4 oxidation under low nitrogen stress (Fig. 8I).

To determine if H_2_O_2_-mediated Oxi-PTM of TGA4 promotes the expression of *NRT1.1* and *NRT2.1*, we performed a LUC activity assay in *N. benthamiana* leaves under control and H_2_O_2_-treated conditions. The expression of *pro35S*:*TGA4* significantly enhanced LUC activity from both *NRT1.1* and *NRT2.1* reporters, especially with H_2_O_2_ treatment (Fig. 8J). However, in leaves expressing *pro35S*:*TGA4^C334S^*, H_2_O_2_ treatment resulted in lower LUC activity compared to those expressing *pro35S*:*TGA4* (Fig. 8J), indicating that H_2_O_2_-mediated TGA4 Oxi-PTM at Cys-334 is crucial for TGA4 to enhance promoter binding of *NRT1.1* and *NRT2.1*. Further analysis using CUT&RUN qPCR revealed a significantly higher enrichment of *NRT1.1* and *NRT2.1* promoters in WT *GFP-TGA4* roots compared to WT *GFP-TGA4^C334S^* roots under low nitrogen conditions (Fig. 8K). Additionally, RT-qPCR showed that the expression levels of *NRT1.1* and *NRT2.1* are significantly lower in WT *GFP-TGA4^C334S^* roots compared to WT *GFP-TGA4* under low nitrogen stress (Supplementary Fig. S21). Moreover, the relative enrichment of *NRT1.1* and *NRT2.1* promoters was significantly higher in WT *GFP-TGA4* transgenic roots compared to *rboh1-1 GFP-TGA4* roots under low nitrogen stress (Fig. 8L). The mutation of *RBOH1* significantly diminished the ability of TGA4 to promote the expression of *NRT1.1* and *NRT2.1* under low nitrogen conditions (Supplementary Fig. S22). These results collectively indicate that Oxi-PTM of TGA4 by RBOH1-dependent H_2_O_2_ enhances the binding of TGA4 to the *NRT1.1* and *NRT2.1* promoters, thereby promoting their expression under low nitrogen stress.

## Discussion

SnRK1α1 is a crucial energy sensor involved in regulating plant metabolism, development, and stress responses by maintaining energy balance, particularly during nutrient starvation (Polge and Thomas 2007; Baena-González and Sheen 2008). SnRK1α1 phosphorylates NLP7 at serine 125 and serine 306, which inhibits NLP7-mediated regulation of primary nitrate responsive genes and affects nitrate-mediated plant growth in Arabidopsis under short photoperiods or low light intensity (Wang et al. 2022). Additionally, SnRK1α1 phosphorylates three conserved and functionally important serine residues, S29, S294, and S300, in bZIP63, altering its dimerization preference and influencing target gene expression and primary metabolism under extended night conditions (Mair et al. 2015). SnRK1α1 also regulates tolerance to nitrogen and carbon starvation in Arabidopsis by phosphorylating ATG1s and activating the autophagy pathway (Chen et al. 2017). Our study further reveals the positive influence of SnRK1α1 on nitrogen uptake and ROS production under low nitrogen stress in tomato (Fig. 1).

ROS levels in roots fluctuate with nutrient availability and play a crucial role in signal transduction (Shin et al. 2005). In Arabidopsis roots, H_2_O_2_ production serves as a signaling molecule under potassium-deficient conditions, where it positively regulates the expression of K^+^ transporter genes *K^+^ EFFLUX ANTIPORTER 5* (*KEA5*) and *HIGH AFFINITY K^+^ TRANSPORTER 5* (*HAK5*) (Shin and Schachtman 2004). Applying H_2_O_2_ induces the expression of these K^+^-responsive genes (Shin and Schachtman 2004). Similarly, nitrogen deprivation leads to H_2_O_2_ accumulation, acting as a versatile signaling modulator (Jung et al. 2018). Inhibiting H_2_O_2_ formation impedes the induction of nitrogen-starvation response genes, such as *NRT2.4* and *NRT2.5* (Safi et al. 2021). Our findings support these observations, demonstrating that ROS induce the expression of nitrate transporter genes (i.e. *NRT1.1* and *NRT2.1*) under low nitrate conditions (Fig. 3 and Supplementary Fig. S8). RBOH-induced ROS are essential for root development, mainly through RBOHF in Arabidopsis (Li et al. 2015). As the most homologous to Arabidopsis *RBOHF*, *SlRBOH1* is abundantly expressed in tomato roots (Fig. 2). We further investigated the role of RBOH1-induced ROS in tomato roots under low nitrogen stress. Treatment with H_2_O_2_ significantly enhanced tomato tolerance to low nitrogen stress, compensating for the nitrogen deficiency observed in *rboh1* mutants (Supplementary Fig. 10). This result confirms the crucial role of RBOH1-induced ROS in nitrate uptake in tomato roots.

Under nitrogen deprivation, increased ROS in root hair cells suggests a potential nitrogen-sensing mechanism (Shin et al. 2005). However, the precise mechanism by which plant roots detect nutrient stress and initiate ROS production remains unclear. We investigated how RBOH1 detects nitrate deficiency and stimulates ROS production in roots. Our findings revealed that *snrk1α1* exhibited a significant decrease in ROS production compared to WT under low nitrate stress (Fig. 1), a phenotype similar to that observed in *rboh1* (Fig. 2). Further analysis showed that SnRK1α1 regulates RBOH1-mediated ROS production by phosphorylating RBOH1 under low nitrogen stress (Figs. 4 and 5). Interestingly, it was reported that ROS also affect SnRK1α1 activity (Wurzinger et al. 2017). Thus, the upregulation of RBOH1-dependent ROS by SnRK1α1 and the downregulation of SnRK1α1 activity by ROS represent a dynamic strategy for managing plant stress responses. SnRK1α1-mediated phosphorylation of RBOH1 positively regulates ROS production, which in turn activates transcription factors such as TGA4 to induce the expression of genes related to nitrogen uptake and transport, including *NRT1.1* and *NRT2.1*, under low nitrogen stress. Concurrently, excessive ROS affects SnRK1α1 activity, creating a negative feedback loop that helps achieve a redox balance under low nitrogen stress. Protein phosphorylation is crucial for the regulation of RBOH activity. Plants respond to various stresses by phosphorylating different RBOHs or specific sites on RBOHs, thereby precisely controlling ROS production (Han et al. 2019). In Arabidopsis, RBOHF, which is homologous to tomato RBOH1, is a multifunctional NADPH oxidase involved in processes such as ROS-dependent ABA signaling, seed germination, root growth, and Na^+^/K^+^ balance under salinity stress (Kwak et al. 2003; Ma et al. 2012; Jiang et al. 2013). In Arabidopsis, CIPK26 mediates calcium responses by binding directly to RBOHF via its N terminus, particularly at residues S13, S130, and S132, leading to lower ROS production (Kimura et al. 2012; Han et al. 2019). ABA-activated SnRK2.6 can phosphorylate the N terminus of RBOHF at residues S13 and S174, positively regulating ABA signaling in Arabidopsis (Sirichandra et al. 2009). Residue S13 is notably important as both CIPK26 and SnRK2.6 can phosphorylate it. Similarly, our study identified SnRK1α1 as a kinase that specifically targets S188, S189, and S308 of RBOH1 in tomato, with S188 corresponding to S174 of RBOHF in Arabidopsis (Supplementary Fig. 2^3^). These findings suggest that S188 of RBOH1 is critical as it may be phosphorylated by both SnRK1α1 and SnRK2.6, integrating signals from nutrient stress and ABA. Further research is needed to understand how the same residue on RBOH1 differentiates kinase phosphorylation signals from various stresses to precisely regulate ROS production. Previous studies have not reported the phosphorylation of RBOH1 residues S189 and S308 by kinases to activate RBOH1/RBOHF, adding a dimension to the regulation of RBOH1 and the precise control of ROS production. Different stressors trigger specific signaling pathways that phosphorylate distinct residues of RBOHs through various kinases, potentially causing different ROS production patterns in response to specific stressors. This mechanism reflects the fine-tuned regulation and adaptability of plants to environmental stressors over evolutionary time. How these diverse regulatory mechanisms interact to modulate NADPH oxidase activity and ROS production remains unclear.

ROS production is essential for plant stress responses, as ROS can modulate protein function through Oxi-PTMs. For instance, ROS can affect the DNA-binding ability of various transcription factors, including CBF, bZIP68, HEAT SHOCK FACTOR A8 (HSFA8), and BZR1 (Giesguth et al. 2015; Tian et al. 2018; Li et al. 2019; Lee et al. 2021). Despite this, the role of ROS in regulating nitrate uptake remains poorly understood. Nitrate is the primary form of nitrogen taken up by plants, and nitrate uptake in roots is predominantly mediated by nitrate transporters located at the plasma membrane (Nacry et al. 2013; Krapp et al. 2014). The most well-documented nitrate transporters are NRT1.1 and NRT2.1 (Muños et al. 2004; Gutiérrez 2012). In tomato, *NRT1.1* and *NRT2.1* show higher expression levels compared to other nitrate transporter genes (Supplementary Fig. S8). The expression of these nitrate transporter genes is regulated by various transcription factors, such as TGA1, TGA4, TCP20, and STOP1 (Alvarez et al. 2014; Guan et al. 2014; Ye et al. 2021). Our study demonstrates that H_2_O_2_-induced oxidation of TGA4 is crucial for its binding to the promoters of *NRT1.1* and *NRT2.1*, identifying C334 as a key site for this interaction. This oxidative modification enhances the expression of *NRT1.1* and *NRT2.1*, thereby promoting nitrate uptake (Fig. 8). Although TGA1 and TGA4 are highly similar, TGA1 lacks the C334 residue present in TGA4 (Supplementary Fig. 2^4^), which may explain the decreased sensitivity of TGA1 to H_2_O_2_. Different transcription factors exhibit varying sensitivities to H_2_O_2_. We propose that H_2_O_2_, produced under stress conditions, modifies specific transcription factors through targeted oxidative changes. These modifications alter the structure of the transcription factors, affecting the expression of stress-responsive genes.

**Figure 9.**
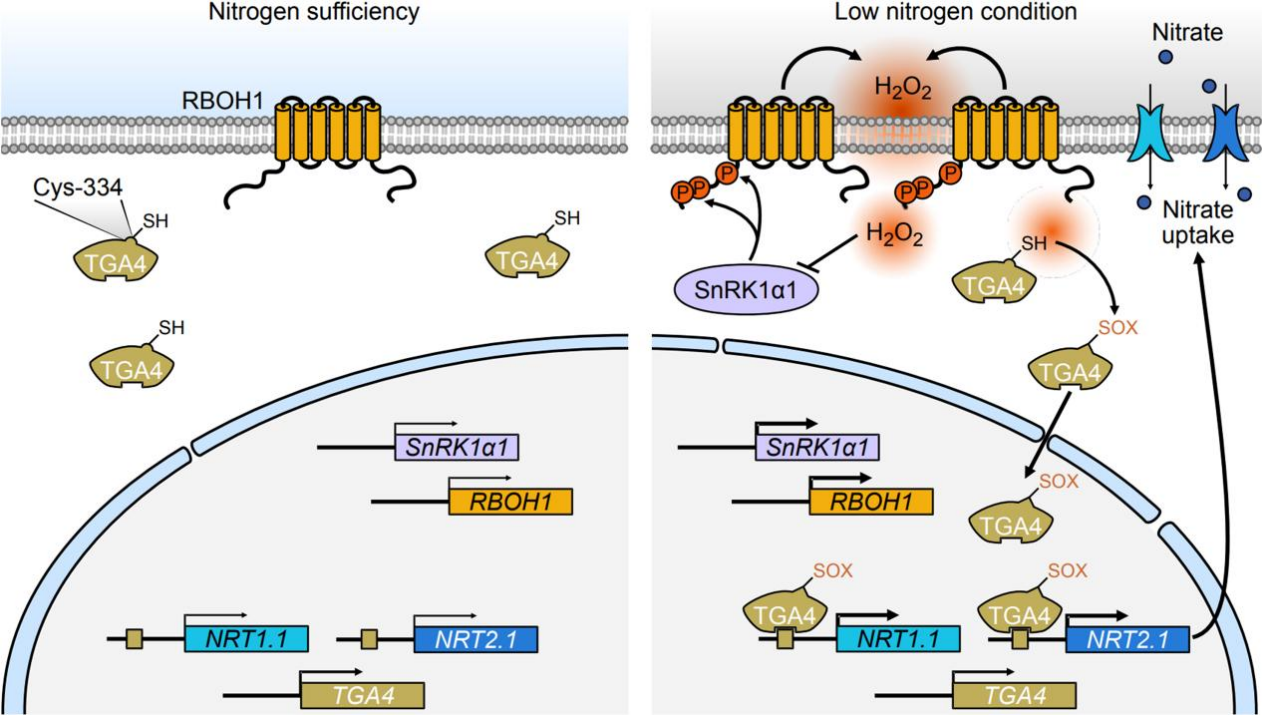
A model illustrating the role of SnRK1α1-RBOH1-induced H_2_O_2_ and its mediated oxidation of TGA4 in promoting low nitrogen tolerance. Under low nitrogen conditions, SnRK1α1 phosphorylates RBOH1 at Ser-188, Ser-189, and Ser-308, which activates H_2_O_2_ production. This H_2_O_2_ then induces oxidative modifications of TGA4, specifically at the Cys-334 residue. As a transcription factor, TGA4 binds to the promoters of *NRT1.1* and *NRT2.1*, thereby regulating their expression under low nitrogen conditions. The H_2_O_2_-mediated oxidation of TGA4 increases its binding affinity to these promoters, boosting the expression of *NRT1.1* and *NRT2.1* and promoting nitrate uptake in roots. Excessive H_2_O_2_ also impacts SnRK1α1 activity, creating a negative feedback loop to help maintain redox balance under low nitrogen stress. H_2_O_2_, hydrogen peroxide; Cys, cysteine; Ser, serine. The inhibitory arrow indicates that excessive H_2_O_2_ acts to repress the activity of SnRK1α1. The thin and thick arrows represent varying levels of transcriptional activity of the target genes.

Based on our study, we propose a working model where SnRK1α1–RBOH1-mediated ROS contributes to low-nitrogen tolerance (Fig. 9). In this model, the SnRK1α1–RBOH1 module regulates ROS production during nitrogen deprivation through specific phosphorylation sites. The interaction between SnRK1α1 and RBOH1, particularly through phosphorylation at S188, S189, and S308, enhances the role of SnRK1α1 in responding to nitrate deficiency. These phosphorylation sites are linked to NADPH enzyme activity, ROS production, and increased tolerance to low nitrogen stress. Under low nitrogen conditions, ROS production triggers a series of oxidative modifications in plant roots. Specifically, H_2_O_2_ promotes TGA4 binding to the promoters of *NRT1.1* and *NRT2.1* through Oxi-PTM. We identified C334 on TGA4 as a crucial site for this interaction. Our findings highlight the essential role of ROS in nitrate uptake and provide valuable insights into the mechanisms underlying nitrogen tolerance. This understanding offers potential pathways for molecular breeding and practical cultivation strategies to improve plant resilience to nitrogen stress.

## Materials and methods

### Plant materials, growth conditions, and low nitrogen treatment

All experiments used tomato seedlings (*Solanum lycopersicum* L. cv. Ailsa Craig) as the wild type (WT) background. Seedlings were grown under controlled conditions at 20 to 23 °C, with a 14-h light/10-h dark photoperiod and a light intensity of 600 *μ*mol m^−2^ s^−1^ in a growth room. For experiments involving nitrogen limitation, 3-week-old seedlings were transferred to either control or nitrogen-limited nutrient solutions and subjected to low nitrogen treatment for 3 weeks. The nutrient solutions were refreshed every other day to maintain consistent nutrient levels. Two different nutrient solutions were prepared to alter nitrate levels: (i) the control solution, which was based on a modified 1/2 Hoagland's nutrient formula and contained 10 mM and (ii) the low nitrogen solution, which had a significantly reduced nitrate concentration of 0.1 mM ${\mathrm{NO}}_{3}^{-}$. Both solutions included identical concentrations of other components: 3.75 mM CaCl_2_, 1.5 mM KH_2_PO_4_, 2.7 mM K_2_SO_4_, and 1 mM MgSO_4_·7H_2_O. The pH of ${\mathrm{NO}}_{3}^{-}$solutions was adjusted to 6.0 using NaOH. Trace elements were also added to all solutions according to our established experimental protocols (Cao et al. 2022).

### Phylogenetic analysis

Homologs of SnRK1 proteins were identified using the Sol genomics network (http://solgenomics.net/) for tomato and TAIR (www.arabidopsis.org) for Arabidopsis. A phylogenetic tree was reconstructed based on their amino acid sequences using the maximum-likelihood method in MEGA5.0 software. The alignment and tree files are provided as Supplementary Files 1 and 2.

### Constructs and plant transformation

Stable tomato lines with loss-of-function and gain-of-function mutations were developed using gene editing and overexpression techniques, respectively. The CRISPR/Cas9 constructs used for gene mutagenesis were created following established protocols (Cao et al. 2022; Guo et al. 2022; Zou et al. 2023). sgRNA sequences targeting *SnRK1α1* and *RBOH1* were custom-designed utilizing the CRISPR-P server (http://cbi.hzau.edu.cn/cgi-bin/CRISPR), with complete sgRNA details provided in Supplementary Table S1. Following synthesis and annealing, these sequences were inserted into the *Bbs*I site of the AtU6-sgRNA-AtUBQ-Cas9 vector. The resulting constructs were then integrated into the pCAMBIA1301 binary vector at the *HindIII* and *KpnI* sites. After transformation into *Agrobacterium tumefaciens* strain EHA105, the modified plasmids were used to infect cotyledons of Ailsa Craig tomato plants. Hygromycin-resistant transformed plants were sequenced at PCR-amplified target loci to confirm knockouts. Homozygous *snrk1α1* lines were isolated from the heterozygous Cas9-free F2 generation, and *rboh1* homozygous Cas9-free F2 lines were used for further studies (Supplementary Figs. S2, A and B and S5). For *SnRK1α1* overexpression (OE) lines, the full-length coding sequence (CDS) of *SnRK1α1* was inserted into the PFGC1008-HA vector downstream of the cauliflower mosaic virus (CaMV) *35S* promoter. Transgenic plants were generated through *A. tumefaciens*-mediated transformation with strain EHA105, and stable transgene expression was confirmed by immunoblotting using an anti-HA monoclonal antibody (Thermo Fisher Scientific, 26183) (Supplementary Fig. S2C). Homozygous T2 lines, verified independently, were selected for subsequent analysis. Primer details used in plasmid construction are listed in Supplementary Table S2.

To generate *RBOH1* transgenic roots driven by the *RBOH1* promoter, a 1980 bp DNA fragment upstream of the *RBOH1* translation start codons was PCR-amplified using specific primers and subcloned to replace the CaMV *35S* promoter in the pBI121-eGFP binary plasmid vector. Full-length CDSs of *RBOH1* and its point mutations (*RBOH1 ^S188A, S189A, S308A^* and *RBOH1 ^S188D, S189D, S308D^*) were amplified with specific primers and inserted into the vector (pBI121-eGFP) using a CloneExpress MultiS One-Step Cloning Kit (Vazyme, C113-01). Similarly, WT *GFP-TGA4,* WT *GFP-TGA4^C334S^*, and *rboh1-1 GFP-TGA4* transgenic roots were constructed, with the plasmids introduced into *A. rhizogenes* strain K599 for inoculation of the hypocotyls of 6-day-old sterile tomato seedlings grown on 1/2 strength Murashige & Skoog (MS) medium (Phyto Technology, M519). After 2 weeks of incubation on 1/2 MS medium, adventitious hairy roots emerged from the injured areas. Native roots were excised, and seedlings were transplanted into nutrient solutions, both control and nitrogen-deprived for further experimental treatments. A complete list of all primers utilized for the construction of the transgenic vectors can be found in Supplementary Table S2.

Virus-induced gene silencing (VIGS) using tobacco rattle virus (pTRV) was employed to silence the *RBOH1* gene in seedlings of WT and *SnRK1α1* OE lines. The pTRV2-*RBOH1* vector was used, and *A. tumefaciens* was used to mediate virus infection, following the method detailed in earlier studies (Zhou et al. 2014b). VIGS seedlings with approximately 20% to 30% of the transcript levels compared to control plants were used (Supplementary Fig. S11A).

### Protein extraction and immunoblotting

Protein extraction from root samples and western blotting were performed as described previously (Wang et al. 2019b). The root samples were ground in liquid nitrogen and homogenized using an extraction buffer composed of 20 mM HEPES (pH 7.5), 40 mM KCl, 1 mM EDTA, 1% Triton X-100, 10% glycerol, 1 mM phenylmethylsulfonyl fluoride, 5 mM DTT, and 25 mM sodium fluoride. For immunoblotting, proteins were separated by 10% SDS-PAGE gel and then transferred to nitrocellulose membranes. AMPK T172 protein was detected with anti-AMPK T172 monoclonal antibody (Cell Signaling Technology, 2535T). SnRK1α1 protein was detected with anti-SnRK1α1 polyclonal antibody (Biospring, BSR231211). ACC pS79 protein was detected with anti-ACC pS79 polyclonal antibody (Cell Signaling Technology, 3661S). The GFP-tagged ACC1 and the GFP-tagged TGA4 proteins were detected with anti-GFP monoclonal antibody (Thermo Fisher Scientific, MA5-15256). The HA-tagged SnRK1α1 protein was detected with anti-HA monoclonal antibody (Thermo Fisher Scientific, 26183). Actin protein was detected with anti-Actin polyclonal antibody (Abcam, ab197345).

### Determination of chlorophyll content

Chlorophyll content was determined using the method described previously (Wang et al. 2019b). Normal functional leaves were taken as samples for chlorophyll extraction. Leaf samples weighing 0.1 g were extracted using 10 mL of 80% acetone and kept in the dark until the leaves were completely decolorized. The samples were then centrifuged at 4,000 × *g* to obtain the supernatant, which was used for chlorophyll quantification. Chlorophyll content in the supernatant was determined using a spectrophotometer by measuring absorbance at wavelengths of 663 and 645 nm.

### Determination of nitrogen and carbon contents

The determination methods of carbon and nitrogen contents were described previously (Cao et al. 2022). The entire aerial parts and the entire roots were taken as samples for carbon and nitrogen content determination. These samples were then dried, finely ground, and passed through a 100-mesh sieve to ensure consistency. The nitrogen and carbon contents in the plant tissues were analyzed using a Flash IRMS Elemental Analyzer (Thermo Fisher Scientific, USA).

### Quantification, histochemical analysis, and cytochemical detection of ROS

The entire roots were taken as samples to determine of H_2_O_2_ content, and 0.1 g sample of tomato roots was utilized. The H_2_O_2_ levels were quantified using a Hydrogen Peroxide Assay Kit (Solarbio, BC3595), following the detailed instructions provided with the kit.

For 3,3′-diaminobenzidine (DAB) staining, root tips were first immersed in 50 mM Tris-HCl buffer (pH 3.8) for 15 min. They were then stained in 0.1% DAB solution under light for 10 min. After staining, the roots were rinsed three times in Tris-HCl buffer and decolorized in acid glycerol at 60 °C for 12 h. The processed root tips were examined with a Zeiss Stemi 305 stereomicroscope equipped with a Zeiss Axiocam ERc 5s camera (Zeiss, https://www.zeiss.com), maintaining consistent imaging parameters (Yan et al. 2020).

For H2DCF-DA (DCF) staining, root tips were soaked in 50 mM PBS (pH 7.4) for 30 min, followed by staining with H2DCF-DA (Sigma-Aldrich) in the dark for 10 min. After 3 washes with PBS, fluorescence images were captured using a Leica DM4000B microscope with a Leica DFC425C camera, using the same settings (exposure: 72.5 ms; gain: 2.1×; saturation: 0.85) as previously described (Yan et al. 2020).

For CeCl_3_ staining, root tips were immersed in 50 mM 3-(*N*-morpholino) propanesulfonic acid (MOPS) buffer (pH 7.2) and vacuum infiltrated with 5 mM CeCl_3_ solution in the dark. The roots were then fixed in 1.25% (v/v) glutaraldehyde and 1.25% (v/v) paraformaldehyde in 50 mM sodium cacodylate buffer (pH 7.2) for 1 h. Pretreatment for transmission electron microscopy was carried out as previously described (Zhou et al. 2014a), and samples were observed under a Hitachi H-7650 transmission electron microscope.

### Isolation of the plasma membrane and determination of NADPH oxidase activity

Isolation of the plasma membrane and measurement of NADPH oxidase activity were carried out as previously described (Zhou et al. 2012; Wang et al. 2019a). Plasma membranes were isolated from root samples using a modified two-phase aqueous polymer partition system. Initially, roots were homogenized in extraction buffer (50 mM Tris-HCl, pH 7.5, 0.25 M Suc, 1 mM AsA, 1 mM EDTA, 0.6% PVP, 1 mM PMSF) and filtered through cheesecloth. After centrifugation at 10,000 × *g* for 15 min, microsomal membranes were separated by further centrifugation at 50,000 × *g* for 30 min. The pellet was resuspended in a solution (0.33 M Suc, 3 mM KCl, 5 mM potassium phosphate, pH 7.8), and plasma membranes were isolated using a two-phase polymer system (6.2% Dextran T500, 6.2% polyethylene glycol 3350, 0.33 M Suc, 3 mM KCl, 5 mM potassium phosphate, pH 7.8) through three partitioning rounds. The upper phase was diluted in Tris-HCl buffer (10 mM, pH 7.4) containing 0.25 M sucrose, 1 mM EDTA, 1 mM DTT, 1 mM AsA, 1 mM PMSF, and centrifuged at 120,000 × *g* for 30 min. The final pellet was resuspended in Tris-HCl buffer for immediate further analysis. Protein content in the plasma membranes was determined using BSA as a standard. NADPH-dependent superoxide (O^2−^) generating activity was assessed by measuring the reduction of XTT by O^2−^. The assay mixture (1 mL) included 50 mM Tris-HCl buffer (pH 7.5), 0.5 mM XTT, 100 *μ*M NADPH, and 15 to 20 *μ*g of membrane proteins. The reaction was initiated by adding NADPH, and XTT reduction was monitored at 470 nm. Background activity was corrected by including 50 units of superoxide dismutase (SOD) in the reaction. Superoxide generation rates were calculated using an extinction coefficient of 2.16 × 10^4^ M^−1^ cm^−1^.

### In vivo SnRK1α1 activity assays

In vivo SnRK1α1 activity was assessed following the protocol as previously described (Sanagi et al. 2021). Tomato seedlings were treated for 3 days with either control or low nitrogen nutrient solutions, and root samples were collected. Proteins were extracted using 2× SDS sample buffer and denatured at 95 °C for 10 min. The extracts were centrifuged at 12,000 × *g* for 10 min at 25 °C to remove debris. Supernatants were analyzed via immunoblotting. SnRK1α1 activity was quantified by measuring the immunoblot signals with anti-AMPK pT172 antibody, normalized against signals from the SnRK1α1 endogenous antibody as controls (Supplementary Fig. S2B).

To assess SnRK1α1 activity using the ACC reporter, we constructed a plasmid containing tandemly repeated rat ACC1 peptides (ACC1 74-84, MRSSMSGLHLV) fused to GFP under the *35S* promoter. SnRK1α1 activity was evaluated in *snrk1α* mutants, WT, and *SnRK1α1* OE plants expressing the ACC reporter. Plants were grown on control media for 2 weeks and then were transferred to either control or low nitrogen conditions for 3 d. Proteins were extracted using the previously described method (Zou et al. 2023). SnRK1α1 activity was measured by quantifying immunoblot signals with anti-ACC pS79 antibody, normalized against signals from anti-GFP antibody, which served as controls for reporter expression.

### Y2H assays

The full-length CDSs of *SnRK1α1* and *SnRK1α2* were cloned into the pGADT7 vector, while the full-length CDSs of various *RBOH*s (*RBOHA-F*, *RBOH1*, and *RBOHH*) as well as the N-terminal (*RBOH1/N*) and C-terminal (*RBOH1/C*) fragments of *RBOH1* were inserted into the pGBKT7 vector. The primers utilized for these cloning processes are detailed in Supplementary Table S2. The pGADT7 and pGBKT7 constructs were co-transformed into yeast strain Y2H. Transformed yeast cells were grown on synthetic defined (SD)/-Trp-Leu (-LW) and SD/-Trp-Leu-His-Ade (-LWHA) solid media to assess protein interactions. The plates were incubated at 28 °C for 3 d, allowing for the evaluation of yeast growth and interaction between the expressed proteins.

### Pull-down assays

Pull-down assays were conducted with modifications based on previous methods (Zou et al. 2023). His-tagged proteins, including His-RBOH1/N and His-RBOH1/C fusion proteins, were purified using Ni-NTA agarose (Qiagen, 30230). GST and GST-SnRK1α1 fusion proteins were extracted with a buffer containing 10 mM NaH_2_PO_4_, 1.8 mM K_2_HPO_4_, 2.7 mM KCl, 140 mM NaCl, 1× protease inhibitor cocktail (Roche, 4693116001), 1% Triton X-100, and 1 mM phenylmethylsulfonyl fluoride. These proteins were then bound to glutathione agarose beads (Thermo Fisher Scientific, 16100). The beads with GST or GST-SnRK1α1 were incubated with equal amounts of His-tagged proteins at 4 °C for 1 h. After 6 washes, the beads were treated with SDS loading buffer, separated by SDS-PAGE, and analyzed by immunoblotting using anti-GST polyclonal antibody (Sigma-Aldrich, G7781) and anti-His polyclonal antibody (Cell Signaling Technology, 2365S).

### Split luciferase complementation assays

The CDSs of *SnRK1α1* and *RBOH1* were individually cloned into the pDONR207 vector. These genes were then recombined into the N/C-LUC vectors using the Gateway cloning system, with specific primers listed in Supplementary Table S2. After confirming the sequences, the vectors were transformed into *A. tumefaciens* strain GV3101. The transformed bacteria were used to infiltrate the leaves of *N. benthamiana*. Approximately 48 h post-infiltration, the dorsal surfaces of the leaves were treated with 0.5 mM luciferin. Following a 10-min incubation in the dark, the leaves were imaged using a low-temperature charge-coupled device imager (Song et al. 2023).

### Bimolecular fluorescence complementation (BiFC) assays

The CDSs of *RBOH1*, *RBOH1/N*, *RBOH1/C*, and *RBOHA*, or *SnRK1α1* and *SnRK1α2* were individually cloned into nYFP or cYFP vectors for BiFC analysis, using specific primers outlined in Supplementary Table S2. Appropriate controls were set based on the previous study (Kudla and Bock 2016). *Agrobacterium tumefaciens* strain GV3101, containing the various combinations of these constructs along with FLAGELLIN SENSITIVE 2 (FLS2)-mCherry (used as a plasma membrane localization marker), was utilized to infiltrate *N. benthamiana* leaves. Approximately 48 h post-infiltration, fluorescence was observed using a Nikon A1 confocal laser scanning microscope. The excitation and emission wavelengths for yellow fluorescent protein (YFP) detection were set at 514 nm and 520 to 560 nm, respectively, based on previously established parameters (Zou et al. 2023).

### Co-IP assays

The CDSs of *SnRK1α1* and *RBOH1/N* were cloned into vectors tagged with GFP and HA, respectively. These constructs were introduced into *A. tumefaciens* strain GV3101, which was then used to infiltrate *N. benthamiana* leaves. Two days post-infiltration, proteins were extracted using an IP buffer containing 10 mM HEPES pH 7.5, 100 mM NaCl, 1 mM EDTA, 10% glycerol, 1% Triton X-100, 1 mM PMSF, and 1 × protease inhibitor cocktail. The extracts were incubated with anti-GFP Magnetic Beads (Chromotek, gtma-20) for 2 h. The beads were subsequently washed 5 times with IP buffer to remove any unbound proteins. The immunoprecipitated proteins were subsequently analyzed by immunoblotting with an anti-HA monoclonal antibody (Thermo Fisher Scientific, 26183).

### Liquid chromatography tandem mass spectrometry (LC-MS/MS) analysis

The LC-MS/MS analysis for identifying phosphorylation and oxidation sites followed established protocols (Tian et al. 2018; Hu et al. 2021). To determine phosphorylation sites in RBOH1/N, a phosphorylation reaction was performed using His-RBOH1/N as the substrate and GST-SnRK1α1 as the kinase. The reaction was incubated at room temperature for 3 h. After incubation, the substrate proteins were separated by SDS-PAGE and subjected to trypsin digestion overnight. The resulting phosphopeptides were analyzed with an LTQ Orbitrap Elite mass spectrometer. For analyzing oxidized residues in TGA4, the purified maltose-binding protein-TGA4 fusion (MBP-TGA4) was labeled with biotin-IAM (BIAM). The biotinylated protein was then digested in-gel with trypsin and analyzed using LC-MS/MS on a ThermoFisher Orbitrap Fusion mass spectrometer.

### Phosphorylation assays

In vitro phosphorylation assays were carried out based on established protocols (Song et al. 2023). Recombinant GST-SnRK1α1 (2 *μ*g) was incubated with various His-RBOH1/N variants (4 *μ*g each) in a reaction buffer consisting of 25 mM Tris-HCl (pH 7.5), 10 mM MgCl_2_, 1 mM DTT, and 50 mM ATP at 28 °C for 1 h. Phosphorylation of the RBOH1/N proteins was detected using an anti-phospho-serine (anti-pSer) monoclonal antibody (Santa Cruz Biotechnology, sc-81514) following SDS-PAGE separation.

Additionally, in vivo phosphorylation assays were performed as described previously (Ding et al. 2023). Various *RBOH1/N-GFP* variants and *SnRK1α1-HA* constructs, expressed in *A. tumefaciens* strain GV3101, were used to infiltrate *N. benthamiana* leaves. Two days post-infiltration, proteins were extracted and incubated with anti-GFP Magnetic Beads (Chromotek, gtma-20). Phosphorylation was then detected with the anti-pSer monoclonal antibody (Santa Cruz Biotechnology, sc-81514) after SDS-PAGE separation.

### Total RNA extraction and gene expression analysis

Total RNA was extracted using the Total RNA Extraction Kit (Tiangen, DP419). RNA concentration and purity were assessed with a NanoDrop 2000 spectrophotometer (Thermo Fisher Scientific, USA). cDNA synthesis was performed using the Reverse Transcription Kit (Vazyme, R223). Quantitative PCR (qPCR) was conducted on the Light Cycler 480 II Real-Time PCR detection system (Roche, Germany) with ChamQ Universal SYBR qPCR Master Mix (Vazyme, Q711). Tomato *Actin* and *Ubi3* genes served as internal controls. Specific primers used for RT-qPCR are listed in Supplementary Table S3. Gene expression levels were quantified following the method described previously (Livak and Schmittgen 2001).

### RNA transcriptome analyses using RNA-seq

Root samples from WT and *rboh1-1* mutants, treated with low nitrogen for 3 d, were used for RNA-seq analysis. Each condition had three biological replicates to ensure data accuracy. RNA was isolated and processed as per the methodology described previously (Xie et al. 2022). RNA sequencing followed the manufacturer's protocol, with analysis performed by LC Biotech (http://www.lc-bio.com/). Poly(A) RNA was extracted using poly-T oligonucleotide-coated magnetic beads, fragmented to ∼300 bp, and reverse-transcribed to cDNA. Sequencing was conducted on an Illumina NovaseqTM 6000 platform by LC Sciences (Hangzhou, China), following recommended procedures. Transcript expression levels were estimated using StringTie v2.1.6 (http://ccb.jhu.edu/software/stringtie/) and ballgown (http://www.bioconductor.org/packages/release/bioc/html/ballgown.html). Differentially expressed genes were identified with a threshold of | log_2_(FPKM) | > 1 and a *P*-value <0.05.

### BIAM labeling assays

BIAM labeling assays were conducted with minor modifications to the previously described method (Tian et al. 2018). Proteins MBP-STOP1a, MBP-STOP1b, MBP-TGA1, MBP-TGA4, and MBP-TCP20 were purified from *E. coli* using amylose resin (NEB, E8021V) and exposed to varying concentrations of H_2_O_2_ at room temperature for 15 min. The proteins were then precipitated by adding acetone at −20 °C for 30 min, followed by centrifugation at 5,000 × *g* for 5 min. The pellets were washed three times with 50% acetone and resuspended in 500 *µ*l labeling buffer (50 mM MES-NaOH, pH 6.5, 100 mM NaCl, 1% Triton X-100, 100 *µ*M BIAM). The mixture was incubated in the dark at room temperature for 1 h. To halt the labeling reaction, β-mercaptoethanol was added to a final concentration of 20 mM. The mixtures were then precipitated at −20 °C for 30 min with acetone and centrifuged at 5,000 × *g* for 5 min. The resulting pellets were dissolved in 50 *µ*l of SDS sample buffer and analyzed by SDS-PAGE. BIAM-labeled proteins were detected using anti-biotin, HRP-linked antibody (Cell Signaling Technology, 7075S), while total MBP-STOP1a, MBP-STOP1b, MBP-TGA1, MBP-TGA4, and MBP-TCP20 proteins were visualized with anti-MBP monoclonal antibody (NEB, 8038L).

### In vivo and in vitro biotin switch assays

Biotin switch assays were adapted with minor modifications (Kim et al. 2000; Wang et al. 2012) to selectively biotinylate either reduced or oxidized thiol groups. These assays were employed to assess the redox status of TGA4 proteins in tomato plants as well as in purified recombinant TGA4 proteins treated with H_2_O_2_.

For in vivo oxidation analysis, *GFP-TGA4* and *GFP-TGA4^C334S^* transgenic seedlings were transplanted into nutrient solutions under both control and nitrogen-deprived conditions for further experimental treatments. Roots were then harvested, ground into a fine powder in liquid nitrogen, and proteins were extracted using EBR buffer (20 mM HEPES, pH 8.0, 40 mM KCl, 5 mM EDTA, 0.5% Triton X-100, 1% SDS, 1 mM PMSF, and 1× protease inhibitor cocktail).

For in vitro oxidation analysis, MBP-TGA4 proteins were purified from *E. coli* and then treated at room temperature with either 1 mM H_2_O_2_ or 1 mM DTT for 15 min. The proteins were precipitated using acetone at −20 °C for 30 min, followed by centrifugation at 5,000 × *g* for 5 min. The pellets were subjected to three washes with 50% acetone, followed by dissolution in 500 *µ*l of EBR buffer.

To identify the oxidized form of TGA4, plant extracts or recombinant TGA4 proteins were incubated with 100 mM N-ethylmaleimide (NEM) in EBR buffer at room temperature for 30 min, with periodic vortexing to block free thiol groups. The samples were then subjected to acetone precipitation, followed by three rounds of washing with 50% acetone. The resulting pellets were dissolved in 500 *µ*l of EBR buffer containing 20 mM DTT and incubated at 37 °C for 30 min. Following reduction, DTT was removed through protein precipitation, and the resulting pellet was re-dissolved in 500 *µ*l of EBR buffer. The supernatant was incubated with 100 *µ*M BIAM at room temperature in the dark for 1 h. To remove any excess BIAM, proteins were precipitated by adding acetone. The BIAM-labeled proteins were resuspended in 250 *µ*l of EBR buffer and then further diluted with 750 *µ*l of NEB buffer (20 mM HEPES, pH 8.0, 40 mM KCl, 5 mM EDTA, 0.25% Triton X-100, 1 mM PMSF, and 1× protease inhibitor cocktail). After centrifugation at 10,000 × *g* for 5 min, the supernatants were combined with 40 *µ*l of streptavidin beads (Biomag, VC298600) and incubated at 4 °C overnight. The beads were subjected to 5 washes with NEB buffer, and the bound proteins were then eluted using 50 *µ*l of 2 × SDS sample buffer. The samples were subsequently subjected to SDS-PAGE for analysis. Furthermore, a portion of the proteins, collected prior to incubation with streptavidin beads, was analyzed separately as a control for the total TGA4 protein levels. Immunoblotting was performed with anti-GFP monoclonal antibody (Thermo Fisher Scientific, MA5-15256) or anti-MBP monoclonal antibody (NEB, E8038L).

### Recombinant proteins and electrophoretic mobility shift assay (EMSA)

The MBP-TGA4 fusion proteins were affinity purified, and EMSA was conducted as previously described (Zou et al. 2023). For the EMSA, probes were biotinylated at the 3′ end using the Biotin 3′ End DNA Labeling Kit (Pierce, 89818) and annealed to form double-stranded probes. The binding assay was conducted according to the guidelines provided with the Light Shift Chemiluminescent EMSA Kit (Thermo Fisher Scientific, 20148). Biotin-EBNA Control DNA and EBNA extracts supplied with the kit were utilized as positive controls. The primers of probes used are listed in Supplementary Table S4.

### Dual-luciferase transcription activity assays

The dual-luciferase assays were performed with some modifications to previously described (Guo et al. 2024). Briefly, the full-length CDSs of *TGA4* and *TGA4^C334S^*, along with the promoters of *NRT1.1* and *NRT2.1*, were inserted into the pGreen II 0029 62-SK and pGreen II 0800-LUC vectors, respectively. Primers for vector construction are provided in Supplementary Table S2. The promoter PCR products were cloned into the pGreenII 0800-LUC vector to drive the expression of the firefly luciferase (LUC) reporter gene, while the *35S* promoter was used to drive the Renilla luciferase (REN) reporter gene as an internal control. Promoter activity was analyzed in *N. benthamiana* through transient expression mediated by *A. tumefaciens*. All constructs were introduced into *A. tumefaciens* strain GV3101. The resulting *A. tumefaciens* strains, containing the relevant constructs, were mixed and infiltrated into *N. benthamiana* leaves with an infiltration buffer. After 2 days of infiltration, LUC and REN activities were measured by the Dual Luciferase Reporter Assay Kit (Vazyme, DL101-01). The relative LUC/REN activity for combinations involving empty SK vectors and promoters was normalized to 1, and the analyses were conducted with six replicates.

### Cleavage under targets and release using nuclease (CUT&RUN) assays

Plant nuclei were extracted using the BestBio Plant Nucleus Extraction Kit (BestBio, BB-36112). CUT&RUN assays were conducted according to the manufacturer's instructions with the Hyperactive pG-M Nase CUT&RUN Assay Kit for PCR/qPCR (Vazyme, HD101) (Jia et al. 2024; Wang et al. 2024). Approximately 5 × 10^5^ nuclei were used for the reactions. Nuclei samples were incubated overnight at 4 °C with a goat anti-mouse immunoglobulin G (IgG) polyclonal antibody (EMD, Millipore AP124P) and anti-GFP monoclonal antibody (Thermo Fisher Scientific, MA5-15256), followed by cleavage and release steps. The DNA fragments from the CUT&RUN assay were purified using DNA purification buffers and spin columns. Finally, the DNA products were quantified by qPCR, with the primers used listed in Supplementary Table S5.

### Statistical analysis

Data are reported as the mean ± standard deviation (SD). Differences between two groups were evaluated using Student's *t*-tests. For comparisons involving multiple groups, one-way ANOVA, two-way ANOVA, and three-way ANOVA were utilized, followed by post hoc analysis using Tukey's test. Detailed descriptions of each statistical test are provided in the figure legends. Statistical data are provided in Supplementary Data Set 10.

### Accession numbers

The tomato genome sequence data utilized in this study were obtained from the tomato genome website (https://solgenomics.net/) using following the accession numbers: *SnRK1α1* (Solyc02g067030), *SnRK1α2* (Solyc03g115700), *RBOH1* (Solyc08g081690), *RBOHA* (Solyc01g099620), *RBOHB* (Solyc03g117980), *RBOHC* (Solyc05g025680), *RBOHD* (Solyc06g068680), *RBOHE* (Solyc06g075570), *RBOHF* (Solyc07g042460), *RBOHH* (Solyc11g072800), *STOP1a* (Solyc11g017140), *STOP1b* (Solyc06g065440), *TCP20* (Solyc02g068200), *TGA1* (Solyc04g011670), *TGA4* (Solyc04g054320), *NRT1.1* (Solyc08g078950), and *NRT2.1* (Solyc06g074990).

## Supplementary Material

koae321_Supplementary_Data

## Data Availability

Source data are provided with this article (Supplementary File 3). RNA-Seq data have been archived in the Sequence Read Archive (SRA) database at the National Center for Biotechnology Information (NCBI) under accession numbers PRJNA1075204 (SRR30233841 to SRR30233852).

## Supplementary data

The following materials are available in the online version of this article.

**Supplementary Figure S1.** A phylogenetic analysis of the SnRK1 family between Arabidopsis and tomato.

**Supplementary Figure S2.** Identification of *snrk1α1* mutants and *SnRK1α1* overexpression (*SnRK1α1* OE) plants.

**Supplementary Figure S3.** Phenotypes of tomato flowers, ovary, and styles from the flower bud to fruitlet stages in wild type (WT) and *snrk1α1* mutants.

**Supplementary Figure S4.** Nitrogen and carbon contents of shoots in wild type (WT), *snrk1α1*, and *SnRK1α1* overexpression (*SnRK1α1* OE) plants.

**Supplementary Figure S5.** Identification of *rboh1* mutants.

**Supplementary Figure S6.** Nitrogen and carbon contents of shoots in wild type (WT), *rboh1-1*, and *rboh1-2* mutants.

**Supplementary Figure S7.** RNA-seq analysis of wild type (WT) and *rboh1-1* roots under control and low-nitrogen conditions.

**Supplementary Figure S8.** Fragments Per Kilobase of transcript per Million mapped reads (FPKM) of nitrate transporters (*NRT*s) in RNA-seq between wild type (WT) and *rboh1-1*.

**Supplementary Figure S9.** Treatment with different H_2_O_2_ concentrations in wild type (WT).

**Supplementary Figure S10.** H_2_O_2_ treatment in different genotypes of tomato plants.

**Supplementary Figure S11.** Relative expression of *RBOH1* and nitrogen and carbon contents of roots in different genotypes of tomato plants.

**Supplementary Figure S12.** Yeast two-hybrid assays were conducted to test the interaction between SnRK1α1 and RBOHs.

**Supplementary Figure S13.** Yeast two-hybrid assays were conducted to test the interactions between SnRK1α1 and RBOHA, as well as SnRK1α2 and RBOH1 or RBOH1/N.

**Supplementary Figure S14.** Identification of SnRK1α1-targeted phosphorylation sites on RBOH1/N.

**Supplementary Figure S15.** Yeast two-hybrid assays were conducted to test the interaction between SnRK1α1 and RBOH1/N or its variants.

**Supplementary Figure S16.** GFP-tagged protein abundance and nitrogen and carbon contents of roots in different transgenic tomato plants.

**Supplementary Figure S17.** Analysis of oxidative modification of STOP1a, STOP1b, TGA1, and TCP20 by BIAM labeling assay.

**Supplementary Figure S18.** Regulatory effects of STOP1a on the promoters of *NRT1.1* and *NRT2.1* as determined by dual-luciferase assays.

**Supplementary Figure S19.** Relative expression of *NRT1.1* and *NRT2.1* in the WT *GFP-EV* and WT *GFP-TGA4* transgenic roots under control and low-nitrogen conditions.

**Supplementary Figure S20.** Mass spectrometry analysis of tryptic fragments of MBP-TGA4 protein treated as in Fig. 8F.

**Supplementary Figure S21.** Relative expression of *NRT1.1* and *NRT2.1* in the WT *GFP-EV* (EV), WT *GFP-TGA4* (TGA4) and WT *GFP-TGA4^C334S^* (TGA4^C334S^) transgenic roots under control and low-nitrogen conditions.

**Supplementary Figure S22.** Relative expression of *NRT1.1* and *NRT2.1* in the WT *GFP-TGA4* (WT TGA4) and *rboh1-1 GFP-TGA4* (*rboh1-1* TGA4) transgenic roots under control and low-nitrogen conditions.

**Supplementary Figure S23.** Comparison of amino acid sequences between tomato RBOH1 (SlRBOH1) and Arabidopsis RBOHF (AtRBOHF).

**Supplementary Figure S24.** Comparison of amino acid sequences between TGA1 and TGA4 in tomato.

**Supplementary Table S1.** Target sequences used for CRISPR-Cas9-mediated gene editing.

**Supplementary Table S2.** Primers used for plasmid construction.

**Supplementary Table S3.** Primers used for RT-qPCR.

**Supplementary Table S4.** Primers used for EMSA.

**Supplementary Table S5.** Primers used for CUT&RUN.

**Supplementary Data Set 1.** Differentially expressed genes (DEGs) of WT between low-nitrogen (LN) stress and control conditions.

**Supplementary Data Set 2.** Differentially expressed genes (DEGs) of *rboh1-1* mutants between low-nitrogen (LN) stress and control conditions.

**Supplementary Data Set 3.** Differentially expressed genes (DEGs) of WT and *rboh1-1* mutants under low-nitrogen (LN) stress.

**Supplementary Data Set 4.** Gene ontology (GO) annotation and categorization of genes downregulated in *rboh1-1* mutants compared to WT under low-nitrogen stress.

**Supplementary Data Set 5.** Fragments per kilobase of transcript per million mapped reads (FPKM) of seven nitrate transporters (*NRTs*) in RNA-seq between wild type (WT) and *rboh1-1*.

**Supplementary Data Set 6.** Kyoto Encyclopedia of Genes and Genomes (KEGG) enrichment analysis of genes downregulated in *rboh1-1* mutants compared to WT under low-nitrogen stress.

**Supplementary Data Set 7.** Differentially expressed genes (DEGs) of *rboh1-1* mutants and WT under control conditions.

**Supplementary Data Set 8.** Gene ontology (GO) annotation and categorization of the differentially expressed genes (DEGs) between *rboh1-1* mutants and WT under control conditions.

**Supplementary Data Set 9.** Kyoto Encyclopedia of Genes and Genomes (KEGG) enrichment analysis of the differentially expressed genes (DEGs) between *rboh1-1* mutants and WT under control conditions.

**Supplementary Data Set 10.** Statistical data.

**Supplementary File 1.** Alignment used for phylogenetic analysis.

**Supplementary File 2.** Machine-readable phylogenetic tree.

## Dive Curated Terms

The following phenotypic, genotypic, and functional terms are of significance to the work described in this paper:

BZR1 Gramene: AT1G75080
BZR1 Araport: AT1G75080

OST1 Gramene: AT4G33950
OST1 Araport: AT4G33950

ARF6 Gramene: AT1G30330
ARF6 Araport: AT1G30330

HAK5 Gramene: AT4G13420
HAK5 Araport: AT4G13420

KEA5 Gramene: AT5G51710
KEA5 Araport: AT5G51710

NLP7 Gramene: AT4G24020
NLP7 Araport: AT4G24020

BIK1 Gramene: AT2G39660
BIK1 Araport: AT2G39660

ARF19 Gramene: AT1G19220
ARF19 Araport: AT1G19220

EDTA Gramene: Ethylene Diamine Tetraacetic Acid
EDTA Araport: Ethylene Diamine Tetraacetic Acid

YFP Gramene: yellow fluorescent protein
YFP Araport: yellow fluorescent protein

nitrate CHEBI: CHEBI:17632

ATP CHEBI: CHEBI:15422

RBOH1 Gramene: Solyc08g081690
RBOH1 Araport: Solyc08g081690

EMSA Gramene: electrophoretic mobility shift assay
EMSA Araport: electrophoretic mobility shift assay

NRT1.1 Gramene: Solyc08g078950
NRT1.1 Araport: Solyc08g078950

NRT2.1 Gramene: Solyc06g074990
NRT2.1 Araport: Solyc06g074990

SnRK1a1 Gramene: Solyc02g067030
SnRK1a1 Araport: Solyc02g067030

SnRK1a2 Gramene: Solyc03g115700
SnRK1a2 Araport: Solyc03g115700

RBOHA Gramene: Solyc01g099620
RBOHA Araport: Solyc01g099620

RBOHB Gramene: Solyc03g117980
RBOHB Araport: Solyc03g117980

RBOHC Gramene: Solyc05g025680
RBOHC Araport: Solyc05g025680

RBOHD Gramene: Solyc06g068680
RBOHD Araport: Solyc06g068680

RBOHE Gramene: Solyc06g075570
RBOHE Araport: Solyc06g075570

RBOHF Gramene: Solyc07g042460
RBOHF Araport: Solyc07g042460

RBOHH Gramene: Solyc11g072800
RBOHH Araport: Solyc11g072800

STOP1a Gramene: Solyc11g017140
STOP1a Araport: Solyc11g017140

STOP1b Gramene: Solyc06g065440
STOP1b Araport: Solyc06g065440

TCP20 Gramene: Solyc02g068200
TCP20 Araport: Solyc02g068200

TGA1 Gramene: Solyc04g011670
TGA1 Araport: Solyc04g011670

TGA4 Gramene: Solyc04g054320
TGA4 Araport: Solyc04g054320

## References

[koae321-B1] Alvarez JM , Riveras E, Vidal EA, Gras DE, Contreras-López O, Tamayo KP, Aceituno F, Gómez I, Ruffel S, Lejay L, et al Systems approach identifies TGA1 and TGA4 transcription factors as important regulatory components of the nitrate response of *Arabidopsis thaliana* roots. Plant J. 2014:80(1):1–13. 10.1111/tpj.12618 25039575

[koae321-B2] Baena-González E , Sheen J. Convergent energy and stress signaling. Trends Plant Sci. 2008:13(9):474–482. 10.1016/j.tplants.2008.06.006 18701338 PMC3075853

[koae321-B3] Belda-Palazón B , Costa M, Beeckman T, Rolland F, Baena-González E. ABA represses TOR and root meristem activity through nuclear exit of the SnRK1 kinase. Proc Natl Acad Sci U S A. 2022:119(28):e2215090119. 10.1073/pnas.2204862119 35787039 PMC9282376

[koae321-B4] Bi GZ , Hu M, Fu L, Zhang XJ, Zuo JR, Li JY, Yang J, Zhou J-M. The cytosolic thiol peroxidase PRXIIB is an intracellular sensor for H_2_O_2_ that regulates plant immunity through a redox relay. Nat Plants. 2022:8(10):1160–1175. 10.1038/s41477-022-01252-5 36241731

[koae321-B5] Cao J , Zheng X, Xie D, Zhou H, Shao S, Zhou J. Autophagic pathway contributes to low-nitrogen tolerance by optimizing nitrogen uptake and utilization in tomato. Hortic Res. 2022:9:uhac068. 10.1093/hr/uhac068 35669705 PMC9164271

[koae321-B6] Chapman JM , Muhlemann JK, Gayornba SR, Muday GK. RBOH-dependent ROS synthesis and ROS scavenging by plant specialized metabolites to modulate plant development and stress responses. Chem Res Toxicol. 2019:32(3):370–396. 10.1021/acs.chemrestox.9b00028 30781949 PMC6857786

[koae321-B7] Chen L , Su Z-Z, Huang L, Xia F-N, Qi H, Xie L-J, Xiao S, Chen Q-F. The AMP-activated protein kinase KIN10 is involved in the regulation of autophagy in Arabidopsis. Front Plant Sci. 2017:8:1201. 10.3389/fpls.2017.01201 28740502 PMC5502289

[koae321-B8] Ding ST , Lv JR, Hu ZJ, Wang J, Wang P, Yu JQ, Foyer CH, Shi K. Phytosulfokine peptide optimizes plant growth and defense via glutamine synthetase GS2 phosphorylation in tomato. EMBO J. 2023:42(6):e111858. 10.15252/embj.2022111858 36562188 PMC10015362

[koae321-B9] Emanuelle S , Hossain MI, Moller IE, Pedersen HL, van de Meene AML, Doblin MS, Koay A, Oakhill JS, Scott JW, Willats WGT, et al SnRK1 from *Arabidopsis thaliana* is an atypical AMPK. Plant J. 2015:82(2):183–192. 10.1111/tpj.12813 25736509

[koae321-B10] Fu LW , Wang PC, Xiong Y. Target of rapamycin signaling in plant stress responses. Plant Physiol. 2020:182(4):1613–1623. 10.1104/pp.19.01214 31949028 PMC7140942

[koae321-B11] Giesguth M , Sahm A, Simon S, Dietz K-J. Redox-dependent translocation of the heat shock transcription factor AtHSFA8 from the cytosol to the nucleus in *Arabidopsis thaliana*. FEBS Lett. 2015:589(6):718–725. 10.1016/j.febslet.2015.01.039 25666709

[koae321-B12] Gilroy S , Suzuki N, Miller G, Choi W-G, Toyota M, Devireddy AR, Mittler R. A tidal wave of signals: calcium and ROS at the forefront of rapid systemic signaling. Trends Plant Sci. 2014:19(10):623–630. 10.1016/j.tplants.2014.06.013 25088679

[koae321-B13] Guan PZ , Wang RC, Nacry P, Breton G, Kay SA, Pruneda-Paz JL, Davani A, Crawford NM. Nitrate foraging by Arabidopsis roots is mediated by the transcription factor TCP20 through the systemic signaling pathway. Proc Natl Acad Sci U S A. 2014:111(42):15267–15272. 10.1073/pnas.1411375111 25288754 PMC4210337

[koae321-B14] Guo M , Yang F, Liu C, Zou J, Qi Z, Fotopoulos V, Lu G, Yu J, Zhou J. A single-nucleotide polymorphism in WRKY33 promoter is associated with the cold sensitivity in cultivated tomato. New Phytol. 2022:236(3):989–1005. 10.1111/nph.18403 35892173

[koae321-B15] Guo M , Yang F, Zhu L, Wang L, Li Z, Qi Z, Fotopoulos V, Yu J, Zhou J. Loss of cold tolerance is conferred by absence of the WRKY34 promoter fragment during tomato evolution. Nat Commun. 2024:15(1):6667–6667. 10.1038/s41467-024-51036-y 39107290 PMC11303406

[koae321-B16] Gutiérrez RA . Systems biology for enhanced plant nitrogen nutrition. Science. 2012:336(6089):1673–1675. 10.1126/science.1217620 22745422

[koae321-B17] Han JP , Köster P, Drerup MM, Scholz M, Li SZ, Edel KH, Hashimoto K, Kuchitsu K, Hippler M, Kudla J. Fine-tuning of RBOHF activity is achieved by differential phosphorylation and Ca2+ binding. New Phytol. 2019:221(4):1935–1949. 10.1111/nph.15543 30320882

[koae321-B18] Hermans C , Hammond JP, White PJ, Verbruggen N. How do plants respond to nutrient shortage by biomass allocation? Trends Plant Sci. 2006:11(12):610–617. 10.1016/j.tplants.2006.10.007 17092760

[koae321-B19] Ho C-H , Lin S-H, Hu H-C, Tsay Y-F. CHL1 functions as a nitrate sensor in plants. Cell. 2009:138(6):1184–1194. 10.1016/j.cell.2009.07.004 19766570

[koae321-B20] Hu Z , Li J, Ding S, Cheng F, Li X, Jiang Y, Yu J, Foyer CH, Shi K. The protein kinase CPK28 phosphorylates ascorbate peroxidase and enhances thermotolerance in tomato. Plant Physiol. 2021:186(2):1302–1317. 10.1093/plphys/kiab120 33711164 PMC8195530

[koae321-B21] Jia D , Wang Q, Qi Y, Jiang Y, He J, Lin Y, Sun Y, Xu J, Chen W, Fan L, et al Microbial metabolite enhances immunotherapy efficacy by modulating T cell stemness in pan-cancer. Cell. 2024:187(7):1651–1665. 10.1016/j.cell.2024.02.022 38490195

[koae321-B22] Jiang CF , Belfield EJ, Mithani A, Visscher A, Ragoussis J, Mott R, Smith JAC, Harberd NP. ROS-mediated vascular homeostatic control of root-to-shoot soil Na delivery in Arabidopsis. EMBO J. 2013:32(6):914–914. 10.1038/emboj.2013.43 PMC3501220 23064146

[koae321-B23] Jung J-Y , Ahn JH, Schachtman DP. CC-type glutaredoxins mediate plant response and signaling under nitrate starvation in Arabidopsis. BMC Plant Biol. 2018:18(1):281. 10.1186/s12870-018-1512-1 30424734 PMC6234535

[koae321-B24] Kadota Y , Sklenar J, Derbyshire P, Stransfeld L, Asai S, Ntoukakis V, Jones JD, Shirasu K, Menke F, Jones A, et al Direct regulation of the NADPH oxidase RBOHD by the PRR-associated kinase BIK1 during plant immunity. Mol Cell. 2014:54(1):43–55. 10.1016/j.molcel.2014.02.021 24630626

[koae321-B25] Kiba T , Krapp A. Plant nitrogen acquisition under low availability: regulation of uptake and root architecture. Plant Cell Physiol. 2016:57(4):707–714. 10.1093/pcp/pcw052 27025887 PMC4836452

[koae321-B26] Kim JW , Yoon HS, Kwon KY, Lee SR, Rhee SG. Identification of proteins containing cysteine residues that are sensitive to oxidation by hydrogen peroxide at neutral pH. Anal Biochem. 2000:283(2):214–221. 10.1006/abio.2000.4623 10906242

[koae321-B27] Kimura S , Kaya H, Kawarazaki T, Hiraoka G, Senzaki E, Michikawa M, Kuchitsu K. Protein phosphorylation is a prerequisite for the Ca2+-dependent activation of Arabidopsis NADPH oxidases and may function as a trigger for the positive feedback regulation of Ca2+ and reactive oxygen species. Biochim Biophys Acta. 2012:1823(2):398–405. 10.1016/j.bbamcr.2011.09.011 22001402

[koae321-B28] Kotur Z , Mackenzie N, Ramesh S, Tyerman SD, Kaiser BN, Glass ADM. Nitrate transport capacity of the *Arabidopsis thaliana* NRT2 family members and their interactions with AtNAR2.1. New Phytol. 2012:194(3):724–731. 10.1111/j.1469-8137.2012.04094.x 22432443

[koae321-B29] Krapp A , David LC, Chardin C, Girin T, Marmagne A, Leprince AS, Chaillou S, Ferrario-Mery S, Meyer C, Daniel-Vedele F. Nitrate transport and signalling in Arabidopsis. J Exp Bot. 2014:65(3):789–798. 10.1093/jxb/eru001 24532451

[koae321-B30] Kudla J , Bock R. Lighting the way to protein-protein interactions: recommendations on best practices for bimolecular fluorescence complementation analyses. Plant Cell. 2016:28(5):1002–1008. 10.1105/tpc.16.00043 27099259 PMC4904677

[koae321-B31] Kwak JM , Mori IC, Pei Z-M, Leonhardt N, Torres MA, Dangl JL, Bloom RE, Bodde S, Jones JDG, Schroeder JI. NADPH oxidase AtrbohD and AtrbohF genes function in ROS-dependent ABA signaling in Arabidopsis. EMBO J. 2003:22(11):2623–2633. 10.1093/emboj/cdg277 12773379 PMC156772

[koae321-B32] Lee ES , Park JH, Wi SD, Kang CH, Chi YH, Chae HB, Paeng SK, Ji MG, Kim W-Y, Kim MG, et al Redox-dependent structural switch and CBF activation confer freezing tolerance in plants. Nat Plants. 2021:7(7):914–922. 10.1038/s41477-021-00944-8 34155371

[koae321-B33] Lezhneva L , Kiba T, Feria-Bourrellier AB, Lafouge F, Boutet-Mercey S, Zoufan P, Sakakibara H, Daniel-Vedele F, Krapp A. The Arabidopsis nitrate transporter NRT2.5 plays a role in nitrate acquisition and remobilization in nitrogen-starved plants. Plant J. 2014:80(2):230–241. 10.1111/tpj.12626 25065551

[koae321-B34] Li L , Li M, Yu LP, Zhou ZY, Liang XX, Liu ZX, Cai GH, Gao LY, Zhang XJ, Wang YC, et al The FLS2-associated kinase BIK1 directly phosphorylates the NADPH oxidase RbohD to control plant immunity. Cell Host Microbe. 2014:15(3):329–338. 10.1016/j.chom.2014.02.009 24629339

[koae321-B35] Li N , Sun LR, Zhang LY, Song YL, Hu PP, Li C, Hao FS. Atrbohd and AtrbohF negatively regulate lateral root development by changing the localized accumulation of superoxide in primary roots of Arabidopsis. Planta. 2015:241(3):591–602. 10.1007/s00425-014-2204-1 25399352

[koae321-B36] Li YM , Liu WZ, Zhong H, Zhang H-L, Xia YJ. Redox-sensitive bZIP68 plays a role in balancing stress tolerance with growth in Arabidopsis. Plant J. 2019:100(4):768–783. 10.1111/tpj.14476 31348568

[koae321-B37] Liu D , Li Y-Y, Zhou Z-C, Xiang XH, Liu X, Wang J, Hu Z-R, Xiang S-P, Li W, Xiao Q-Z, et al Tobacco transcription factor bHLH123 improves salt tolerance by activating NADPH oxidase NtRbohE expression. Plant Physiol. 2021a:186(3):1706–1720. 10.1093/plphys/kiab176 33871656 PMC8260122

[koae321-B38] Liu YL , Duan XL, Zhao XD, Ding WL, Wang YW, Xiong Y. Diverse nitrogen signals activate convergent ROP2-TOR signaling in Arabidopsis. Dev Cell. 2021b:56(9):1283–1295.e5. 10.1016/j.devcel.2021.03.022 33831352

[koae321-B39] Livak KJ , Schmittgen TD. Analysis of relative gene expression data using real-time quantitative PCR and the 2-ΔΔCT method. Methods. 2001:25(4):402–408. 10.1006/meth.2001.1262 11846609

[koae321-B40] Ludewlg U , Neuhduser B, Dynowski M. Molecular mechanisms of ammonium transport and accumulation in plants. FEBS Lett. 2007:581(12):2301–2308. 10.1016/j.febslet.2007.03.034 17397837

[koae321-B41] Ma LY , Zhang H, Sun LR, Jiao YH, Zhang GZ, Miao C, Hao FS. NADPH oxidase AtrbohD and AtrbohF function in ROS-dependent regulation of Na+/K+ homeostasis in Arabidopsis under salt stress. J Exp Bot. 2012:63(1):305–317. 10.1093/jxb/err280 21984648

[koae321-B42] Mair A , Pedrotti L, Wurzinger B, Anrather D, Simeunovic A, Weiste C, Valerio C, Dietrich K, Kirchler T, Nägele T, et al SnRK1-triggered switch of bZIP63 dimerization mediates the low-energy response in plants. Elife. 2015:4:05828. 10.7554/eLife.05828 PMC4558565 26263501

[koae321-B43] Mittler R , Zandalinas SI, Fichman Y, Van Breusegem F. Reactive oxygen species signalling in plant stress responses. Nat Rev Mol Cell Biol. 2022:23(10):663–679. 10.1038/s41580-022-00499-2 35760900

[koae321-B44] Moller IM , Jensen PE, Hansson A. Oxidative modifications to cellular components in plants. Annu Rev Plant Biol. 2007:58:459–481. 10.1146/annurev.arplant.58.032806.103946 17288534

[koae321-B45] Muños S , Cazettes C, Fizames C, Gaymard F, Tillard P, Lepetit M, Lejay L, Gojon A. Transcript profiling in the chl1-5mutant of Arabidopsis reveals a role of the nitrate transporter NRT1.1 in the regulation of another nitrate transporter, NRT2.1. Plant Cell. 2004:16(9):2433–2447. 10.1105/tpc.104.024380 15319483 PMC520944

[koae321-B46] Muralidhara P , Weiste C, Collani S, Krischke M, Kreisz P, Draken J, Feil R, Mair A, Teige M, Müller MJ, et al Perturbations in plant energy homeostasis prime lateral root initiation via SnRK1-bZIP63-ARF19 signaling. Proc Natl Acad Sci U S A. 2021:118(37):e2106961118. 10.1073/pnas.2106961118 34504003 PMC8449399

[koae321-B47] Nacry P , Bouguyon E, Gojon A. Nitrogen acquisition by roots: physiological and developmental mechanisms ensuring plant adaptation to a fluctuating resource. Plant Soil. 2013:370(1–2):1–29. 10.1007/s11104-013-1645-9

[koae321-B48] Nietzel T , Mostertz J, Ruberti C, Née G, Fuchs P, Wagner S, Moseler A, Müller-Schüssele SJ, Benamar A, Poschet G, et al Redox-mediated kick-start of mitochondrial energy metabolism drives resource-efficient seed germination. Proc Natl Acad Sci U S A. 2020:117(1):741–751. 10.1073/pnas.1910501117 31871212 PMC6955286

[koae321-B49] Polge C , Thomas M. SNF1/AMPK/SnRK1 kinases, global regulators at the heart of energy control? Trends Plant Sci. 2007:12(1):20–28. 10.1016/j.tplants.2006.11.005 17166759

[koae321-B50] Rodriguez M , Parola R, Andreola S, Pereyra C, Martínez-Noël G. TOR and SnRK1 signaling pathways in plant response to abiotic stresses: do they always act according to the “yin-yang” model? Plant Sci. 2019:288:110220. 10.1016/j.plantsci.2019.110220 31521220

[koae321-B51] Safi A , Medici A, Szponarski W, Martin F, Clément-Vidal A, Marshall-Colon A, Ruffel S, Gaymard F, Rouached H, Leclercq J, et al GARP transcription factors repress Arabidopsis nitrogen starvation response via ROS-dependent and -independent pathways. J Exp Bot. 2021:72(10):3881–3901. 10.1093/jxb/erab114 33758916 PMC8096604

[koae321-B52] Sagi M , Fluhr R. Production of reactive oxygen species by plant NADPH oxidases. Plant Physiol. 2006:141(2):336–340. 10.1104/pp.106.078089 16760484 PMC1475462

[koae321-B53] Sanagi M , Aoyama S, Kubo A, Lu Y, Sato Y, Ito S, Abe M, Mitsuda N, Ohme-Takagi M, Kiba T, et al Low nitrogen conditions accelerate flowering by modulating the phosphorylation state of FLOWERING BHLH 4 in Arabidopsis. Proc Natl Acad Sci U S A. 2021:118(19):e2022942118. 10.1073/pnas.2022942118 33963081 PMC8126780

[koae321-B54] Shin R , Berg RH, Schachtman DP. Reactive oxygen species and root hairs in Arabidopsis root response to nitrogen, phosphorus and potassium deficiency. Plant Cell Physiol. 2005:46(8):1350–1357. 10.1093/pcp/pci145 15946982

[koae321-B55] Shin R , Schachtman DP. Hydrogen peroxide mediates plant root cell response to nutrient deprivation. Proc Natl Acad Sci U S A. 2004:101(23):8827–8832. 10.1073/pnas.0401707101 15173595 PMC423280

[koae321-B56] Sies H , Jones DP. Reactive oxygen species (ROS) as pleiotropic physiological signalling agents. Nat Rev Mol Cell Biol. 2020:21(7):363–383. 10.1038/s41580-020-0230-3 32231263

[koae321-B57] Sirichandra C , Gu D, Hu HC, Davanture M, Lee S, Djaoui M, Valot B, Zivy M, Leung J, Merlot S, et al Phosphorylation of the Arabidopsis AtrbohF NADPH oxidase by OST1 protein kinase. FEBS Lett. 2009:583(18):2982–2986. 10.1016/j.febslet.2009.08.033 19716822

[koae321-B58] Song J , Lin R, Tang M, Wang L, Fan P, Xia X, Yu J, Zhou Y. SlMPK1- and SlMPK2-mediated SlBBX17 phosphorylation positively regulates CBF-dependent cold tolerance in tomato. New Phytol. 2023:239(5):1887–1902. 10.1111/nph.19072 37322592

[koae321-B59] Tian Y , Fan M, Qin Z, Lv H, Wang M, Zhang Z, Zhou W, Zhao N, Li X, Han C, et al Hydrogen peroxide positively regulates brassinosteroid signaling through oxidation of the BRASSINAZOLE-RESISTANT1 transcription factor. Nat Commun. 2018:9(1):1063. 10.1038/s41467-018-03463-x 29540799 PMC5852159

[koae321-B60] Wang G , Hu C, Zhou J, Liu Y, Cai J, Pan C, Wang Y, Wu X, Shi K, Xia X, et al Systemic root-shoot signaling drives jasmonate-based root defense against nematodes. Curr Biol. 2019a:29(20):3430–3438. 10.1016/j.cub.2019.08.049 31588001

[koae321-B61] Wang H , Han C, Wang JG, Chu X, Shi W, Yao L, Chen J, Hao W, Deng Z, Fan M, et al Regulatory functions of cellular energy sensor SnRK1 for nitrate signalling through NLP7 repression. Nat Plants. 2022:8(9):1094–1107. 10.1038/s41477-022-01236-5 36050463

[koae321-B62] Wang H , Wang S, Lu Y, Avarez S, Hicks LM, Ge X, Xia Y. Proteomic analysis of early-responsive redox-sensitive proteins in arabidopsis. J Proteome Res. 2012:11(1):412–424. 10.1021/pr200918f 22050424 PMC3253204

[koae321-B63] Wang R , He F, Ning Y, Wang G-L. Fine-tuning of RBOH-mediated ROS signaling in plant immunity. Trends Plant Sci. 2020a:25(11):1060–1062. 10.1016/j.tplants.2020.08.001 32861572

[koae321-B64] Wang W , Li JY, Cui SY, Li JY, Ye XL, Wang Z, Zhang TT, Jiang X, Kong YL, Chen X, et al Microglial Ffar4 deficiency promotes cognitive impairment in the context of metabolic syndrome. Sci Adv. 2024:10(5):eadj7813. 10.1126/sciadv.adj7813 38306420 PMC10836723

[koae321-B65] Wang Y , Cao J-J, Wang K-X, Xia X-J, Shi K, Zhou Y-H, Yu J-Q, Zhou J. BZR1 mediates brassinosteroid-induced autophagy and nitrogen starvation in tomato. Plant Physiol. 2019b:179(2):671–685. 10.1104/pp.18.01028 30482787 PMC6426427

[koae321-B66] Wang Y , Wang LP, Micallef BJ, Tetlow IJ, Mullen RT, Feil R, Lunn JE, Emes MJ. AKINβ1, a subunit of SnRK1, regulates organic acid metabolism and acts as a global modulator of genes involved in carbon, lipid, and nitrogen metabolism. J Exp Bot. 2020b:71(3):1010–1028. 10.1093/jxb/erz460 31624846

[koae321-B67] Wurzinger B , Mair A, Fischer-Schrader K, Nukarinen E, Roustan V, Weckwerth W, Teige M. Redox state-dependent modulation of plant SnRK1 kinase activity differs from AMPK regulation in animals. FEBS Lett. 2017:591(21):3625–3636. 10.1002/1873-3468.12852 28940407 PMC5698759

[koae321-B68] Xie D-L , Huang H-M, Zhou C-Y, Liu C-X, Kanwar MK, Qi Z-Y, Zhou J. Hsfa1a confers pollen thermotolerance through upregulating antioxidant capacity, protein repair, and degradation in *Solanum lycopersicum* L. Hortic Res. 2022:9:uhac163. 10.1093/hr/uhac163 36204210 PMC9531336

[koae321-B69] Xiong Y , McCormack M, Li L, Hall Q, Xiang CB, Sheen J. Glucose-TOR signalling reprograms the transcriptome and activates meristems. Nature. 2013:496(7444):181–186. 10.1038/nature12030 23542588 PMC4140196

[koae321-B70] Yan M-Y , Xie D-L, Cao J-J, Xia X-J, Shi K, Zhou Y-H, Zhou J, Foyer CH, Yu J-Q. Brassinosteroid-mediated reactive oxygen species are essential for tapetum degradation and pollen fertility in tomato. Plant J. 2020:102(5):931–947. 10.1111/tpj.14672 31908046

[koae321-B71] Ye JY , Tian WH, Zhou M, Zhu QY, Du WX, Zhu YX, Liu XX, Lin XY, Zheng SJ, Jin CW. STOP1 activates NRT1.1-mediated nitrate uptake to create a favorable rhizospheric pH for plant adaptation to acidity. Plant Cell. 2021:33(12):3658–3674. 10.1093/plcell/koab226 34524462 PMC8643680

[koae321-B72] Zhang W , Zhi W, Qiao H, Huang J, Li S, Lu Q, Wang N, Li Q, Zhou Q, Sun J, et al H_2_O_2_-dependent oxidation of the transcription factor GmNTL1 promotes salt tolerance in soybean. Plant Cell. 2024:36(1):112–135. 10.1093/plcell/koad250 PMC10734621 37770034

[koae321-B73] Zhou H , Huang JJ, Willems P, Van Breusegem F, Xie YJ. Cysteine thiol-based post-translational modification: what do we know about transcription factors? Trends Plant Sci. 2023:28(4):415–428. 10.1016/j.tplants.2022.11.007 36494303

[koae321-B74] Zhou J , Wang J, Li X, Xia X-J, Zhou Y-H, Shi K, Chen Z, Yu J-Q. H_2_O_2_ mediates the crosstalk of brassinosteroid and abscisic acid in tomato responses to heat and oxidative stresses. J Exp Bot. 2014a:65(15):4371–4383. 10.1093/jxb/eru217 24899077 PMC4112640

[koae321-B75] Zhou J , Wang J, Shi K, Xia XJ, Zhou YH, Yu JQ. Hydrogen peroxide is involved in the cold acclimation-induced chilling tolerance of tomato plants. Plant Physiol Biochem. 2012:60:141–149. 10.1016/j.plaphy.2012.07.010 22935478

[koae321-B76] Zhou J , Xia X-J, Zhou Y-H, Shi K, Chen Z, Yu J-Q. RBOH1-dependent H_2_O_2_ production and subsequent activation of MPK1/2 play an important role in acclimation-induced cross-tolerance in tomato. J Exp Bot. 2014b:65(2):595–607. 10.1093/jxb/ert404 24323505 PMC3904713

[koae321-B77] Zou J , Chen X, Liu C, Guo M, Kanwar MK, Qi Z, Yang P, Wang G, Bao Y, Bassham DC, et al Autophagy promotes jasmonate-mediated defense against nematodes. Nat Commun. 2023:14(1):4769. 10.1038/s41467-023-40472-x 37553319 PMC10409745

[koae321-B78] Zou J-J , Li X-D, Ratnasekera D, Wang C, Liu W-X, Song L-F, Zhang W-Z, Wu W-H. Arabidopsis CALCIUM-DEPENDENT PROTEIN KINASE8 and CATALASE3 function in abscisic acid-mediated signaling and H_2_O_2_ homeostasis in stomatal guard cells under drought stress. Plant Cell. 2015:27(5):1445–1460. 10.1105/tpc.15.00144 25966761 PMC4456645

