# Supplementary material for: SnRK1α1-mediated RBOH1 phosphorylation regulates reactive oxygen species to enhance tolerance to low nitrogen in tomato
Source: Plant Cell. 2024 Dec 12;37(1):koae321. doi: 10.1093/plcell/koae321 (PMC11684077; doi:10.1093/plcell/koae321)
Supplement: koae321_Supplementary_Data [file koae321_supplementary_data.zip › Supplementary Tables.pdf]

SnRK1α1-mediated RBOH1 phosphorylation regulates reactive oxygen species to enhance tolerance to low nitrogen in tomato

Xuelian Zheng, Hongfei Yang, Jinping Zou, Weiduo Jin, Zhenyu Qi, Ping Yang, Jingquan Yu, Jie Zhou*

Supplementary Table S1. Target sequences used for CRISPR-Cas9 -mediated gene editing

Name of sgRNA	Sequence
SnRK1α1-sgRNA	TAAAAATGAGTCAACACTGC
RBOH1-sgRNA	ACGTCGGATACGGTGTCTTC

Supplementary Table S2. Primers used for plasmid constructions

Name	Sequence
SnRK1α1-HA-F	ttacaattaccatggggcgcgccATGGACGGAACAGCAGTGCA
SnRK1α1-HA-R	aacatcgtatgggtaggtaccAAGTACTCGAAGCTGAGCAAGAAA
TRV2-RBOH1-F	gtgagtaaggttaccATGAGGGGTTTACCTGGGCA
TRV2-RBOH1-R	cgtgagctcggtaccCGGCGATCGAGTTTCCGA
pGBKT7-SnRK1α1-F	atggccatggaggccgaattcATGGACGGAACAGCAGTGCA
pGBKT7-SnRK1α1-R	ccgctgcaggtcgacggatccAAGTACTCGAAGCTGAGCAAGAAA
pGBKT7-SnRK1α2-F	atggccatggaggccgaattcATGAGTTCCAGAGGTGGTGGAA
pGBKT7-SnRK1α2-R	ccgctgcaggtcgacggatccTTGTGGCCCCTCTAGCTGC
pGADT7-RBOH1-F	gccatggaggccagtgaattcATGAGGGGTTTACCTGGGCA
pGADT7-RBOH1-R	cagctcgagctcgatggatccAAAATGTTCTTTGTGAAACTCGAACT
pGADT7-RBOH1/N-F	gccatggaggccagtgaattcATGAGGGGTTTACCTGGGCA
pGADT7-RBOH1/N-R	cagctcgagctcgatggatccTCTCTTCCAATTCTCTTGCAGTGA
pGADT7-RBOH1/C-F	gccatggaggccagtgaattcATTGGAAGAGAATTTGGGTTCTGG
pGADT7-RBOH1/C-R	cagctcgagctcgatggatccAAAATGTTCTTTGTGAAACTCGAACT
pGADT7-RBOHA-F	gccatggaggccagtgaattcATGGAGATCGAAAACACGACAGA
pGADT7-RBOHA-R	cagctcgagctcgatggatccGAAATTTTCTTTATGAAATTCAAACTTTG
pGADT7-RBOHB-F	gccatggaggccagtgaattcATGCAAAATTCGGAAAATCATCA
pGADT7-RBOHB-R	cagctcgagctcgatggatccAAAATTTTCTTTATGGAAATCAAACTTG
pGADT7-RBOHC-F	gccatggaggccagtgaattcATGCAGTTAATGTCACTTTTTAGGTCA
pGADT7-RBOHC-R	cagctcgagctcgatggatccTACTTGAGATTGTAGAAATTCTTTACCTACTG
pGADT7-RBOHD-F	gccatggaggccagtgaattcATGCAAAATCCAGAAGATCACCA
pGADT7-RBOHD-R	cagctcgagctcgatggatccAAAGTTTTCTTTATGGAAATCAAACTTT
pGADT7-RBOHE-F	gccatggaggccagtgaattcATGGTGCCCATTGACGATGG
pGADT7-RBOHE-R	cagctcgagctcgatggatccGAAATTTTCTTTGTGGAAATTGAAAC
pGADT7-RBOHF-F	gccatggaggccagtgaattcATGTCGGAGGAGCAATGTTACC
pGADT7-RBOHF-R	cagctcgagctcgatggatccAAAGTACTCTTTGTGGAACTCAAATCG
pGADT7-RBOHH-F	gccatggaggccagtgaattcATGGCAAGGAGAAAGAAGATTAATG
pGADT7-RBOHH-R	cagctcgagctcgatggatccGAAGTTCTCTTTGTGGAAGTTGAAGC
pGADT7-RBOH1/N^{S188A}-F	ACTCGTgcaAGCGCACACAAGGCTCTTCGTGG
pGADT7-RBOH1/N^{S188A}-R	TGTGCGCTtgcACGAGTCCGGTCGAGCTGAGC
pGADT7-RBOH1/N^{S189A}-F	TCGTTCCgcaGCACACAAGGCTCTTCGTGGAC
pGADT7-RBOH1/N^{S189A}-R	TGTGTGCtgcGGAACGAGTCCGGTCGAGCTGA
pGADT7-RBOH1/N^{S308A}-F	TCATGCTAgcaGCCTCTGCAAACAAATTATCAAGA
pGADT7-RBOH1/N^{S308A}-R	AGAGGCtgcTAGCATGATGATCTCTTTTACTTCTTCTT
pGADT7-RBOH1/N$^{S188A,\ S189A}$-F	TCGTgcagcaGCACACAAGGCTCTTCGTGGACT
pGADT7-RBOH1/N$^{S188A,\ S189A}$-R	TTGTGTGCtgctgcACGAGTCCGGTCGAGCTGA
GST-SnRK1α1-F	gatctggttccgcgtggatccATGGACGGAACAGCAGTGCA
GST-SnRK1α1-R	ctcgagtcgacccgggaattcAAGTACTCGAAGCTGAGCAAGAAA
His-RBOH1/N-F	gccatggctgatatcggatccATGAGGGGTTTACCTGGGCA
His-RBOH1/N-R	gcaagcttgtcgacggagctcTCTCTTCCAATTCTCTTGCAGTGA
His-RBOH1/C-F	gccatggctgatatcggatccATGATTGGAAGAGAATTTGGGTTC
His-RBOH1/C-R	gcaagcttgtcgacggagctcAAAATGTTCTTTGTGAAACTCGAACT

Supplementary Table S2 continued

His-RBOH1/N^{S188A}-F	ACTCGTgcaAGCGCACACAAGGCTCTTCGTGG
His-RBOH1/N^{S188A}-R	TGTGCGCTtgcACGAGTCCGGTCGAGCTGAGC
His-RBOH1/N^{S189A}-F	TCGTTCCgcaGCACACAAGGCTCTTCGTGGAC
His-RBOH1/N^{S189A}-R	TGTGTGCtgcGGAACGAGTCCGGTCGAGCTGA
His-RBOH1/N^{S308A}-F	TCATGCTAgcaGCCTCTGCAAACAAATTATCAAGA
His-RBOH1/N^{S308A}-R	AGAGGCtgcTAGCATGATGATCTCTTTTACTTCTTCTT
His-RBOH1/N$^{S188A, S189A}$-F	TCGTgcagcaGCACACAAGGCTCTTCGTGGACT
His-RBOH1/N$^{S188A, S189A}$-R	TTGTGTGCtgctgcACGAGTCCGGTCGAGCTGA
SnRK1α1-cLUC-F	ggggacaagtttgtacaaaaaagcaggctttATGGACGGAACAGCAGTGCA
SnRK1α1-cLUC-R	ggggaccactttgtacaagaaagctgggtcAAGTACTCGAAGCTGAGCAA
RBOH1/N-nLUC-F	ggggacaagtttgtacaaaaaagcaggctttATGAGGGGTTTACCTGGGCA
RBOH1/N-nLUC-R	ggggaccactttgtacaagaaagctgggtcTCTCTTCCAATTCTCTTGCA
cYFP-SnRK1α1-F	atttacgaacgatagttaattaacATGGACGGAACAGCAGTGCA
cYFP-SnRK1α1-R	actgccacctcctccactagtAAGTACTCGAAGCTGAGCAAGAAA
RBOH1-nYFP-F	atttacgaacgatagttaattaacATGAGGGGTTTACCTGGGCA
RBOH1-nYFP-R	actgccacctcctccactagtAAAATGTTCTTTGTGAAACTCGAACT
RBOH1/N-nYFP-F	atttacgaacgatagttaattaacATGAGGGGTTTACCTGGGCA
RBOH1/N-nYFP-R	actgccacctcctccactagtTCTCTTCCAATTCTCTTGCAGTGA
RBOH1/C-nYFP-F	atttacgaacgatagttaattaacATGATTGGAAGAGAATTTGGGTTC
RBOH1/C-nYFP-R	actgccacctcctccactagtAAAATGTTCTTTGTGAAACTCGAACT
RBOHA-nYFP-F	atttacgaacgatagttaattaacATGGAGATCGAAAACACGACAGA
RBOHA-nYFP-R	actgccacctcctccactagtGAAATTTTCTTTATGAAATTCAAACTTTG
cYFP-SnRK1α2-F	atttacgaacgatagttaattaacATGAGTTCCAGAGGTGGTGGAA
cYFP-SnRK1α2-R	actgccacctcctccactagtTTGTGGCCCCTCTAGCTGC
RBOH1/N-GFP-F	ctctcgagctttcgcgagctcATGAGGGGTTTACCTGGGCA
RBOH1/N-GFP-R	gcccttgctcaccatggatccTCTCTTCCAATTCTCTTGCAGTGA
RBOH1/N^{S188A}-GFP-F	ACTCGTgcaAGCGCACACAAGGCTCTTCGTGG
RBOH1/N^{S188A}-GFP-R	TGTGCGCTtgcACGAGTCCGGTCGAGCTGAGC
RBOH1/N^{S189A}-GFP-F	TCGTTCCgcaGCACACAAGGCTCTTCGTGGAC
RBOH1/N^{S189A}-GFP-R	TGTGTGCtgcGGAACGAGTCCGGTCGAGCTGA
RBOH1/N^{S308A}-GFP-F	TCATGCTAgcaGCCTCTGCAAACAAATTATCAAGA
RBOH1/N^{S308A}-GFP-R	AGAGGCtgcTAGCATGATGATCTCTTTTACTTCTTCTT
RBOH1/N$^{S188A, S189A}$-GFP-F	TCGTgcagcaGCACACAAGGCTCTTCGTGGACT
RBOH1/N$^{S188A, S189A}$-GFP-R	TTGTGTGCtgctgcACGAGTCCGGTCGAGCTGA
proRBOH1:GFP -F	gaccatgattacgccaagcttGGCCATAACAATATTCATACATGTCG
proRBOH1:GFP -R	gcccttgctcaccatggtaccCTGTCCCAAATCAAATGACAATGA
proRBOH1:GFP-RBOH1-F	gacgagctgtacaagctcgagATGAGGGGTTTACCTGGGCA
proRBOH1:GFP-RBOH1-R	cgatcggggaaattcgagctcAAAATGTTCTTTGTGAAACTCGAACT
proRBOH1:GFP-RBOH1$^{S188A, S189A}$-F	TCGTgcagcaGCACACAAGGCTCTTCGTGGACT
proRBOH1:GFP-RBOH1$^{S188A, S189A}$-R	TTGTGTGCtgctgcACGAGTCCGGTCGAGCTGA
proRBOH1:GFP-RBOH1^{S308A}-F	TCATGCTAgcaGCCTCTGCAAACAAATTATCAAGA
proRBOH1:GFP-RBOH1^{S308A}-R	AGAGGCtgcTAGCATGATGATCTCTTTTACTTCTTCTT
proRBOH1:GFP-RBOH1$^{S188D, S189D}$-F	TCGTgacgacGCACACAAGGCTCTTCGTGGACT

Supplementary Table S2 continued

proRBOH1:GFP-RBOH1$^{S188D, S189D}$-R	TTGTGTGCgtcgtcACGAGTCCGGTCGAGCTGA
proRBOH1:GFP-RBOH1^{S308D}-F	TCATGCTAgacGCCTCTGCAAACAAATTATCAAGA
proRBOH1:GFP-RBOH1^{S308D}-R	AGAGGCgtcTAGCATGATGATCTCTTTTACTTCTTCTT
MBP-TGA1-F	gagggaaggatttcagaattcATGAATTCTTCAACATACACTCAGTTTG
MBP-TGA1-R	caggtcgactctagaggatccGGCCGGCTCATGAAGACG
MBP-TGA4-F	gagggaaggatttcagaattcATGAATTCTTCAACATATACTCAATTTGTT
MBP-TGA4-R	caggtcgactctagaggatccAGCAGGTTCAGAAAGACGTCCA
MBP-TGA4^{C334S}-F	GATGTCCAGCATACTGGCTCCGCACCAATCAG
MBP-TGA4^{C334S}-R	CCAGTATGCTGGACATCTGTAGAAGGGTTTCTTGG
MBP-TCP20-F	gagggaaggatttcagaattcATGGATCCCAAACAGGCTAACC
MBP-TCP20-R	caggtcgactctagaggatccATGTCCTGATCCTTGAGAATCCTC
MBP-STOP1a-F	gagggaaggatttcagaattcATGCCCTCAGATAACCATTCATTT
MBP-STOP1a-R	caggtcgactctagaggatccTTCCATTTCACTAGAACTCATAAATTTTG
MBP-STOP1b-F	gagggaaggatttcagaattcATGGAACAACAGGCTAATCAAAGC
MBP-STOP1b-R	caggtcgactctagaggatccTATTTCCAGCCTTTGTAGTTGCAT
ACC1-GFP-F	ctctcgagctttcgcgagctcATGAGATCCAGCATGTCTGGCT
ACC1-GFP-R	gcccttgctcaccatggatccTACTAGGTGCAAGCCAGACATGC
TGA4-GFP-F	ctctcgagctttcgcgagctcATGAATTCTTCAACATATACTCAATTTGTT
TGA4-GFP-R	gcccttgctcaccatggatccAGCAGGTTCAGAAAGACGTCCA
TGA4^{C334S}-GFP-F	GATGTCCAGCATACTGGCTCCGCACCAATCAG
TGA4^{C334S}-GFP-R	CCAGTATGCTGGACATCTGTAGAAGGGTTTCTTGG
SK-STOP1a-F	cgctctagaactagtggatccATGCCCTCAGATAACCATTCATTT
SK-STOP1a-R	tgatttcagcgaattggtaccTTCCATTTCACTAGAACTCATAAATTTTG
SK-TGA4-F	cgctctagaactagtggatccATGAATTCTTCAACATATACTCAATTTGTT
SK-TGA4-R	tgatttcagcgaattggtaccAGCAGGTTCAGAAAGACGTCCA
SK-TGA4^{C334S}-F	GATGTCCAGCATACTGGCTCCGCACCAATCAG
SK-TGA4^{C334S}-R	CCAGTATGCTGGACATCTGTAGAAGGGTTTCTTGG
proNRT1.1-LUC-F	gtcgacggtatcgataagcttTAAATAAAAATCTCTTATTAATATATTATTTTAAATCG
proNRT1.1-LUC-R	cgctctagaactagtggatccAAGTTGTATAGTTCCCGGGTATCTTG
proNRT2.1-LUC-F	gtcgacggtatcgataagcttAAGTTGACTTGGTTTCATTACATCAGA
proNRT2.1-LUC-R	cgctctagaactagtggatccCCAACCAATTATGGAAGCCTGA
proNRT1.1$^{-2000bp}$-LUC-F	gtcgacggtatcgataagcttGCCTAACAGTCTTTTATTAATAACAAAATAA
proNRT1.1$^{-2000bp}$-LUC-R	cgctctagaactagtggatccAAGATTCAATTGATTCTATAACCCTTTT
proNRT2.1$^{-2000bp}$-LUC-F	gtcgacggtatcgataagcttCCCTTTTAACTCCGCACTCCT
proNRT2.1$^{-2000bp}$-LUC-R	cgctctagaactagtggatccCCAACCAATTATGGAAGCCTGA

Supplementary Table S3. Primers used for RT-qPCR

Name	Sequence
SnRK1α1-F	TTATCAGCTGGTGCGAGGGA
SnRK1α1-R	GTGGAGGCACGGCCAAATAG
NRT1.1-F	CACATCGGAAAATTCGAGATCC
NRT1.1-R	GCCGCAATCATAGCTATAATCG
NRT2.1-F	CAAAGAATTGAAGGATCACCGG
NRT2.1-R	AGTGAAAAAGGAGATCCAGGAG
RBOH1-F	TCCAGCACAAGATTACCG
RBOH1-R	CCTCCATTGCGACGAT
RBOHA-F	CGAGAGTAGGATTCAGCGGT
RBOHA-R	TGCAGCTTCATGATCAGCAC
RBOHB-F	CAACATGAAGGCCATGGACGAA
RBOHB-R	CTGTGCCATATTTGGGCTAGCATT
RBOHC-F	TGAGCCACAGTACGCCTTTA
RBOHC-R	TAGCAAGCAACCACAGCAAG
RBOHD-F	AAGCCAACCGCATCCTCCTC
RBOHD-R	GCAGTAAGCTCGTCGAATCGC
RBOHE-F	CGGATGGAATCAGGCGCTAC
RBOHE-R	TCTCTGAAGATCCTCCATTAACTGC
RBOHF-F	ATCTGCACGAGAGGAAATGAAATCG
RBOHF-R	CAAACCGCGCCTCCACTTT
RBOHH-F	CCACGGCTGCTTCATATTCC
RBOHH-R	CGTGGTAGCGGTTCTCATTG
Ubiquitin3-F	GCCGACTACAACATCCAGAAGG
Ubiquitin3-R	TGCAACACAGCGAGCTTAACC
Actin-F	TGGTCGGAATGGGACAGAAG
Actin-R	CTCAGTCAGGAGAACAGGGT

Supplementary Table S4. Primers used for EMSA

Name	Sequence
NRT1.1-probe-F	TATCTTTTTGACGTGCAATA
NRT1.1-probe-R	TATTGCACGTCAAAAAGATA
NRT2.1-probe-F	AAACACACGTCAGGCTTCCA
NRT2.1-probe-R	TGGAAGCCTGACGTGTGTTT
Mu-NRT1.1-probe-F	TATCTTTTAAAAATGCAATA
Mu-NRT1.1-probe-R	TATTGCATTTTTAAAAGATA
Mu-NRT2.1-probe-F	AAACACAAAAAAGGCTTCCA
Mu-NRT2.1-probe-R	TGGAAGCCTTTTTTGTGTTT

Supplementary Table S5. Primers used for CUT&RUN

Name	Sequence
NRT1.1-CUT&RUN-F	CCCTATTGTTTGGGTGCATTCT
NRT1.1-CUT&RUN-R	GTCTGGCCCAGGAATTTTACAAC
NRT2.1-CUT&RUN-F	TTTGCAATGCCCTTATTCTTTG
NRT2.1-CUT&RUN-R	TCTTCACGTTTGCCTCACCC
