# Supplementary material for: SnRK1α1-mediated RBOH1 phosphorylation regulates reactive oxygen species to enhance tolerance to low nitrogen in tomato
Source: Plant Cell. 2024 Dec 12;37(1):koae321. doi: 10.1093/plcell/koae321 (PMC11684077; doi:10.1093/plcell/koae321)
Supplement: koae321_Supplementary_Data [file koae321_supplementary_data.zip › TPC2024RA11290DR1_Supplementary Figures and Tables.pdf]

SnRK1α1-mediated RBOH1 phosphorylation regulates reactive oxygen species to enhance tolerance to low nitrogen in tomato

Xuelian Zheng, Hongfei Yang, Jinping Zou, Weiduo Jin, Zhenyu Qi, Ping Yang, Jingquan Yu, Jie Zhou*

Supplementary Fig. S1

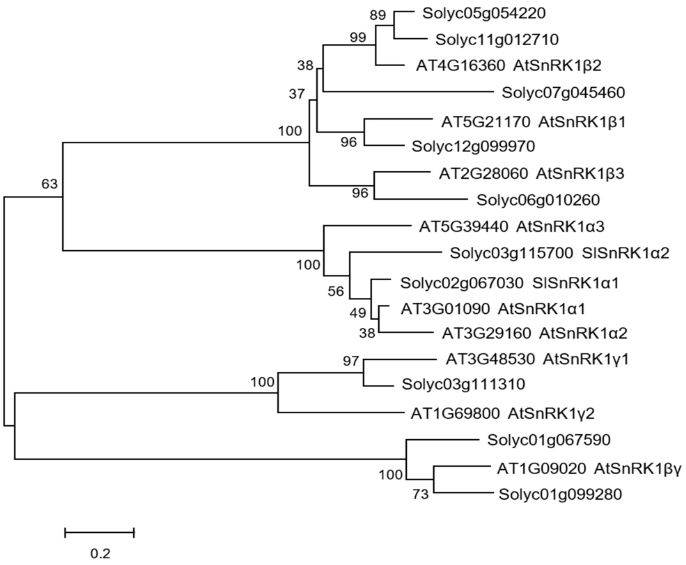

Supplementary Fig. S1 A phylogenetic analysis of the SnRK1 family between Arabidopsis and tomato. Homologs of SnRK1 proteins were identified using the Sol genomics network (http://solgenomics.net/) for tomato and TAIR (www.arabidopsis.org) for Arabidopsis. A phylogenetic tree was reconstructed based on their amino acid sequences using the maximum-likelihood method in MEGA5.0 software. The scale bar indicates the estimated number of amino acid substitutions per site. Bootstrap analysis of 1000 replications provided a reliable estimation of the topology of phylogenetic trees.

Supplementary Fig. S2

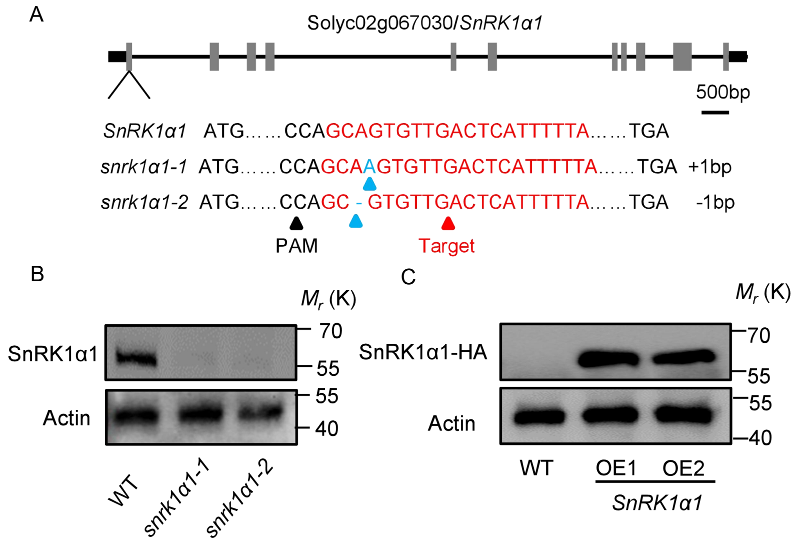

Supplementary Fig. S2 Identification of *snrk1α1* mutants and *SnRK1α1*-overexpression (*SnRK1α1* OE) plants. (A) Identification of *snrk1α1* mutants, DNA sequence comparison of wild type (WT), *snrk1α1-1*, and *snrk1α1-2*. The different rectangles represent the UTR regions and the exon regions. Single guide RNAs (sgRNAs), protospacer-adjacent motifs (PAMs) and Cas9 cutting sites are indicated by different arrows. (B) Comparison of SnRK1α1 protein levels between wild type (WT) and *snrk1α1* mutants**.** Actin was used as a loading control. (C) Immunoblotting analysis of SnRK1α1 protein in WT and *SnRK1α1* OE plants using an anti HA antibody. Actin was used as a loading control.

Supplementary Fig. S3

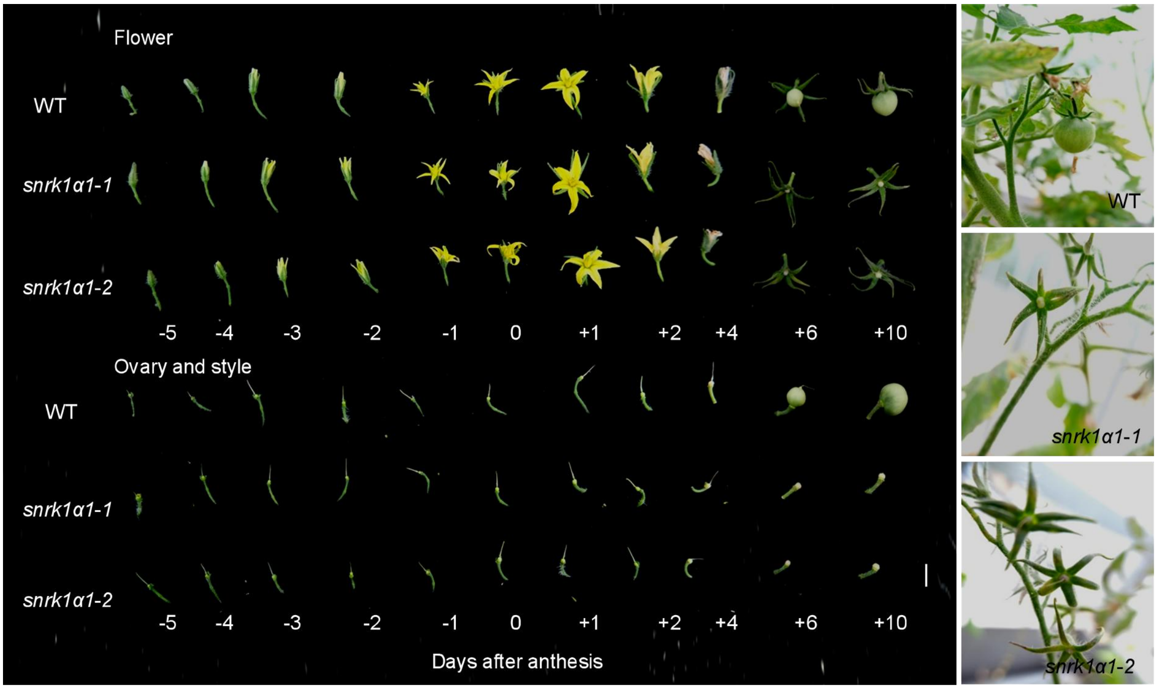

Supplementary Fig. S3 Phenotypes of tomato flowers, ovary, and styles from the flower bud to fruitlet stages in wild type (WT) and *snrk1α1* mutants. Bars: 1cm. Images were digitally extracted for comparison.

Supplementary Fig. S4

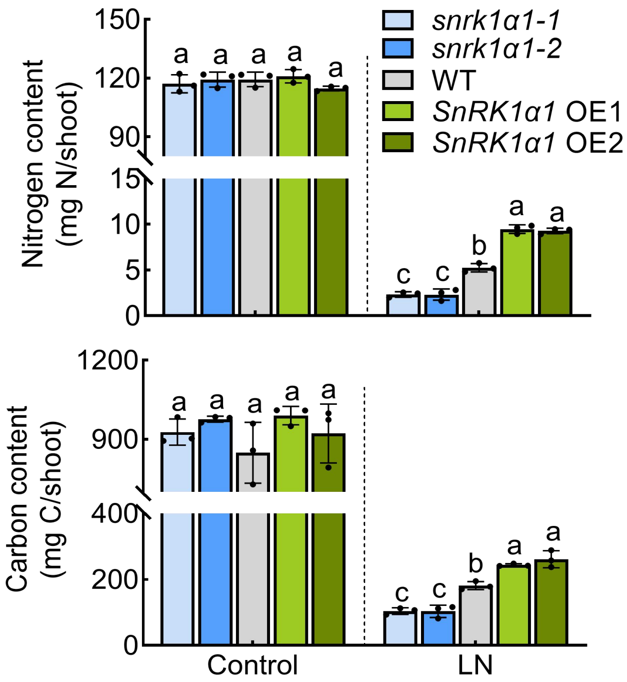

Supplementary Fig. S4 Nitrogen and carbon contents of shoots in wild type (WT), *snrk1α1*, and *SnRK1α1*-overexpression (*SnRK1α1* OE) plants. Shoot nitrogen (upper panel) and carbon (lower panel) contents were calculated by multiplying the dry weight of shoots by their nitrogen and carbon concentrations, respectively. Error bars represent SD; data are means ± SD from n= 3 biological replicates (individual dots). Experiments were repeated three times with similar results. Distinct letters above the bars signify significant differences at the $P < 0.05$ level, as determined by one-way ANOVA analysis with Tukey's multiple comparison test. Precise *P*-values from these statistical tests are detailed in the Supplementary Data Set 10.

Supplementary Fig. S5

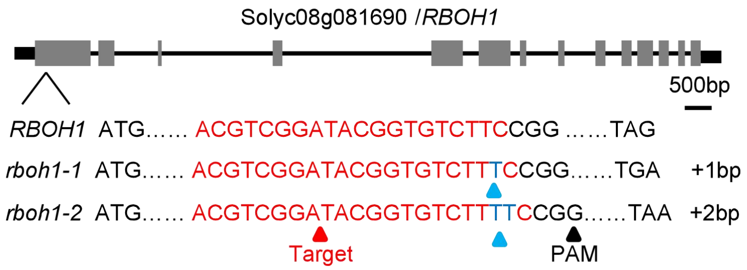

Supplementary Fig. S5 Identification of *rboh1* mutants. DNA sequence comparison of wild type (WT), *rboh1-1*, and *rboh1-2*. The different rectangles represent the UTR regions and the exon regions. Single guide RNAs (sgRNAs), protospacer-adjacent motifs (PAMs) and Cas9 cutting sites are indicated by different arrows.

Supplementary Fig. S6

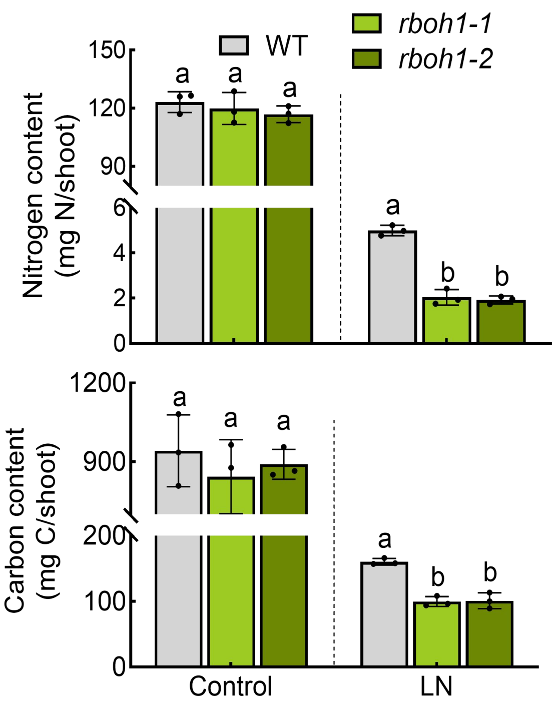

Supplementary Fig. S6 Nitrogen and carbon contents of shoots in wild type (WT), *rboh1-1*, and *rboh1-2* mutants. Shoot nitrogen (upper panel) and carbon (lower panel) contents were calculated by multiplying the dry weight of shoots by their nitrogen and carbon concentrations, respectively. Error bars represent SD; data are means ± SD from n= 3 biological replicates (individual dots). Experiments were repeated three times with similar results. Distinct letters above the bars signify significant differences at the $P < 0.05$ level, as determined by one-way ANOVA analysis with Tukey's multiple comparison test. Precise *P*-values from these statistical tests are detailed in the Supplementary Data Set 10.

Supplementary Fig. S7

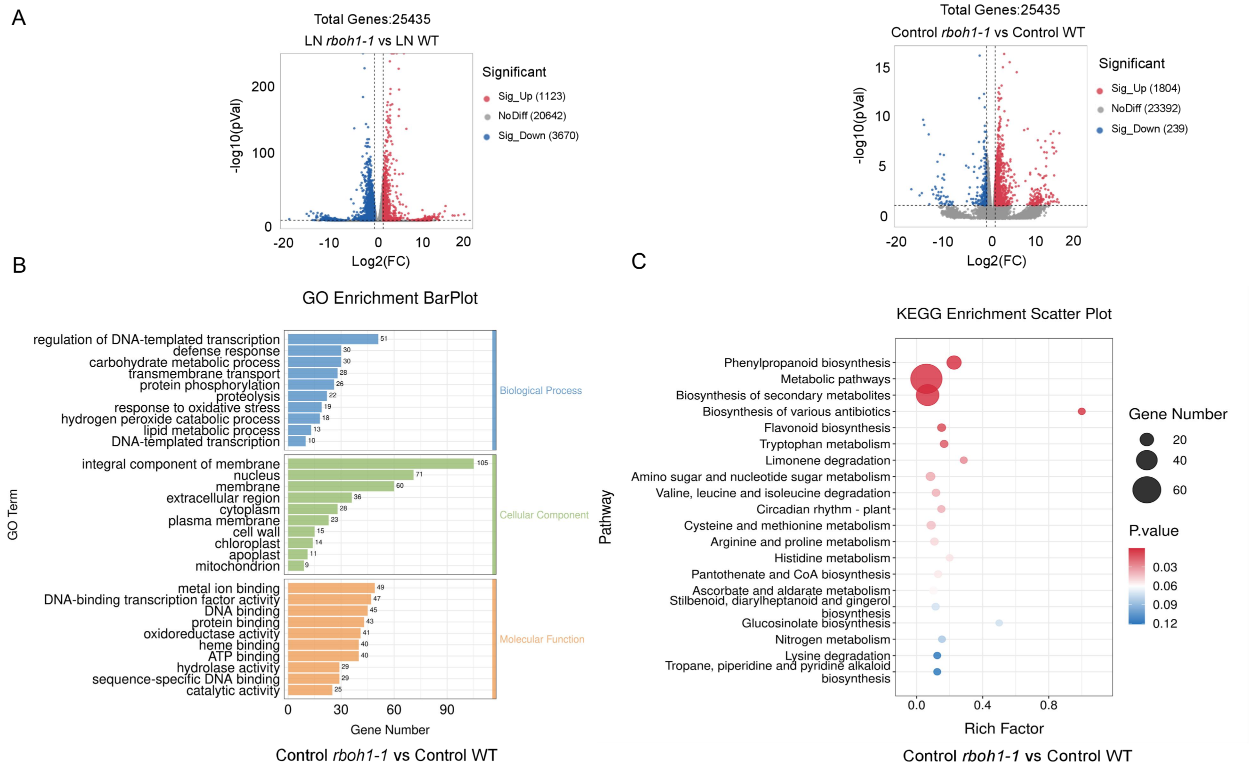

Supplementary Fig. S7 RNA-seq analysis of wild type (WT) and *rboh1-1* roots under control and low nitrogen conditions. (A) Volcano plot of differentially expressed genes (DEGs) comparing LN *rboh1-1* vs LN WT and Control *rboh1-1* vs Control WT. (B) Gene Ontology (GO) annotation and categorization of DEGs in comparisons of Control *rboh1-1* vs Control WT. (C) Kyoto Encyclopedia of Genes and Genomes (KEGG) enrichment analysis of DEGs in comparisons of Control *rboh1-1* vs Control WT. Data are provided in the Supplementary Data Set 3 and 7-9.

Supplementary Fig. S8

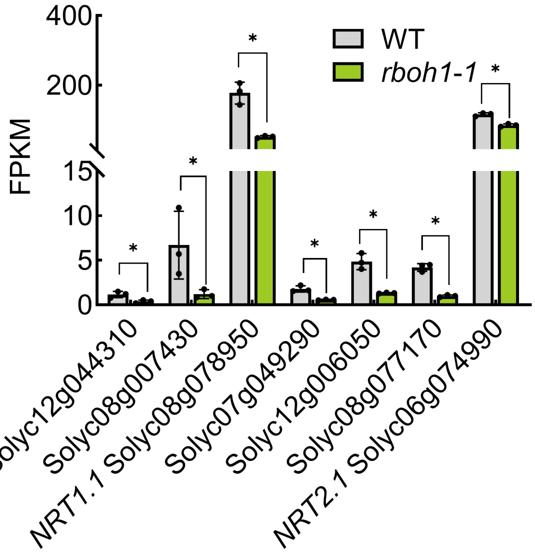

Supplementary Fig. S8 Fragments Per Kilobase of transcript per Million mapped reads (FPKM) of seven nitrate transporters (*NRTs*) in RNA-seq between wild type (WT) and *rboh1-1*. *NRTs* that were significantly differentially expressed between WT and *rboh1-1* are shown. Error bars represent SD; data are means ± SD of n= 3 biological replicates (individual dots). Asterisks above the bars denote a significant difference at the $P < 0.05$ level, as determined by Student's t-tests, compared to WT. Data are provided in the Supplementary Data Set 5.

Supplementary Fig. S9

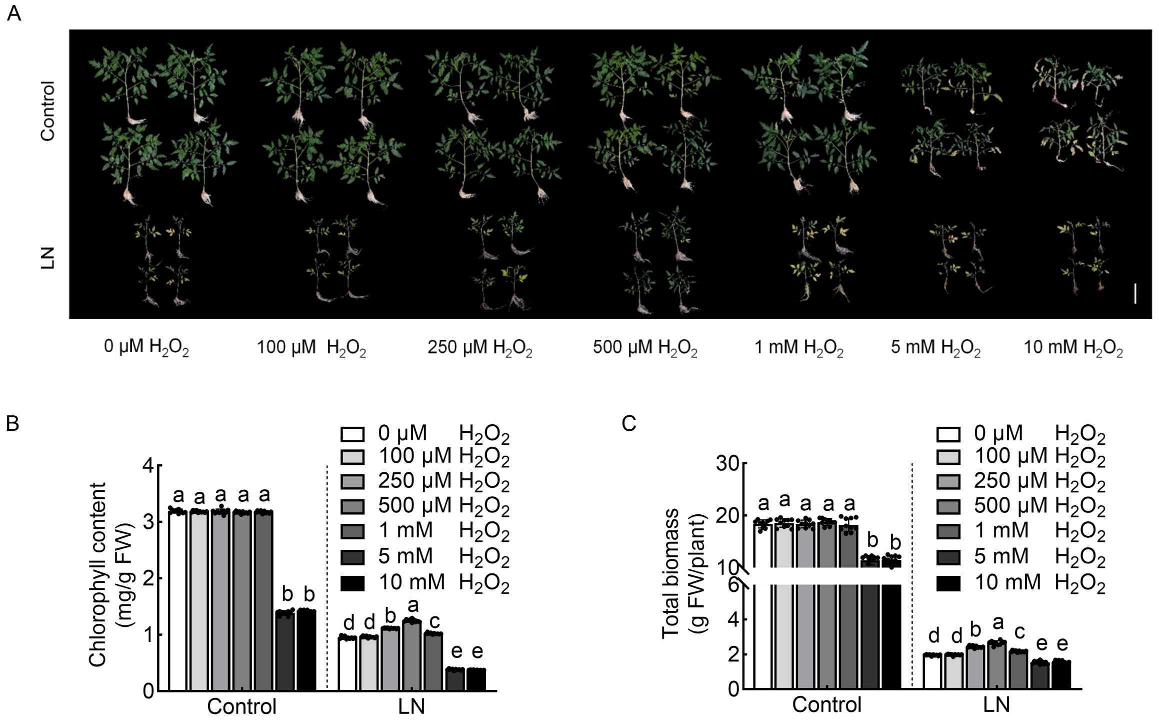

Supplementary Fig. S9 Treatment with different H_2O_2 concentrations in wild type (WT). (A) Phenotypes of WT plants under low nitrogen stress with various H_2O_2 concentrations (0 μM, 100 μM, 250 μM, 500 μM, 1 mM, 5 mM, and 10 mM). Images were digitally extracted for comparison. Bars: 10 cm. (B) Chlorophyll content and (C) biomass of WT plants under low nitrogen stress with different H_2O_2 concentrations. Error bars represent SD; data are means ± SD from n= 10 biological replicates (individual dots). Experiments were repeated three times with similar results. Distinct letters above the bars signify significant differences at the $P < 0.05$ level, as determined by one-way ANOVA with Tukey's multiple comparison test. Precise *P*-values from these statistical tests are detailed in the Supplementary Data Set 10.

Supplementary Fig. S10

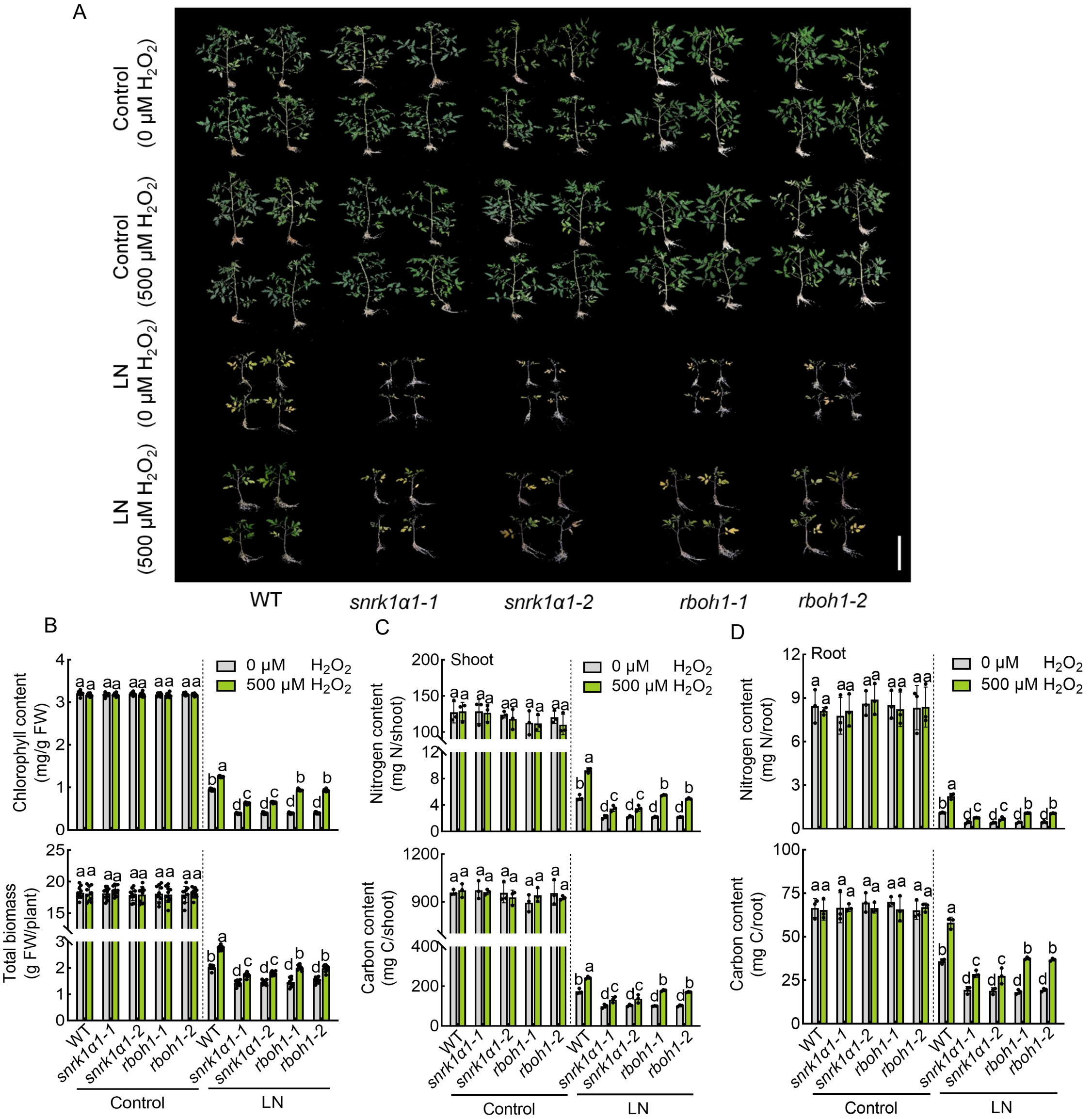

Supplementary Fig. S10 H_2O_2 treatment in different genotypes of tomato plants. (A) Phenotype of wild type (WT), *snrk1α1-1*, *snrk1α1-2*, *rboh1-1*, *rboh1-2* plants under low nitrogen stress and varying H_2O_2 concentrations (0 μM, 500 μM). Images were digitally extracted for comparison. Bars: 10 cm. (B) Chlorophyll content (upper panel) and biomass (lower panel) of WT, *snrk1α1-1*, *snrk1α1-2*, *rboh1-1*, and *rboh1-2* plants under low nitrogen stress and different H_2O_2 concentrations (0 μM, 500 μM). (C) Nitrogen (upper panel) and carbon (lower panel) contents of shoots and (D) roots in WT, , *snrk1α1-1*, *snrk1α1-2*, *rboh1-1*, and *rboh1-2* plants under low nitrogen stress and varying H_2O_2 concentrations. Nitrogen and carbon contents were calculated based on the dry weight of the shoots and roots multiplied by their nitrogen and carbon concentrations. Error bars represent SD; data are means ± SD of n= 3 biological replicates in (C, D); n= 10 biological replicates in (A, B). Experiments were repeated three times with similar results. Distinct letters above the bars signify significant differences at the *P* < 0.05 level, as determined by two-way ANOVA analysis with Tukey's multiple comparison test. Precise *P*-values from these statistical tests are detailed in the Supplementary Data Set 10.

Supplementary Fig. S11

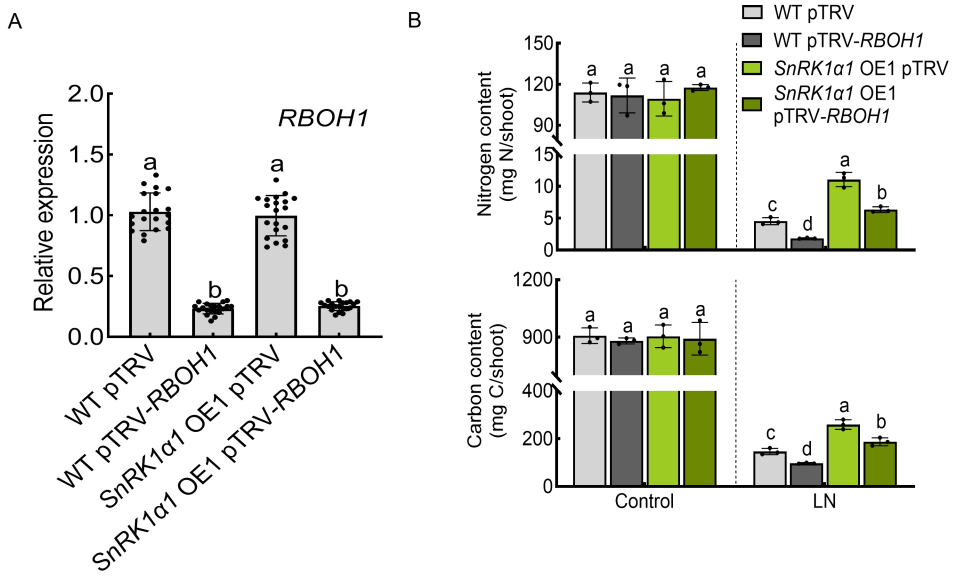

Supplementary Fig. S11 Relative expression of *RBOH1* and nitrogen and carbon contents of roots in different genotypes of tomato plants. (A) Relative expression of *RBOH1* in different genotypes of tomato plants. Silencing *RBOH1* resulted in a 70–80% reduction in *RBOH1* transcript levels. (B) Nitrogen (upper panel) and carbon (lower panel) content of shoots in WT pTRV, WT pTRV-*RBOH1*, *SnRK1α1* OE1 pTRV, *SnRK1α1* OE1 pTRV-*RBOH1* plants. Shoot nitrogen and carbon contents were calculated based on the dry weight of the shoots multiplied by their nitrogen and carbon concentrations. Error bars represent SD; data are means ± SD of 20 biological replicates in (A), 3 biological replicates in (B). Experiments were repeated three times with similar results. Distinct letters above the bars signify significant differences at the $P < 0.05$ level, as determined by one-way ANOVA analysis with Tukey's multiple comparison test. Precise *P*-values from these statistical tests are detailed in the Supplementary Data Set 10.

Supplementary Fig. S12

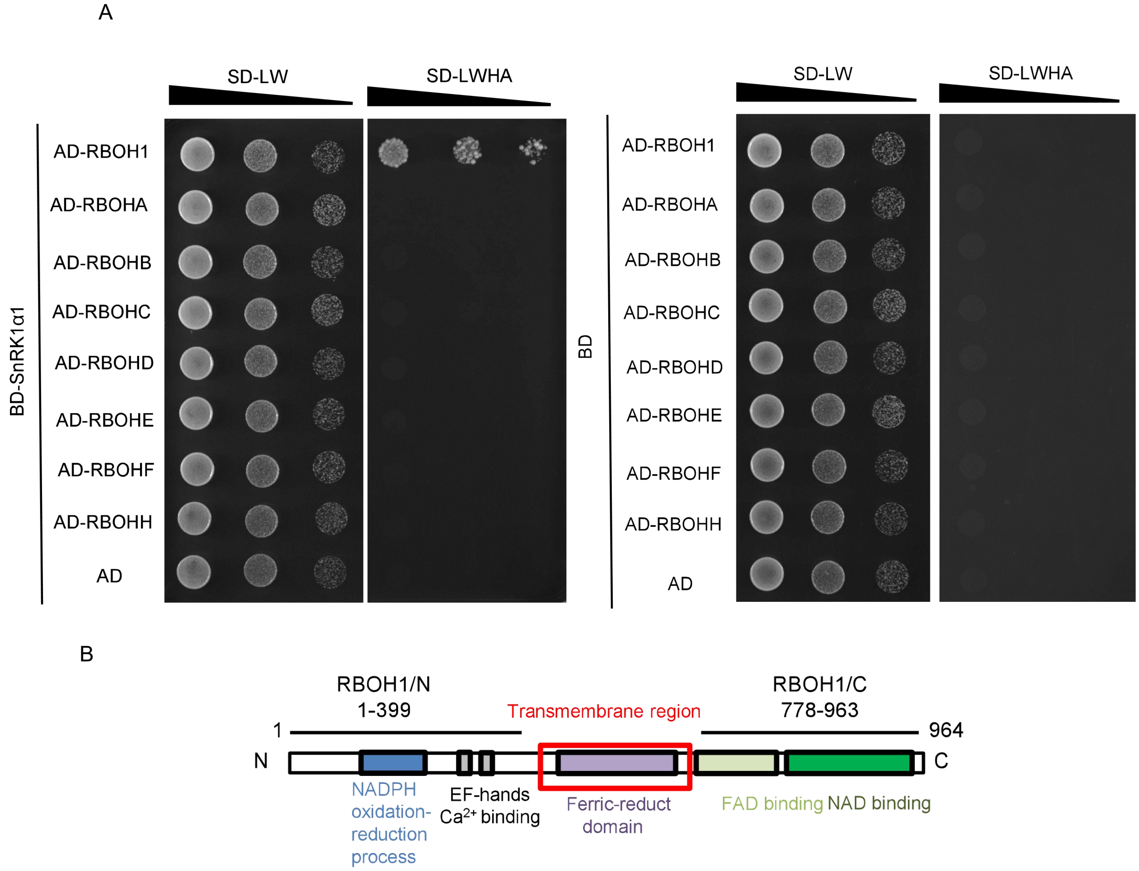

Supplementary Fig. S12 Yeast two-hybrid assays were conducted to test the interaction between SnRK1α1 and RBOHs. (A) Yeast two-hybrid assays showing the interaction between SnRK1α1 and RBOHs. Yeast grown on SD/-Leu/-Trp (-LW) for 2 days or SD/-Leu/-Trp/-Ade/-His (-LWAH) medium for 5 days. (B) Schematic diagram of the RBOH1 protein domains. Experiments in (A) were repeated three times with similar results.

Supplementary Fig. S13

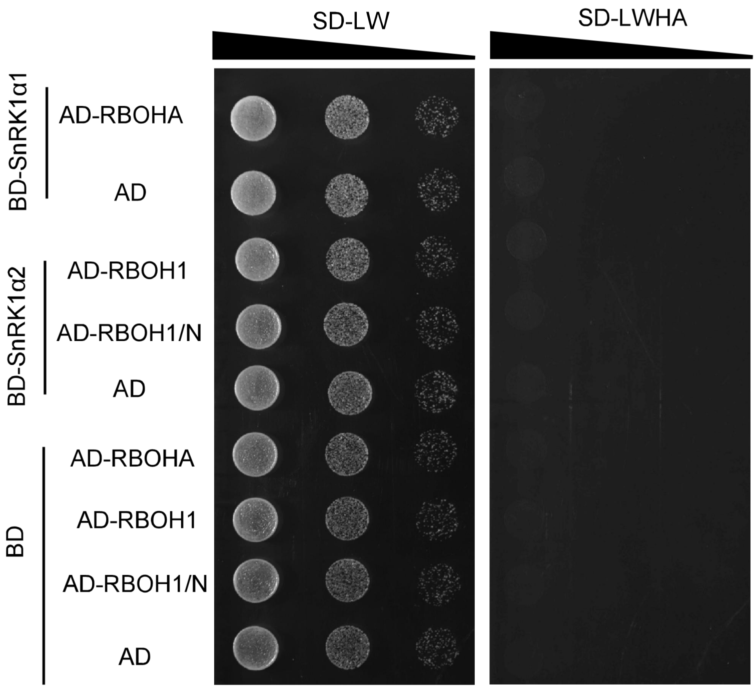

Supplementary Fig. S13 Yeast two-hybrid assays were conducted to test the interactions between SnRK1α1 and RBOHA, as well as SnRK1α2 and RBOH1 or RBOH1/N. Yeast grown on SD/-Leu/-Trp (-LW) for 2 days or SD/-Leu/-Trp/-Ade/-His (-LWAH) medium for 5 days. Experiments were repeated three times with similar results.

Supplementary Fig. S14

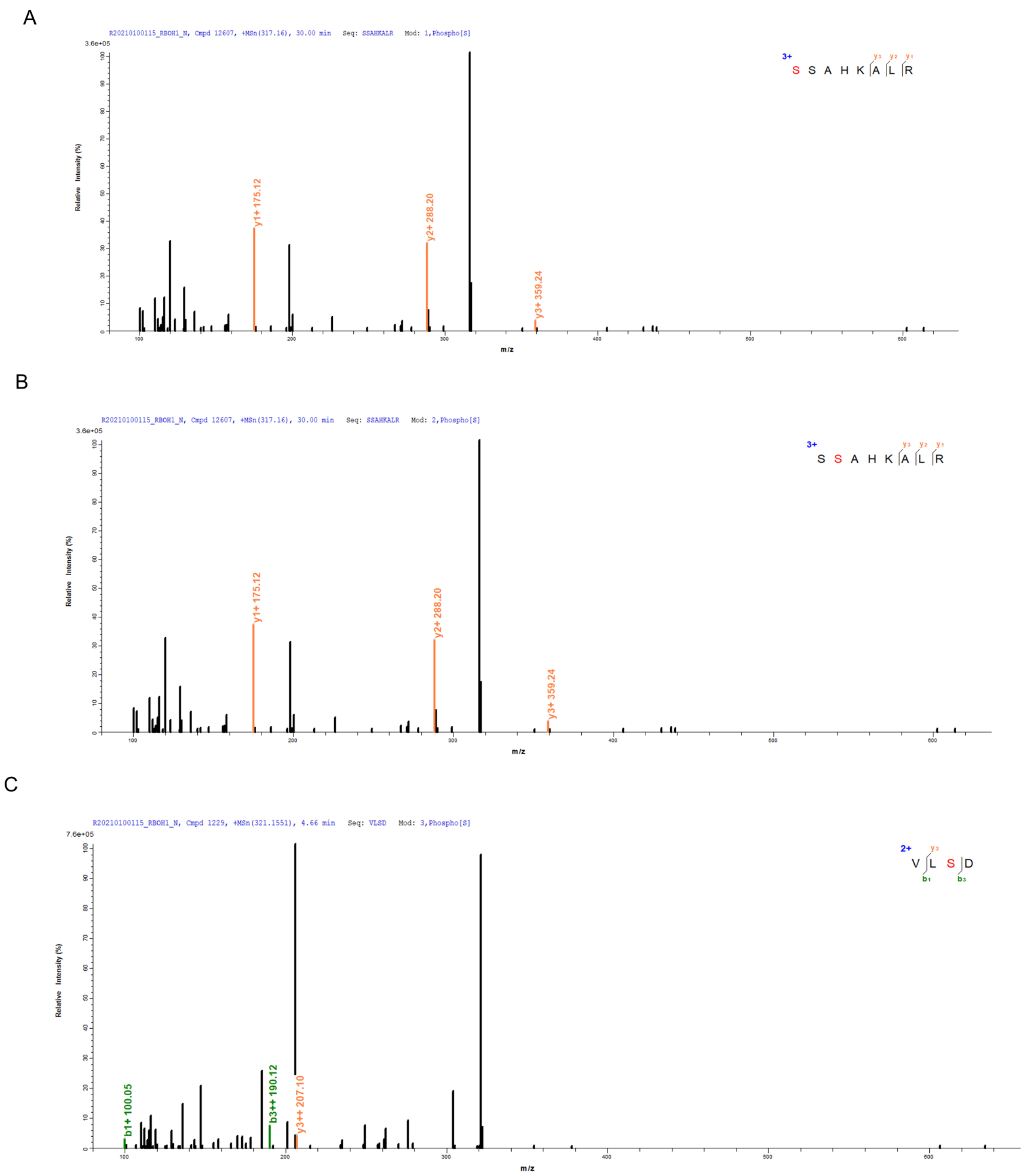

Supplementary Fig. S14 Identification of SnRK1α1-targeted phosphorylation sites on RBOH1/N. (A), (B) and (C) LC-MS/MS analysis identifying phosphorylation sites on RBOH1/N. Mass spectrometry results show phosphorylation at Serine (S)188, S189, and S308 residues of RBOH1/N.

Supplementary Fig. S15

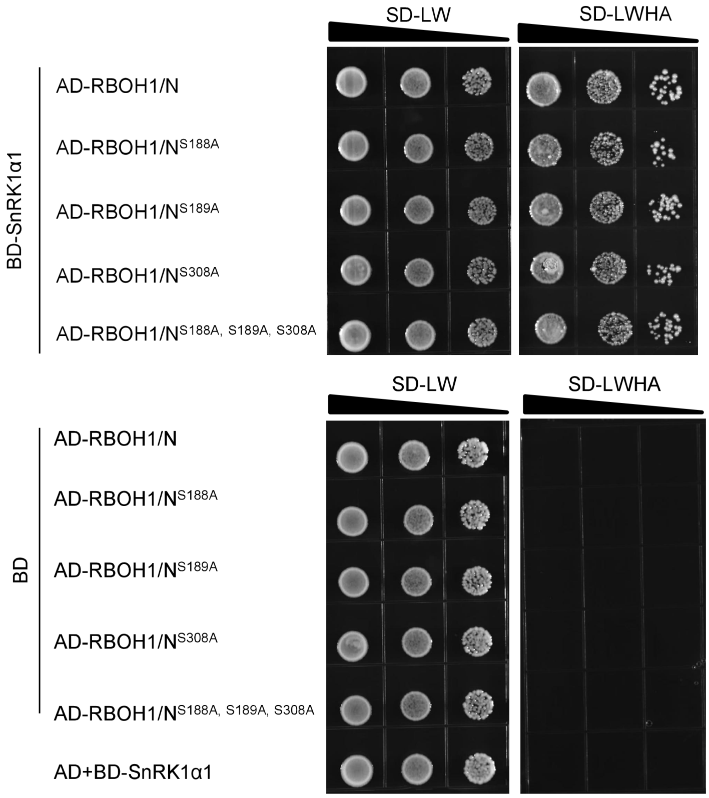

Supplementary Fig. S15 Yeast two-hybrid assays were conducted to test the interaction between SnRK1α1 and RBOH1/N or its variants. Yeast grown on SD/-Leu/-Trp (-LW) for 2 days or SD/-Leu/-Trp/-Ade/-His (-LWAH) medium for 5 days. Experiments were repeated three times with similar results.

Supplementary Fig. S16

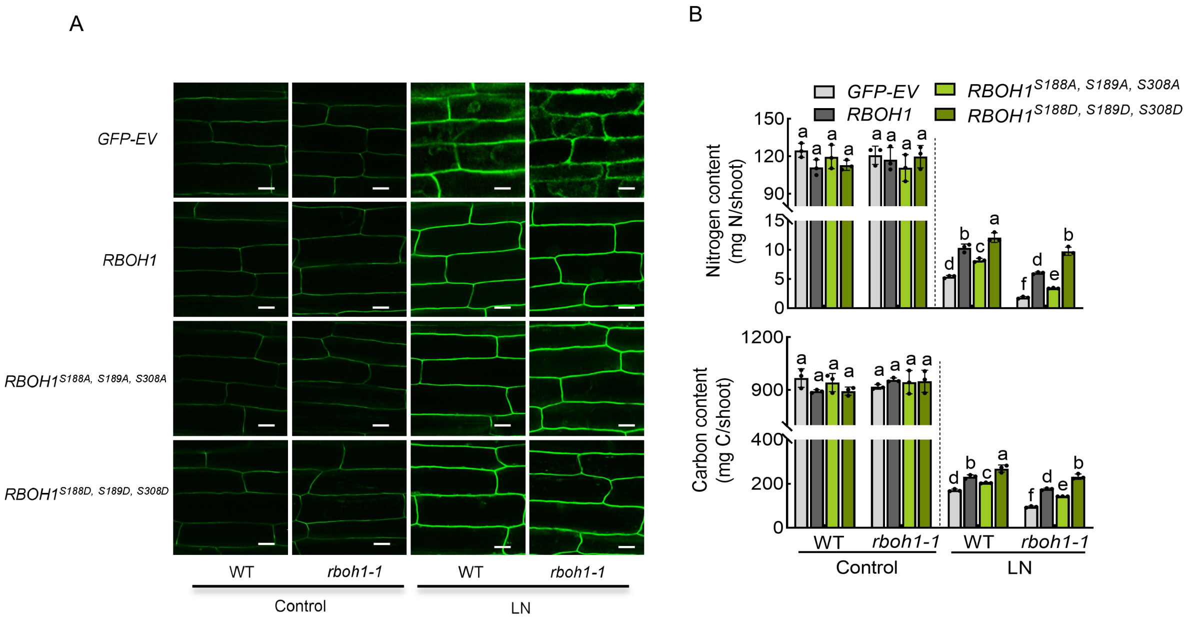

Supplementary Fig. S16 GFP-tagged protein abundance and nitrogen and carbon contents of roots in different transgenic tomato plants. (A) Expression of GFP-tagged constructs: *proRBOH1:GFP-EV* (empty vector) (*GFP-EV*), *proRBOH1:GFP-RBOH1* (*RBOH1*), *proRBOH1: RBOH1*$^{S188A,\ S189A,\ S308A}$ (*RBOH1*$^{S188A,\ S189A,\ S308A}$) and phospho-mimic variants *proRBOH1:GFP-RBOH1*$^{S188D,\ S189D,\ S308D}$ (*RBOH1*$^{S188D,\ S189D,\ S308D}$) in WT and *rboh1-1* mutants. Bars: 25μm**.** (B) Nitrogen (upper panel) and carbon (lower panel) contents of shoots in different transgenic tomato plants. Shoot nitrogen and carbon contents were calculated based on the dry weight of the shoots multiplied by their nitrogen and carbon concentrations. Error bars represent SD; data are means ± SD (n= 3 biological replicates). Experiments were repeated three times with similar results. Distinct letters above the bars signify significant differences at the $P < 0.05$ level, as determined by one-way ANOVA analysis with Tukey's multiple comparison test. Precise *P*-values from these statistical tests are detailed in the Supplementary Data Set 10.

Supplementary Fig. S17

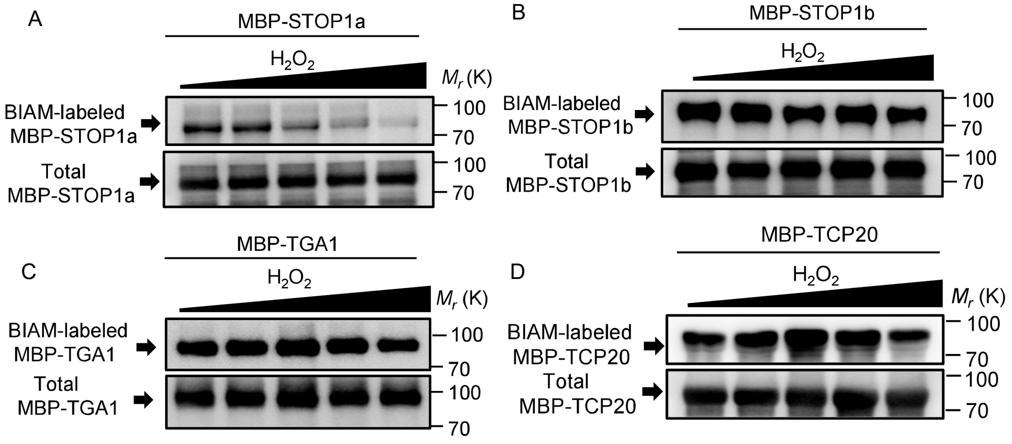

Supplementary Fig. S17 Analysis of oxidative modification of STOP1a, STOP1b, TGA1, TCP20 by BIAM-labeling assay. (A) MBP-STOP1a, (B) MBP-STOP1b, (C) MBP-TGA1, (D) MBP-TCP20 proteins were pretreated with different concentrations of H_2O_2 (0μM, 1μM, 10μM, 100μM, 1mM), and then labeled by BIAM at room temperature for 30 min. Labeled proteins were separated by SDS-PAGE and detected by western blotting with HRP-conjugated streptavidin and anti-MBP antibodies. Experiments were repeated three times with similar results.

Supplementary Fig. S18

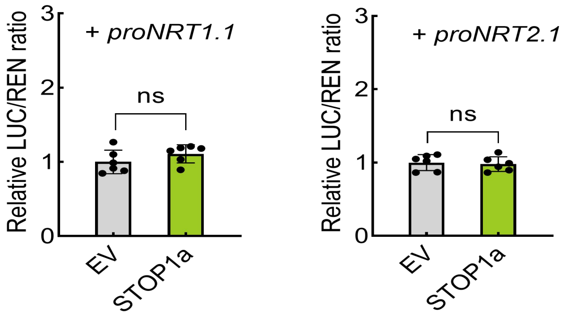

Supplementary Fig. S18 Regulatory effects of STOP1a on the promoters of *NRT1.1* and *NRT2.1* as determined by dual-luciferase assays. The ratios of firefly luciferase/Renilla luciferase (LUC/REN) of the empty vector (EV) plus promoters under normal conditions were set as '1'. Error bars represent SD; data are means ± SD (n= 6 biological replicates). Experiments were repeated three times with similar results. 'ns' stands for 'not significant', indicating that the differences between the groups are not statistically significant at the $P < 0.05$ level, as determined by Student's t-tests. Precise *P*-values from these statistical tests are detailed in the Supplementary Data Set 10.

Supplementary Fig. S19

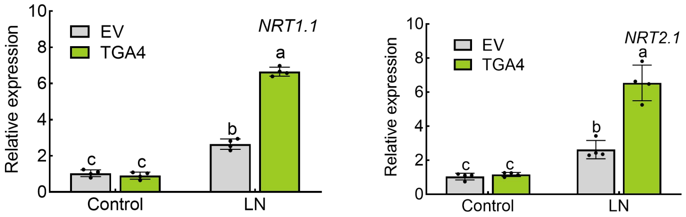

Supplementary Fig. S19 Relative expression of *NRT1.1* and *NRT2.1* in the WT *GFP-EV* and WT *GFP-TGA4* transgenic roots under control and low-nitrogen conditions. The relative expression of WT *GFP-EV* (EV) under control conditions was set to '1' . Error bars represent SD; data are means ± SD of 4 biological replicates. Experiments were repeated three times with similar results. Distinct letters above the bars signify significant differences at the $P < 0.05$ level, as determined by two-way ANOVA analysis with Tukey's multiple comparison test. Precise *P*-values from these statistical tests are detailed in the Supplementary Data Set 10.

Supplementary Fig. S20

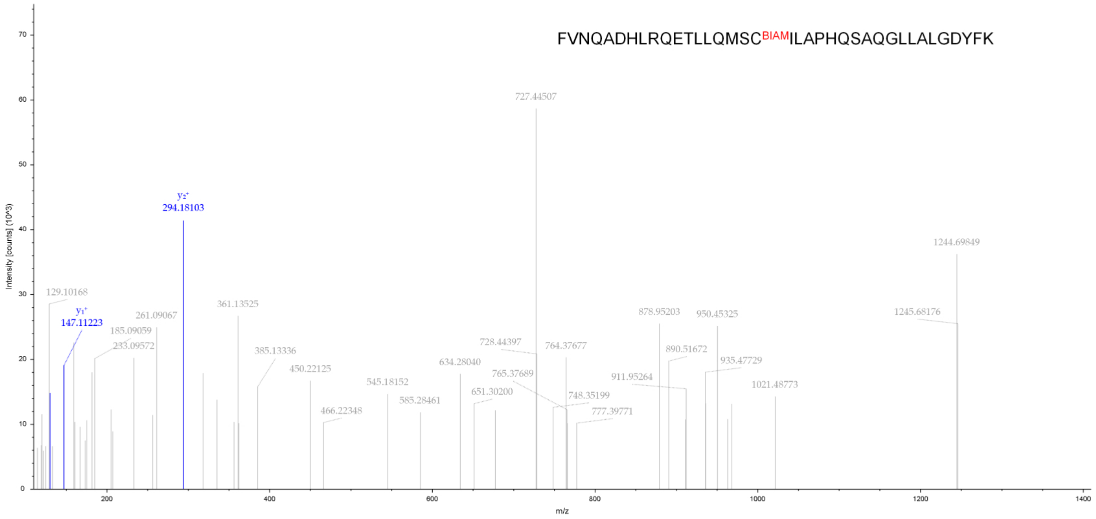

Supplementary Fig. S20 Mass spectrometry analysis of tryptic fragments of MBP-TGA4 protein treated as in Figure 8F. Cysteine (C) 334 modified with BIAM was identified as an H_2O_2-sensitive residue.

Supplementary Fig. S21

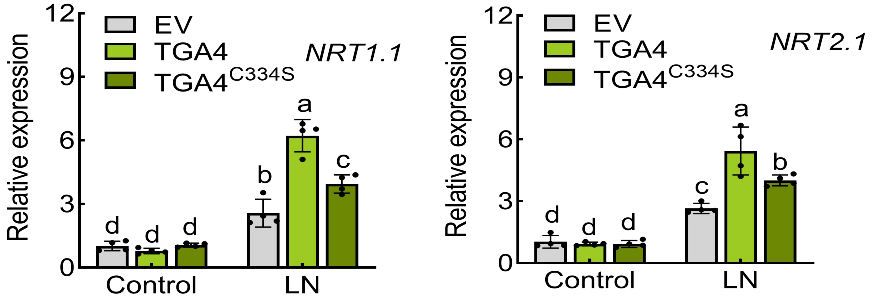

Supplementary Fig. S21 Relative expression of *NRT1.1* and *NRT2.1* in the WT *GFP-EV* (EV), WT *GFP-TGA4* (TGA4) and WT *GFP-TGA4^{C334S}* (TGA4^{C334S}) transgenic roots under control and low-nitrogen conditions. The relative expression of EV under control conditions was set to ‘1’ . Error bars represent SD; data are means ± SD of 4 biological replicates. Experiments were repeated three times with similar results. Distinct letters above the bars signify significant differences at the *P* < 0.05 level, as determined by two-way ANOVA analysis with Tukey's multiple comparison test. Precise *P*-values from these statistical tests are detailed in the Supplementary Data Set 10.

Supplementary Fig. S22

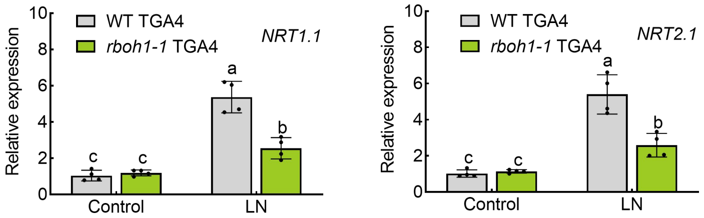

Supplementary Fig. S22 Relative expression of *NRT1.1* and *NRT2.1* in the WT *GFP-TGA4* (WT TGA4) and *rboh1-1 GFP-TGA4* (*rboh1-1* TGA4) transgenic roots under control and low-nitrogen conditions. The relative expression of WT TGA4 under control conditions was set to ‘1’ . Error bars represent SD; data represent the means ± SD of 4 biological replicates. Experiments were repeated three times with similar results. Distinct letters above the bars signify significant differences at the $P < 0.05$ level, as determined by two-way ANOVA analysis with Tukey's multiple comparison test. Precise *P*-values from these statistical tests are detailed in the Supplementary Data Set 10.

Supplementary Fig. S23

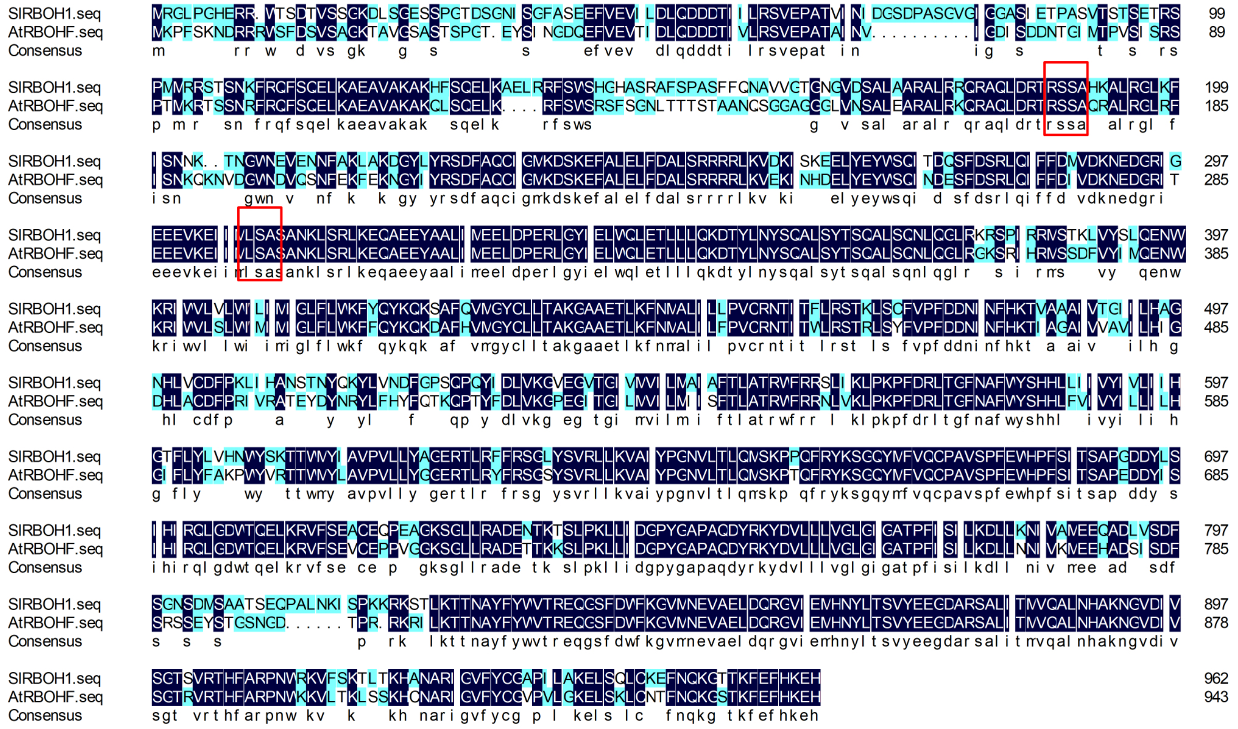

Supplementary Fig. S23 Comparison of amino acid sequences between tomato RBOH1 (SlRBOH1) and Arabidopsis RBOHF (AtRBOHF). The three phosphorylation sites Serine (S)188, S189, and S308 on RBOH1 mediated by SnRK1α1 are highlighted in boxes.

Supplementary Fig. S24

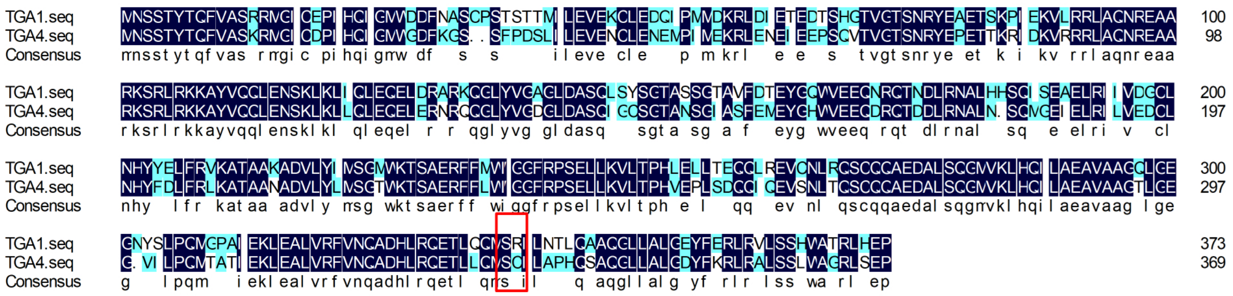

Supplementary Fig. S24 Comparison of amino acid sequence between TGA1 and TGA4 in tomato.

The Oxidation site Cysteine (C) 334 on TGA4 mediated by H_2O_2 is highlighted in box.

Supplementary Table S1. Target sequences used for CRISPR-Cas9 -mediated gene editing

Name of sgRNA	Sequence
SnRK1α1-sgRNA	TAAAAATGAGTCAACACTGC
RBOH1-sgRNA	ACGTCGGATACGGTGTCTTC

Supplementary Table S2. Primers used for plasmid constructions

Name	Sequence
SnRK1α1-HA-F	ttacaattaccatggggcgcgccATGGACGGAACAGCAGTGCA
SnRK1α1-HA-R	aacatcgtatgggtaggtaccAAGTACTCGAAGCTGAGCAAGAAA
TRV2-RBOH1-F	gtgagtaaggttaccATGAGGGGTTTACCTGGGCA
TRV2-RBOH1-R	cgtgagctcggtaccCGGCGATCGAGTTTCCGA
pGBKT7-SnRK1α1-F	atggccatggaggccgaattcATGGACGGAACAGCAGTGCA
pGBKT7-SnRK1α1-R	ccgctgcaggtcgacggatccAAGTACTCGAAGCTGAGCAAGAAA
pGBKT7-SnRK1α2-F	atggccatggaggccgaattcATGAGTTCCAGAGGTGGTGGAA
pGBKT7-SnRK1α2-R	ccgctgcaggtcgacggatccTTGTGGCCCCTCTAGCTGC
pGADT7-RBOH1-F	gccatggaggccagtgaattcATGAGGGGTTTACCTGGGCA
pGADT7-RBOH1-R	cagctcgagctcgatggatccAAAATGTTCTTTGTGAAACTCGAACT
pGADT7-RBOH1/N-F	gccatggaggccagtgaattcATGAGGGGTTTACCTGGGCA
pGADT7-RBOH1/N-R	cagctcgagctcgatggatccTCTCTTCCAATTCTCTTGCAGTGA
pGADT7-RBOH1/C-F	gccatggaggccagtgaattcATTGGAAGAGAATTTGGGTTCTGG
pGADT7-RBOH1/C-R	cagctcgagctcgatggatccAAAATGTTCTTTGTGAAACTCGAACT
pGADT7-RBOHA-F	gccatggaggccagtgaattcATGGAGATCGAAAACACGACAGA
pGADT7-RBOHA-R	cagctcgagctcgatggatccGAAATTTTCTTTATGAAATTCAAACTTTG
pGADT7-RBOHB-F	gccatggaggccagtgaattcATGCAAAATTCGGAAAATCATCA
pGADT7-RBOHB-R	cagctcgagctcgatggatccAAAATTTTCTTTATGGAAATCAAACTTG
pGADT7-RBOHC-F	gccatggaggccagtgaattcATGCAGTTAATGTCACTTTTTAGGTCA
pGADT7-RBOHC-R	cagctcgagctcgatggatccTACTTGAGATTGTAGAAATTCTTTACCTACTG
pGADT7-RBOHD-F	gccatggaggccagtgaattcATGCAAAATCCAGAAGATCACCA
pGADT7-RBOHD-R	cagctcgagctcgatggatccAAAGTTTTCTTTATGGAAATCAAACTTT
pGADT7-RBOHE-F	gccatggaggccagtgaattcATGGTGCCCATTGACGATGG
pGADT7-RBOHE-R	cagctcgagctcgatggatccGAAATTTTCTTTGTGGAAATTGAAAC
pGADT7-RBOHF-F	gccatggaggccagtgaattcATGTCGGAGGAGCAATGTTACC
pGADT7-RBOHF-R	cagctcgagctcgatggatccAAAGTACTCTTTGTGGAACTCAAATCG
pGADT7-RBOHH-F	gccatggaggccagtgaattcATGGCAAGGAGAAAGAAGATTAATG
pGADT7-RBOHH-R	cagctcgagctcgatggatccGAAGTTCTCTTTGTGGAAGTTGAAGC
pGADT7-RBOH1/N^{S188A}-F	ACTCGTgcaAGCGCACACAAGGCTCTTCGTGG
pGADT7-RBOH1/N^{S188A}-R	TGTGCGCTtgcACGAGTCCGGTCGAGCTGAGC
pGADT7-RBOH1/N^{S189A}-F	TCGTTCCgcaGCACACAAGGCTCTTCGTGGAC
pGADT7-RBOH1/N^{S189A}-R	TGTGTGCtgcGGAACGAGTCCGGTCGAGCTGA
pGADT7-RBOH1/N^{S308A}-F	TCATGCTAgcaGCCTCTGCAAACAAATTATCAAGA
pGADT7-RBOH1/N^{S308A}-R	AGAGGCtgcTAGCATGATGATCTCTTTTACTTCTTCTT
pGADT7-RBOH1/N$^{S188A, S189A}$-F	TCGTgcagcaGCACACAAGGCTCTTCGTGGACT
pGADT7-RBOH1/N$^{S188A, S189A}$-R	TTGTGTGCtgctgcACGAGTCCGGTCGAGCTGA
GST-SnRK1α1-F	gatctggttccgcgtggatccATGGACGGAACAGCAGTGCA
GST-SnRK1α1-R	ctcgagtcgacccgggaattcAAGTACTCGAAGCTGAGCAAGAAA
His-RBOH1/N-F	gccatggctgatatcggatccATGAGGGGTTTACCTGGGCA
His-RBOH1/N-R	gcaagcttgtcgacggagctcTCTCTTCCAATTCTCTTGCAGTGA
His-RBOH1/C-F	gccatggctgatatcggatccATGATTGGAAGAGAATTTGGGTTC
His-RBOH1/C-R	gcaagcttgtcgacggagctcAAAATGTTCTTTGTGAAACTCGAACT

Supplementary Table S2 continued	
His-RBOH1/N^{S188A}-F	ACTCGTgcaAGCGCACACAAGGCTCTTCGTGG
His-RBOH1/N^{S188A}-R	TGTGCGCTtgcACGAGTCCGGTCGAGCTGAGC
His-RBOH1/N^{S189A}-F	TCGTTCCgcaGCACACAAGGCTCTTCGTGGAC
His-RBOH1/N^{S189A}-R	TGTGTGCtgcGGAACGAGTCCGGTCGAGCTGA
His-RBOH1/N^{S308A}-F	TCATGCTAgcaGCCTCTGCAAACAAATTATCAAGA
His-RBOH1/N^{S308A}-R	AGAGGCtgcTAGCATGATGATCTCTTTTACTTCTTCTT
His-RBOH1/N$^{S188A, S189A}$-F	TCGTgcagcaGCACACAAGGCTCTTCGTGGACT
His-RBOH1/N$^{S188A, S189A}$-R	TTGTGTGCtgctgcACGAGTCCGGTCGAGCTGA
SnRK1α1-cLUC-F	ggggacaagtttgtacaaaaaagcaggctttATGGACGGAACAGCAGTGCA
SnRK1α1-cLUC-R	ggggaccactttgtacaagaaagctgggtcAAGTACTCGAAGCTGAGCAA
RBOH1/N-nLUC-F	ggggacaagtttgtacaaaaaagcaggctttATGAGGGGTTTACCTGGGCA
RBOH1/N-nLUC-R	ggggaccactttgtacaagaaagctgggtcTCTCTTCCAATTCTCTTGCA
cYFP-SnRK1α1-F	atttacgaacgatagttaattaacATGGACGGAACAGCAGTGCA
cYFP-SnRK1α1-R	actgccacctcctccactagtAAGTACTCGAAGCTGAGCAAGAAA
RBOH1-nYFP-F	atttacgaacgatagttaattaacATGAGGGGTTTACCTGGGCA
RBOH1-nYFP-R	actgccacctcctccactagtAAAATGTTCTTTGTGAAACTCGAACT
RBOH1/N-nYFP-F	atttacgaacgatagttaattaacATGAGGGGTTTACCTGGGCA
RBOH1/N-nYFP-R	actgccacctcctccactagtTCTCTTCCAATTCTCTTGCAGTGA
RBOH1/C-nYFP-F	atttacgaacgatagttaattaacATGATTGGAAGAGAATTTGGGTTC
RBOH1/C-nYFP-R	actgccacctcctccactagtAAAATGTTCTTTGTGAAACTCGAACT
RBOHA-nYFP-F	atttacgaacgatagttaattaacATGGAGATCGAAAACACGACAGA
RBOHA-nYFP-R	actgccacctcctccactagtGAAATTTTCTTTATGAAATTCAAACTTTG
cYFP-SnRK1α2-F	atttacgaacgatagttaattaacATGAGTTCCAGAGGTGGTGGAA
cYFP-SnRK1α2-R	actgccacctcctccactagtTTGTGGCCCCTCTAGCTGC
RBOH1/N-GFP-F	ctctcgagctttcgcgagctcATGAGGGGTTTACCTGGGCA
RBOH1/N-GFP-R	gcccttgctcaccatggatccTCTCTTCCAATTCTCTTGCAGTGA
RBOH1/N^{S188A}-GFP-F	ACTCGTgcaAGCGCACACAAGGCTCTTCGTGG
RBOH1/N^{S188A}-GFP-R	TGTGCGCTtgcACGAGTCCGGTCGAGCTGAGC
RBOH1/N^{S189A}-GFP-F	TCGTTCCgcaGCACACAAGGCTCTTCGTGGAC
RBOH1/N^{S189A}-GFP-R	TGTGTGCtgcGGAACGAGTCCGGTCGAGCTGA
RBOH1/N^{S308A}-GFP-F	TCATGCTAgcaGCCTCTGCAAACAAATTATCAAGA
RBOH1/N^{S308A}-GFP-R	AGAGGCtgcTAGCATGATGATCTCTTTTACTTCTTCTT
RBOH1/N$^{S188A, S189A}$-GFP-F	TCGTgcagcaGCACACAAGGCTCTTCGTGGACT
RBOH1/N$^{S188A, S189A}$-GFP-R	TTGTGTGCtgctgcACGAGTCCGGTCGAGCTGA
proRBOH1:GFP -F	gaccatgattacgccaagcttGGCCATAACAATATTCATACATGTCG
proRBOH1:GFP -R	gcccttgctcaccatggtaccCTGTCCCAAATCAAATGACAATGA
proRBOH1:GFP-RBOH1-F	gacgagctgtacaagctcgagATGAGGGGTTTACCTGGGCA
proRBOH1:GFP-RBOH1-R	cgatcggggaaattcgagctcAAAATGTTCTTTGTGAAACTCGAACT
proRBOH1:GFP-RBOH1$^{S188A, S189A}$-F	TCGTgcagcaGCACACAAGGCTCTTCGTGGACT
proRBOH1:GFP-RBOH1$^{S188A, S189A}$-R	TTGTGTGCtgctgcACGAGTCCGGTCGAGCTGA
proRBOH1:GFP-RBOH1^{S308A}-F	TCATGCTAgcaGCCTCTGCAAACAAATTATCAAGA
proRBOH1:GFP-RBOH1^{S308A}-R	AGAGGCtgcTAGCATGATGATCTCTTTTACTTCTTCTT
proRBOH1:GFP-RBOH1$^{S188D, S189D}$-F	TCGTgacgacGCACACAAGGCTCTTCGTGGACT

**Supplementary Table S2 continued**	
proRBOH1:GFP-RBOH1$^{S188D, S189D}$-R	TTGTGTGCgtcgtcACGAGTCCGGTCGAGCTGA
proRBOH1:GFP-RBOH1^{S308D}-F	TCATGCTAgacGCCTCTGCAAACAAATTATCAAGA
proRBOH1:GFP-RBOH1^{S308D}-R	AGAGGCgtcTAGCATGATGATCTCTTTTACTTCTTCTT
MBP-TGA1-F	gagggaaggatttcagaattcATGAATTCTTCAACATACACTCAGTTTG
MBP-TGA1-R	caggtcgactctagaggatccGGCCGGCTCATGAAGACG
MBP-TGA4-F	gagggaaggatttcagaattcATGAATTCTTCAACATATACTCAATTTGTT
MBP-TGA4-R	caggtcgactctagaggatccAGCAGGTTCAGAAAGACGTCCA
MBP-TGA4^{C334S}-F	GATGTCCAGCATACTGGCTCCGCACCAATCAG
MBP-TGA4^{C334S}-R	CCAGTATGCTGGACATCTGTAGAAGGGTTTCTTGG
MBP-TCP20-F	gagggaaggatttcagaattcATGGATCCCAAACAGGCTAACC
MBP-TCP20-R	caggtcgactctagaggatccATGTCCTGATCCTTGAGAATCCTC
MBP-STOP1a-F	gagggaaggatttcagaattcATGCCCTCAGATAACCATTCATTT
MBP-STOP1a-R	caggtcgactctagaggatccTTCCATTTCACTAGAACTCATAAATTTTG
MBP-STOP1b-F	gagggaaggatttcagaattcATGGAACAACAGGCTAATCAAAGC
MBP-STOP1b-R	caggtcgactctagaggatccTATTTCCAGCCTTTGTAGTTGCAT
ACC1-GFP-F	ctctcgagctttcgcgagctcATGAGATCCAGCATGTCTGGCT
ACC1-GFP-R	gcccttgctcaccatggatccTACTAGGTGCAAGCCAGACATGC
TGA4-GFP-F	ctctcgagctttcgcgagctcATGAATTCTTCAACATATACTCAATTTGTT
TGA4-GFP-R	gcccttgctcaccatggatccAGCAGGTTCAGAAAGACGTCCA
TGA4^{C334S}-GFP-F	GATGTCCAGCATACTGGCTCCGCACCAATCAG
TGA4^{C334S}-GFP-R	CCAGTATGCTGGACATCTGTAGAAGGGTTTCTTGG
SK-STOP1a-F	cgctctagaactagtggatccATGCCCTCAGATAACCATTCATTT
SK-STOP1a-R	tgatttcagcgaattggtaccTTCCATTTCACTAGAACTCATAAATTTTG
SK-TGA4-F	cgctctagaactagtggatccATGAATTCTTCAACATATACTCAATTTGTT
SK-TGA4-R	tgatttcagcgaattggtaccAGCAGGTTCAGAAAGACGTCCA
SK-TGA4^{C334S}-F	GATGTCCAGCATACTGGCTCCGCACCAATCAG
SK-TGA4^{C334S}-R	CCAGTATGCTGGACATCTGTAGAAGGGTTTCTTGG
proNRT1.1-LUC-F	gtcgacggtatcgataagcttTAAATAAAAATCTCTTATTAATATATTATTTTAAATCG
proNRT1.1-LUC-R	cgctctagaactagtggatccAAGTTGTATAGTTCCCGGGTATCTTG
proNRT2.1-LUC-F	gtcgacggtatcgataagcttAAGTTGACTTGGTTTCATTACATCAGA
proNRT2.1-LUC-R	cgctctagaactagtggatccCCAACCAATTATGGAAGCCTGA
proNRT1.1$^{-2000bp}$-LUC-F	gtcgacggtatcgataagcttGCCTAACAGTCTTTTATTAATAACAAAATAA
proNRT1.1$^{-2000bp}$-LUC-R	cgctctagaactagtggatccAAGATTCAATTGATTCTATAACCCTTTT
proNRT2.1$^{-2000bp}$-LUC-F	gtcgacggtatcgataagcttCCCTTTTAACTCCGCACTCCT
proNRT2.1$^{-2000bp}$-LUC-R	cgctctagaactagtggatccCCAACCAATTATGGAAGCCTGA

Supplementary Table S3. Primers used for RT-qPCR

Name	Sequence
SnRK1α1-F	TTATCAGCTGGTGCGAGGGA
SnRK1α1-R	GTGGAGGCACGGCCAAATAG
NRT1.1-F	CACATCGGAAAATTCGAGATCC
NRT1.1-R	GCCGCAATCATAGCTATAATCG
NRT2.1-F	CAAAGAATTGAAGGATCACCGG
NRT2.1-R	AGTGAAAAAGGAGATCCAGGAG
RBOH1-F	TCCAGCACAAGATTACCG
RBOH1-R	CCTCCATTGCGACGAT
RBOHA-F	CGAGAGTAGGATTCAGCGGT
RBOHA-R	TGCAGCTTCATGATCAGCAC
RBOHB-F	CAACATGAAGGCCATGGACGAA
RBOHB-R	CTGTGCCATATTTGGGCTAGCATT
RBOHC-F	TGAGCCACAGTACGCCTTTA
RBOHC-R	TAGCAAGCAACCACAGCAAG
RBOHD-F	AAGCCAACCGCATCCTCCTC
RBOHD-R	GCAGTAAGCTCGTCGAATCGC
RBOHE-F	CGGATGGAATCAGGCGCTAC
RBOHE-R	TCTCTGAAGATCCTCCATTAACTGC
RBOHF-F	ATCTGCACGAGAGGAAATGAAATCG
RBOHF-R	CAAACCGCGCCTCCACTTT
RBOHH-F	CCACGGCTGCTTCATATTCC
RBOHH-R	CGTGGTAGCGGTTCTCATTG
Ubiquitin3-F	GCCGACTACAACATCCAGAAGG
Ubiquitin3-R	TGCAACACAGCGAGCTTAACC
Actin-F	TGGTCGGAATGGGACAGAAG
Actin-R	CTCAGTCAGGAGAACAGGGT

Supplementary Table S4. Primers used for EMSA

Name	Sequence
NRT1.1-probe-F	TATCTTTTTGACGTGCAATA
NRT1.1-probe-R	TATTGCACGTCAAAAAGATA
NRT2.1-probe-F	AAACACACGTCAGGCTTCCA
NRT2.1-probe-R	TGGAAGCCTGACGTGTGTTT
Mu-NRT1.1-probe-F	TATCTTTTAAAAATGCAATA
Mu-NRT1.1-probe-R	TATTGCATTTTTAAAAGATA
Mu-NRT2.1-probe-F	AAACACAAAAAAGGCTTCCA
Mu-NRT2.1-probe-R	TGGAAGCCTTTTTTGTGTTT

Supplementary Table S5. Primers used for CUT&RUN

Name	Sequence
NRT1.1-CUT&RUN-F	CCCTATTGTTTGGGTGCATTCT
NRT1.1-CUT&RUN-R	GTCTGGCCCAGGAATTTTACAAC
NRT2.1-CUT&RUN-F	TTTGCAATGCCCTTATTCTTTG
NRT2.1-CUT&RUN-R	TCTTCACGTTTGCCTCACCC
