# Supplementary material for: SnRK1α1-mediated RBOH1 phosphorylation regulates reactive oxygen species to enhance tolerance to low nitrogen in tomato
Source: Plant Cell. 2024 Dec 12;37(1):koae321. doi: 10.1093/plcell/koae321 (PMC11684077; doi:10.1093/plcell/koae321)
Supplement: koae321_Supplementary_Data [file tpc.11290.2024-s20.pdf › tpc.11290.2024-s20.pdf]

THE PLANT CELL

SnRK1α1-RBOH1 regulates ROS production and the oxidation of TGA4 to enhance low nitrogen stress tolerance in tomato

Xue-Lian Zheng, Hongfeng Yang, Jinping Zou, Weiduo Jin, Zhen-Yu Qi, Ping Yang, Jingquan Yu, and Jie Zhou

Review Timeline:

Submission Date:	14-Feb-2024
Editorial Decision:	25-Mar-2024
Resubmission Received:	09-Sep-2024
Editorial Decision:	04-Oct-2024
Revision Received:	29-Oct-2024
Accepted:	04-Nov-2024

Prof. Dr. Jie Zhou
Zhejiang University
Horticulture
Hangzhou, Zhejiang
China

Dear Dr. Zhou:

Thank you for choosing to send your manuscript entitled "SnRK1.1-mediated RBOH1 phosphorylation positively regulates ROS production and tolerance to low nitrogen stress in Solanum lycopersicum L." for consideration at The Plant Cell. Your submission has been evaluated by members of the editorial board as well as expert reviewers in your field, and we regret to inform you that we are not able to recommend the publication of this manuscript in its current form. We have had input from multiple scientists and we have solicited post-review comments.

As you see, all the reviewers indicate that although the study is attractive and has novelty, the work needs additional experiments to support key conclusions derived from the study. In this regard, the SnRK1 activity under low nitrogen should be examined (gene transcriptional activation and protein activity are not always correlated). The link between ROS production, RBOH, SnRK1 and gene expression is unclear. In addition, the reviewers indicate that some aspects of the manuscript should be improved, including images quality (phenotypic images such as 1B or 2F), experimental design or procedures [RNA-seq, co-IP or VIGS, which need additional controls], and methodology information (e.g. nitrogen treatment information). In addition, the reviewers suggest that some of the experiments might be improved such as BiFC, in which a co-localization assay might be included (please also double check the use of required controls, as described in this TPC article: https://academic.oup.com/plcell/article/28/5/1002/6098458). The protein/gene nomenclature for SnRK1 might be replace by SnRK1α, perhaps more widely used. Related to this point, information regarding the SnRK1 complex in tomato is almost missing in the text.

Based on these evaluations by experts in the field and our own assessment of your paper, we cannot offer proceed towards publication of this work in TPC. If you decide to complete the story and resubmit to TPC, it will be evaluated as a new submission subject to full assessment by the editorial board, including pre-review and editor selection, and if sent for external review, a new set of reviewers might be chosen.

In addition, we find that the writing suffers from some problems with grammar and awkward phrasing, and in general lacks clarity and conciseness. We strongly encourage the use of a professional editing service to ensure that the work is presented in the best manner and reaches the broadest possible audience. Several editing services have agreed to extend a discount to authors coming to their website via this ASPB page https://aspb.org/aspb-journal-submission-editing-services/

We are sorry that we could not communicate more positive news, but we nevertheless hope that you will find our reviewers' comments helpful. Please contact us if there are ambiguous comments or if you wish to discuss a revision.

If you were to resubmit, it would be important to convince the editors and reviewers that the study adds significant new understanding(s) of mechanisms or processes, or otherwise represents a significant advance in the field, and that the major claims made are fully justified by the data presented. This includes careful consideration and explanation of the various controls used in experiments, the extent and manner of replication, and the statistical analyses used. Sampling methods and the nature of "biological replicates" should be described precisely (i.e. different plants, parts of plants, pooled tissue, independent pools of tissue, sampled at different times, etc.), along with a clear description of and rationale for any statistical analyses conducted. The reader should know exactly what was sampled; what forms the basis of the calculation of any means and other statistical variables and parameters reported. This is also necessary to ensure that proper statistical analysis was conducted.

The Plant Cell now requires authors to complete and submit an author revisions checklist upon submission of a revised manuscript. The aim of the checklist is to aid authors in preparing a high-quality manuscript, facilitate the review and assessment of revised manuscripts, and help to ensure that journal standards are maintained across the board. If your manuscript is accepted, the completed checklist will be published as supplemental material attached to the article online. Please download a copy of the checklist (a fillable PDF form) at this link, for submission with your revised manuscript: https://tpc.msubmit.net/html/Author_Revisions_Checklist.pdf.

Note also that supplemental materials should be restricted to large datasets and tables, presentation of replicates, and validation of reagents, methods, or genotypes. Any data that are used to support the main claims must be in the main manuscript. Supplemental figure legends must indicate what figure in the main manuscript is supported by the supplemental data presented.

We thank you for your interest in and support of The Plant Cell. We hope we will be able to render a more positive decision on future work.

On behalf of the editorial board,

Emilio Gutierrez-Beltran, Board of Reviewing Editors
Siobhán Brady, Senior Editor
Blake Meyers, Editor-in-Chief

The Plant Cell

--- Reviewer comments:

Reviewer #1 (Comments for the Author):

The study by Zhen et al. identifies a SnRK1-dependent mechanism connecting ROS and plant growth under low nitrogen. This is an important study highlighting the connection between SnRK1, ROS machinery, and plant growth. The authors used a robust combination of experiments to support their claims. However, some critical points/experiments need to be done before publishing this study. Further, in many places, the experiments are not sufficiently described in the manuscript. The detailed comments are given below,

Major Comments
1. In the current study, the authors show that the transcript level of tomato SnRK1.1 is induced during LN. As the transcript level of SnRK1 and activity is not clearly correlated, it would be important to analyze what happens to SnRK1 activity under LN in tomatoes. It is an important aspect as the central theme of this study highlights a SnRK1-dependent molecular pathway providing tolerance to low nitrogen conditions to plants. Using a sensitive synthetic reporter of kinase activity, it has been shown in Arabidopsis that SnRK1 activity is downregulated under low nitrogen (https://doi.org/10.1073/pnas.2022942118). Therefore, it is important to clarify what happens to SnRK1 activity under LN in tomatoes. The Arabidopsis SnRK1 activity (activity and T-loop phosphorylation of AKIN10) is downregulated by ROS and cysteines important for this redox-dependent SnRK1 activity seem to be fairly conserved in the plant kingdom (10.1002/1873-3468.12852). As ROS production in LN is directly dependent on SnRK1, monitoring of SnRK1 activity will help to identify important regulatory connections between LN, ROS, and SnRK1.
2. The details of the experimental setup of RNA-seq (control vs LN) need to be given in the manuscript before describing the data. I had to go to the SRA website to check the experimental setup. From the heatmap (Fig. 3B), it seems that the rboh1-1 mutant fails to make the transcriptional reprogramming under LN. It would be better to highlight the critical pathways modulated under LN in WT and how it is affected by the rboh1-1 mutation. This way, the readers can better appreciate the critical differences between the WT and mutant. Also, what is the effect of rboh1-1 mutation on the transcriptional profile in the control condition (WT control vs rboh1-1 control)? Potentially, there is a chance that the transcript profile of WT and rboh1-1 in the control condition is different and this can create biases in the analysis presented in Fig. 3. Therefore, I would recommend authors analyze the RNA-seq by keeping one control (i.e. WT control) and analyze the effect of LN and rboh1-1 mutation (i.e. WT control vs WT LN; WT control vs rboh1-1 control and WT control vs rboh1-1 LN). Also provide the accession number of the project (PRJNA1075204) instead of the accession number of each sample.
3. What happens to the transcript level of RBOH1 under LN? It would be interesting to check whether the transcript level of RBOH1 is affected by the nitrogen status.
4. Tomato SnRK1.1: How many SnRK1 catalytic subunits does tomato possess? A quick search identified another subunit (SlSnRK1.2) with weaker in vitro activity (https://doi.org/10.1016/j.bbapap.2018.05.010). It is better to give an overview of the different subunits and provide the locus ID for clarity. Similarly, the locus ID of all genes studied in this work needs to be provided as part of the manuscript.
5. The phenotypic difference between different genotypes is not clearly appreciable in the panel in many figures. It would be good if the authors could improve this aspect (for example, by also making zoomed versions of the plant images).
6. The results of Co-IP and BiFC are not sufficiently described. This is especially true for the BiFC result. It seems that the interaction is happening predominantly at the plasma membrane (needs to be verified by colocalization with marker proteins). However, when the N-terminal fragment was used, the signal formed discrete puncta. These results and the potential reasons need to be described.
7. The negative control experiments of Y2H are not provided in many places (e.g. Fig. S9A and Fig. 5A).
8. The contrast of the GST pulldown experiment (Fig 5B) is too high. I suggest authors use minimum modifications so that results can be appreciated well.

Minor comments
1. Line 75-77: Specify that you are talking about the Arabidopsis SnRK1 complex which has three catalytic subunits. It is important as different plants possess different copy numbers of catalytic genes. The description of regulatory subunits is not accurate as Arabidopsis contains three beta and one beta-gamma subunit (not the canonical gamma subunit of Snf1/AMPK). Also, it is not clear what are the precise molecular functions of these subunits in plants, so it is better not to specify that (scaffolding function or nucleotide binding).
2. Line 107: When introducing the RBOH, clarify the molecular function of this gene so that readers can appreciate its role in the ROS pathway.
3. Line 158: Specify what you mean by 'heterozygote isolation', it is not clear in the manuscript.

4. Line 168: The chlorophyll content and total biomass is not 'increased' in the SnRK1.1OE line in LN. They also showed a reduction compared to the control condition but maintained higher chlorophyll content and total biomass compared to the WT control. In the same paragraph, the results are clearly described for the N and C estimation. Adopt the same language to accurately describe the results.

Reviewer #2 (Comments for the Author):

Nitrogen (N) is among the most important macronutrients for plant growth and development, and one of the most widely used as fertilizer. Understanding how plants sense and respond to N deficiency is essential for optimizing plant growth and crop yield and reduce the use of chemical fertilizers.

The findings of this manuscript are worthy although I would suggest that the authors address the following points:

Introduction:

1. It would be convenient to differentiate well results obtained in Arabidopsis and obtained in tomato because the results of one species are not always applicable to the other and can lead to confusion.

Material and Methods:

2. The nitrogen concentration used for the low nitrogen condition is perhaps a bit extreme, a very strong stress is seen on the plants. How long was maintained this treatment?, please specify in material an methods section. Have different nitrogen concentrations been analysed? If so, are similar results observed?
3. The number of biological replicates is perhaps a little low. The ideal would be to grow at least 8-10 plants per condition taking into account the variability of each plant in their response to the stress and analyse the phenotype even if not all of them are processed in the end. Number of roots analysed by microscopy should be also higher.
4. Expression in VIGS is missing in Supplementary Figure 9
5. Was it used to determine the different parameters such as chlorophyll, nitrogen, carbon, H2O2, etc., the entire root and the entire aerial part? or certain leaves or leaflets were taken from the aerial part? Please clarify.
6. For the H2O2 accumulation, were only the root tips used or was it also observed above the root tips?
7. For results where there are two variables, such as genotype and treatment (i.e.: Fig. 2D and E), it would be convenient to use a two-way ANOVA analysis.

Results and Discussion:

8. The image of the plants cannot be distinguished very well, perhaps a white background would help.
9. Tomato has different root plasma membrane nitrate transporters: LeNRT1.1, LeNRT1.2, LeNRT2.1, LeNRT2.2 and LeNRT2.3, may authors explain why they analysed only NRT1.1 and NRT2.1, how are the other transporters behaving. In fact, under low nitrate conditions it has been published that NRT2.1 and NRT2.3 are upregulated while NRT1.1 is downregulated (https://doi.org/10.1111/pce.14212). Please discuss.
10. Pg 7, line 188-189: "this low nitrogen-induced upregulation of NRT1.1 and NRT2.1 expression was suppressed in snrk1.1 mutants". Perhaps it is better to say that the induction in the snrk mutants is lower than in the WT, as there is not actually a suppression of the induction (Fig. 1E). Similar comment in lines 210-211 with NADPH activity, there is an increase of NADPH oxidase activity, although lower than in WT (Fig. 1I). The same in pg9, lines 270-271 with rboh1 mutants. In Pg. 10, line 289, instead of "completely offset" better say that H2O2 improves phenotype of mutants.
11. Is RBOH1 the so-called RBOHG? Is it induced under LN stress?. How is the comparison of SlRBOH1 and AtRBOHI?, apparently is in the same subgroup (IV) that AtRBOHF and SlRBOH1 (https://doi.org/10.3389/fpls.2015.00463). Please discuss.
12. It is not clear where came from the 3965 DEGs that were analysed in Fig. 3, which comparisons did the authors made to obtain them?. Which genes are represented in Fig. 3B? all DEGs upregulated in WT are down-regulated in rboh1? And viceversa?. Please explain.
13. It has been shown that TGA1 relies on the oxidation state of Cys residues to mediate the interaction with NPR1 (doi: 10.1105/tpc.012849), to use in the discussion, pg 16, lines 477-481.

Reviewer #3 (Comments for the Author):

STPC2024-RA-00167

SnRK1.1-1 mediated RBOH1 phosphorylation positively regulates ROS production and tolerance to low nitrogen stress in Solanum lycopersicum L.

In this work, the authors studied the role of SnRK1.1 in the responses of tomato plants to low nitrogen supply. ROS production is

controlled by SnRK1 through the activity of the apoplastic NADP oxidase RBOH1. Authors demonstrate in an elegant way the interaction of SnRK1.1 to activate RBOH1 via the phosphorylation of specific serine residues. Furthermore, transcriptomic analysis reveals that nitrogen transport and metabolism genes were influenced by ROS derived from RBOH1 activity under N deficiency. Among them, authors highlight NRT1.1 and NRT2.1.
In my opinion, the main weakness of the manuscript relies in the relation between the increased apoplastic ROS production activated by SnRK1 under N limitation and the induced expression of genes like NRT1.1 and NRT2.1 associated to the N tolerance responses. More data are required to demonstrate the molecular link between the ROS and the increased expression of the selected target genes. Moreover, the involvement of several transcription factors described to be involved in the regulation of the studied nitrate transporters (e.g. TGA, STOP1, NLP7) or related to ROS signaling (HBI1) should be included in the study. Specifically since SnRK1 has been recently reported to control NLP7 transcription factor under N depletion conditions.
A revision of the English language would be recommended (E.g. sentence PP192-194).

Minor issues

Abstract
"Nitrogen is one of the most essential mineral elements..": All essential mineral elements are 'equally' essential.

Introduction
PP 58-63: repetitive. Summarize
PP 70: 'systemic glucose' meaning
PP 90: .".degradation.(Wang.." remove .
PP 244: crucial in the responses to low N
PP 258: transcription, translation

Results
PP 173: Changes in C contents among genotypes are related to differences in photosynthesis or partition in the plant. Clarify
Unify the structure of the figures. E.g. Figures 1C-D and 1E-H

Discussion
Section should be improved by reducing its size and focusing around two-three main topics.

PP 442: Not really demonstrated

Editorial requests:

1. As you see, all the reviewers indicate that although the study is attractive and has novelty, the work needs additional experiments to support key conclusions derived from the study.

Response: We have conducted additional experiments to support the key conclusions of the study and to address the reviewers' comments.

2. In this regard, the SnRK1 activity under low nitrogen should be examined (gene transcriptional activation and protein activity are not always correlated).

Response: We examined SnRK1α1 activity under low nitrogen stress using Anti-AMPK pT172 and Anti-ACC pS79 methods suggested by the reviewer. As shown in Fig. 1B and C, SnRK1α1 activity increased in WT plants and was further upregulated in *SnRK1α1* overexpressing lines under low nitrogen stress. However, mutation of SnRK1α1 suppressed its activity (Fig. 1C). These results suggest that both *SnRK1α1* gene transcriptional activation and protein activity are increased under low nitrogen stress.

3. The link between ROS production, RBOH, SnRK1 and gene expression is unclear.

Response: We have conducted additional experiments and found that SnRK1α1-mediated RBOH1 phosphorylation positively regulates ROS production. In turn, RBOH1-dependent ROS enhance the activity of transcription factor TGA4 through oxidative modification. TGA4 then binds to the promoters of *NRT1.1* and *NRT2.1*, upregulating their expression under low nitrogen stress. This clarifies the relationship between ROS production, RBOH, SnRK1α1, and gene expression. A detailed model summarizing these findings is presented in Fig. 9.

4. In addition, the reviewers indicate that some aspects of the manuscript should be improved, including images quality (phenotypic images such as 1B or 2F), experimental design or procedures [RNA-seq, co-IP or VIGS, which need additional controls], and methodology information (e.g. nitrogen treatment information).

Response: We have improved image quality and experimental design, and have provided detailed methodological information as suggested by the reviewers. Please

see below for specific responses to their comments.

5. In addition, the reviewers suggest that some of the experiments might be improved such as BiFC, in which a co-localization assay might be included (please also double check the use of required controls, as described in this TPC article: https://academic.oup.com/plcell/article/28/5/1002/6098458).

Response: We have improved the experiments based on the reviewers' suggestions. Please see below for specific responses to their comments.

6. The protein/gene nomenclature for SnRK1 might be replace by SnRK1α, perhaps more widely used. Related to this point, information regarding the SnRK1 complex in tomato is almost missing in the text.

Response: SnRK1 is now replaced by SnRK1α1 in this article. In tomatoes, SnRK1 forms a heterotrimeric complex with two catalytic α-subunits, SlSnRK1α1 and SlSnRK1α2. SlSnRK1α1 is highly homologous to AtSnRK1α1, as shown in Supplementary Fig. 1 (Su and Devarenne, 2018). The complex also includes four substrate-interacting β-subunits (SlGal83, SlSip1, SlTau1, and SlTau2) and a regulatory γ-subunit (SlSnf4) (Bradford et al., 2003; Avila et al., 2012). The phylogenetic divergence and lower kinase activity of SlSnRK1α2 compared to SlSnRK1α1 suggest distinct in vivo roles for each kinase (Su and Devarenne, 2018). Information on tomato and Arabidopsis SnRK1 complexes is provided in Supplementary Fig. 1.

Reviewer #1 (Comments for the Author)

The study by Zhen et al. identifies a SnRK1-dependent mechanism connecting ROS and plant growth under low nitrogen. This is an important study highlighting the connection between SnRK1, ROS machinery, and plant growth. The authors used a robust combination of experiments to support their claims. However, some critical points/experiments need to be done before publishing this study. Further, in many places, the experiments are not sufficiently described in the manuscript. The detailed comments are given below.

Response: Many thanks for your positive comments and suggestions. We have

included additional data that were not present in the original version and completed further experiments in response to your feedback. We have also provided detailed methodological information as suggested. Our responses to your comments are listed below, point-by-point.

Major Comments

1. In the current study, the authors show that the transcript level of tomato SnRK1.1 is induced during LN. As the transcript level of SnRK1 and activity is not clearly correlated, it would be important to analyze what happens to SnRK1 activity under LN in tomatoes. It is an important aspect as the central theme of this study highlights a SnRK1-dependent molecular pathway providing tolerance to low nitrogen conditions to plants. Using a sensitive synthetic reporter of kinase activity, it has been shown in Arabidopsis that SnRK1 activity is downregulated under low nitrogen (https://doi.org/10.1073/pnas.2022942118). Therefore, it is important to clarify what happens to SnRK1 activity under LN in tomatoes. The Arabidopsis SnRK1 activity (activity and T-loop phosphorylation of AKIN10) is downregulated by ROS and cysteines important for this redox-dependent SnRK1 activity seem to be fairly conserved in the plant kingdom (10.1002/1873-3468.12852). As ROS production in LN is directly dependent on SnRK1, monitoring of SnRK1 activity will help to identify important regulatory connections between LN, ROS, and SnRK1.

Response: Thank you for your suggestion on providing further evidence of SnRK1α1 protein activity in tomato under low nitrogen stress. We appreciate the references you provided, which were crucial for our experimental protocol. Using similar designs, we found that SnRK1α1 activity increased in WT plants and was further upregulated in *SnRK1α1* overexpressing lines under low nitrogen stress (Fig. 1B, C). Conversely, mutation of *SnRK1α1* suppressed its activity (Fig. 1C). These results indicate that both SnRK1α1 gene transcriptional activation and protein activity are elevated under low nitrogen stress.

As noted, Arabidopsis SnRK1α1 activity is downregulated by ROS, with cysteines important for this redox-dependent activity being conserved across the plant kingdom (Wurzinger et al., 2017). ROS-mediated post-translational modifications on cysteine

residues can either promote or suppress protein activity (Waszczak et al., 2015; Bi et al., 2022; Zhou et al., 2023). While Wurzinger et al. found that H_2O_2 suppresses SnRK1α1 activity in Arabidopsis, our study shows that RBOH1-dependent ROS increase tomato TGA4 transcriptional activity through oxidative modification at residue Cys-334. These results suggest that ROS-mediated oxidative modifications of different protein cysteine residues can have varying effects. The upregulation of RBOH1-dependent ROS by SnRK1α1 and the downregulation of SnRK1α1 activity by ROS represent plant stress response strategies. SnRK1α1-mediated RBOH1 phosphorylation positively regulates ROS production, which in turn activates TGA4 and other transcription factors to induce the expression of nitrogen absorption and transport genes, such as *NRT1.1* and *NRT2.1*, under low nitrogen stress. Excessive ROS, however, can affect SnRK1α1 activity, creating a negative feedback loop to maintain redox balance under stress. We have added these discussions to the revised manuscript (Discussion section, lines 501-509) and summarized a detailed model in Fig. 9.

2. The details of the experimental setup of RNA-seq (control vs LN) need to be given in the manuscript before describing the data. I had to go to the SRA website to check the experimental setup. From the heatmap (Fig. 3B), it seems that the rboh1-1 mutant fails to make the transcriptional reprogramming under LN. It would be better to highlight the critical pathways modulated under LN in WT and how it is affected by the rboh1-1 mutation. This way, the readers can better appreciate the critical differences between the WT and mutant. Also, what is the effect of rboh1-1 mutation on the transcriptional profile in the control condition (WT control vs rboh1-1 control)? Potentially, there is a chance that the transcript profile of WT and rboh1-1 in the control condition is different and this can create biases in the analysis presented in Fig. 3. Therefore, I would recommend authors analyze the RNA-seq by keeping one control (i.e. WT control) and analyze the effect of LN and rboh1-1 mutation (i.e. WT control vs WT LN; WT control vs rboh1-1 control and WT control vs rboh1-1 LN). Also provide the accession number of the project (PRJNA1075204) instead of the

accession number of each sample.

Response: Thank you very much for your detailed suggestion. We have elaborated on the RNA-seq experimental setup in the manuscript (Lines 255-257), providing a more detailed description before presenting the data. In Fig. 3 and Supplementary Fig. 7, we included multiple comparisons in the transcriptome analysis (LN WT vs Control WT; LN *rboh1-1* vs Control *rboh1-1*; LN *rboh1-1* vs LN WT; Control *rboh1-1* vs Control WT) as you advised (Supplementary Data Set 1-8). In the roots of WT plants, 3550 genes exhibited significant upregulation following low nitrogen treatment, while in *rboh1-1* mutants, only 1127 genes showed such an increase (Fig. 3A, Supplementary Data Set 1-2). Conversely, 811 genes were downregulated in WT roots under low nitrogen stress, compared to a substantial 2853 genes in *rboh1* mutants (Fig. 3A, Supplementary Data Set 1-2). Furthermore, we compared LN *rboh1-1* with LN WT and found that 20642 genes had no significant difference between the two lines, 1123 genes were expressed significantly higher in *rboh1-1* mutants than that of WT, however, there are 3670 genes in *rboh1-1* mutants were expressed significantly lower than that of WT under low nitrogen stress (Fig. 3B, Supplementary Fig. 7A and Supplementary Data Set 3). These results indicate that the loss function of RBOH1 impacts the expression pattern of genes associated with low nitrogen response in tomato roots. In addition, we also compared Control *rboh1-1* and Control WT and found that 1804 genes in the *rboh1-1* mutant had significantly higher FPKM than WT, 239 genes had significantly lower FPKM, and 23392 genes had no significant differences in FPKM under control conditions (Supplementary Fig. 7). We conducted GO enrichment analysis and KEGG analysis on 2043 differentially expressed genes (*DEGs*) in the comparison between Control *rboh1-1* and Control WT. Most of these genes are associated with the plasma membrane, cell wall, and hydrolase activity, and are involved in the synthesis of various secondary metabolites such as flavonoids. This finding is consistent with previous studies, which have shown that ROS can cause lipid peroxidation reactions with phospholipids and membrane receptor proteins, affecting the integrity and fluidity of the membrane system (Mhamdi et al., 2010);

The positive effects of ROS on plants have also been observed in the regulation of cell wall formation (Potikha et al., 1999). Additionally, RBOH1-dependent ROS can promote the degradation of the pollen tapetum (Yan et al., 2020). Furthermore, flavonoid accumulation is induced by various ROS-inducing developmental signals(Chapman et al., 2019) and the biosynthesis of anthocyanins is also influenced by ROS (Zhang et al., 2024). These results suggest that ROS also plays a significant role in plant growth, which is consistent with our transcriptomic data. We have included the analysis of these transcriptomic results in the result (Lines 257-298).

Additionally, we have included the project accession number PRJNA1075204 (SRR30233841 to SRR30233852) (Lines 1016-1017) in the Data Availability section of the manuscript, as suggestion.

3. What happens to the transcript level of RBOH1 under LN? It would be interesting to check whether the transcript level of RBOH1 is affected by the nitrogen status.

Response: Following your suggestion, we detected the transcript level of *RBOH1* gene and found that its expression was induced 3.2-fold on the first day of low nitrogen treatment and maintained a consistent and significant increase from days 1 to 3 compared to the control treatment (Fig. 2B) (Lines 229-231). This indicates that the transcript level of RBOH1 is indeed affected by low nitrogen.

4. Tomato SnRK1.1: How many SnRK1 catalytic subunits does tomato possess? A quick search identified another subunit (SlSnRK1.2) with weaker in vitro activity (https://doi.org/10.1016/j.bbapap.2018.05.010). It is better to give an overview of the different subunits and provide the locus ID for clarity. Similarly, the locus ID of all genes studied in this work needs to be provided as part of the manuscript.

Response: In Supplementary Fig. 1, we have included an evolutionary tree analysis of different SnRK1 subunits in tomato and *Arabidopsis thaliana*, along with site IDs for easier comparison and analysis, as per your suggestions. SnRK1 forms a heterotrimeric complex in tomatoes, consisting of two catalytic α-subunits:

SlSnRK1α1 (Solyc02g067030) and SlSnRK1α2 (Solyc03g115700), SlSnRK1α1 is highly homologous to AtSnRK1α1 (AT3G01090) as shown in Supplementary Fig.1 (Su and Devarenne, 2018), four substrate-interacting β-subunits (SlGal83, SlSip1, SlTau1, and SlTau2), and a regulatory γ-subunit (SlSnf4) in tomato (Bradford et al., 2003; Avila et al., 2012). The phylogenetic divergence and lower kinase activity of SnRK1α2 compared to SlSnRK1α1 suggest that each kinase may have distinct roles in vivo (Su and Devarenne, 2018). An overview of the different subunits and the locus ID of the genes studied in this work is provided in Supplementary Fig. 1.

5. The phenotypic difference between different genotypes is not clearly appreciable in the panel in many figures. It would be good if the authors could improve this aspect (for example, by also making zoomed versions of the plant images).

Response: Based on your suggestions, we have improved the figure settings by displaying multiple plants from the same line rather than just a single plant. This approach has enhanced the comparison of plant groups, resulting in a clearer depiction of phenotypic differences (Fig. 1D, Fig. 2H, Fig. 4A, Fig. 7A, etc.).

6. The results of Co-IP and BiFC are not sufficiently described. This is especially true for the BiFC result. It seems that the interaction is happening predominantly at the plasma membrane (needs to be verified by colocalization with marker proteins). However, when the N-terminal fragment was used, the signal formed discrete puncta. These results and the potential reasons need to be described.

Response: Based on your advice, we have provided a detailed description of the results from the Co-IP (Lines 344-346) and BiFC (Lines 350-354) experiments in the manuscript. To assess the membrane localization of the interaction between RBOH1/N-nYFP and cYFP-SnRK1α1, we used the mCherry-FLS2 fusion protein as a plasma membrane marker (Ma et al., 2022; Wang et al., 2022). As shown in Figure 5E, SnRK1α1 was found to interact with either full length or N-terminal fragment of RBOH1 on the plasma membrane, but it did not interact with C-terminal fragment of RBOH1.

7. The negative control experiments of Y2H are not provided in many places (e.g. Fig. S9A and Fig. 5A).

Response: We have included the negative control experiments for Y2H in the revised version (Fig. 5A, Supplementary Fig. 12, and Supplementary Fig. 14).

8. The contrast of the GST pulldown experiment (Fig 5B) is too high. I suggest authors use minimum modifications so that results can be appreciated well.

Response: We have decreased the contrast in the GST pulldown experiment and other similar experiments in the revised version.

Minor comments1. Line 75-77: Specify that you are talking about the Arabidopsis SnRK1 complex which has three catalytic subunits. It is important as different plants possess different copy numbers of catalytic genes. The description of regulatory subunits is not accurate as Arabidopsis contains three beta and one beta-gamma subunit (not the canonical gamma subunit of Snf1/AMPK). Also, it is not clear what are the precise molecular functions of these subunits in plants, so it is better not to specify that (scaffolding function or nucleotide binding).

Response: Thank you very much for your advice. Based on your comments, we have revised the corresponding part (Lines 78-81). The Arabidopsis SnRK1 complex comprises three α catalytic subunits (SnRK1α1, SnRK1α2, and SnRK1α3) and two regulatory subunits, β-subunits and one βγ- subunit (Emanuelle et al., 2015; Rodriguez et al., 2019; Wang et al., 2020). We have included an evolutionary tree analysis of different SnRK1 subunits in tomato and *Arabidopsis thaliana*, along with site IDs for easier comparison and analysis (Supplementary Fig 1). SnRK1 forms a heterotrimeric complex in tomatoes, consisting of two catalytic α-subunits: SlSnRK1α1 and SlSnRK1α2, SlSnRK1α1 is highly homologous to AtSnRK1α1 as shown in Supplementary Fig.1 (Su and Devarenne, 2018), four substrate-interacting β-subunits (SlGal83, SlSip1, SlTau1, and SlTau2), and a regulatory γ-subunit (SlSnf4)

in tomato (Bradford et al., 2003; Avila et al., 2012).The details about the SnRK1 complex are provided in the Supplementary Fig.1. We have also removed the description of the precise molecular functions of these subunits in plants (such as scaffolding function or nucleotide binding) as suggested.

2. Line 107: When introducing the RBOH, clarify the molecular function of this gene so that readers can appreciate its role in the ROS pathway.

Response: We have clarified the molecular function of RBOHs as suggested (Lines 101-114). We compared the RBOH family in Arabidopsis with that in tomato, emphasizing the roles of RBOHD and RBOHF in roots, as well as RBOHF's function in stress responses. Specifically, NADPH oxidase/respiratory burst oxidase homolog (RBOH) proteins generate localized bursts of ROS, crucial for regulating growth, development, and stress responses (Mittler et al., 2022). The Arabidopsis RBOH family includes 10 members (RBOHA to RBOHJ), each with distinct roles in development and stress responses (Sagi and Fluhr, 2006). RBOHD and RBOHF are highly expressed in root tissues and are important for root growth and environmental responses (Li et al., 2015a; Chapman et al., 2019). RBOHF is particularly significant in abiotic stress responses, such as ABA signaling and salt stress tolerance (Kwak et al., 2003; Ma et al., 2012; Jiang et al., 2013). In tomato, the RBOH family has eight members (RBOH1, RBOHA-F, and RBOHH), with RBOH1 (Solyc08g081690) being the closest to Arabidopsis RBOHF. Tomato RBOH1 is highly expressed in roots (Yan et al., 2020) and plays a role in heat and oxidative stress responses through H_2O_2 signaling (Zhou et al., 2014a).

3. Line 158: Specify what you mean by 'heterozygote isolation', it is not clear in the manuscript.

Response: During the creation and cultivation of *snrk1α1* homozygous mutants, we observed that SnRK1α1 influences flower and fruit development, leading to abortion (Supplementary Fig. 2C). Therefore, we screened for *snrk1α1* homozygous mutant

seedlings from *snrk1α1* heterozygotes in this study. We have described the screening process in the Results section (Lines 175-178).

4. Line 168: The chlorophyll content and total biomass is not 'increased' in the SnRK1.1 OE line in LN. They also showed a reduction compared to the control condition but maintained higher chlorophyll content and total biomass compared to the WT control. In the same paragraph, the results are clearly described for the N and C estimation. Adopt the same language to accurately describe the results.

Response: Thanks to your advice, we have given a more accurate description of the corresponding results in the manuscript based on your comments. As shown in Fig. 1E, both the chlorophyll content and total biomass of *snrk1α1* plants were significantly lower than those of WT plants after low nitrogen conditions. In contrast, the chlorophyll content and total biomass were significantly higher in *SnRK1α1* OE plants compared with those of WT seedlings under low nitrogen stress (Fig. 1E) (Lines 186-189). As shown in Fig. 1F, under sufficient nitrogen conditions, there were no marked differences in nitrogen and carbon contents among the roots of WT, *snrk1α1* mutants, and *SnRK1α1* OE plants. However, with three weeks of low nitrogen treatment, the nitrogen and carbon contents in the roots of *snrk1α1* mutants significantly decreased compared to that in the roots of WT. Although the nitrogen and carbon contents of the roots of *SnRK1α1* OE plants also diminished under low nitrogen treatment, they remained higher than those in WT (Fig. 1F) (Lines 190-195).

Reviewer #2 (Comments for the Author): Nitrogen (N) is among the most important macronutrients for plant growth and development, and one of the most widely used as fertilizer. Understanding how plants sense and respond to N deficiency is essential for optimizing plant growth and crop yield and reduce the use of chemical fertilizers. The findings of this manuscript are worthy although I would suggest that the authors address the following points:

Response: Thank you for your positive feedback and suggestions. We have included

additional data that was missing in the original version and conducted further experiments based on your comments. Our detailed responses to each of your points are listed below.

Introduction:

1. It would be convenient to differentiate well results obtained in Arabidopsis and obtained in tomato because the results of one species are not always applicable to the other and can lead to confusion.

Response: Thank you for your suggestion. To provide readers with a better understanding of the molecular functions of SnRK1 and RBOHs, we have compared their functions across different plants, including Arabidopsis and tomato.

Material and Methods:

2. The nitrogen concentration used for the low nitrogen condition is perhaps a bit extreme, a very strong stress is seen on the plants. How long was maintained this treatment? please specify in material and methods section. Have different nitrogen concentrations been analysed? If so, are similar results observed?

Response: The low nitrogen treatment was maintained for three weeks, as specified in Materials and Methods section based on your comments (Line 582). To determine the optimal nitrate concentration for low nitrogen treatment, we exposed wild-type (WT) plants to four different nitrate concentrations, changing the nutrient solution every two days. As shown in the figure below, 10 mM NO_3^- was used as a control. Compared to the control, plants treated with 1 mM NO_3^- exhibited a significant decrease in biomass, although chlorophyll content remained unchanged. Treatment with 0.01 mM NO_3^- resulted in significant reductions in both chlorophyll content and biomass, but also caused considerable damage to the plants, which hindered line comparisons. In contrast, treatment with 0.1 mM NO_3^- led to noticeable differences in both chlorophyll content and biomass compared to the control, with relatively less damage to the plants than at 0.01 mM NO_3^-. Therefore, we selected 0.1 mM NO_3^- as

the optimal concentration for low nitrogen treatment in this study.

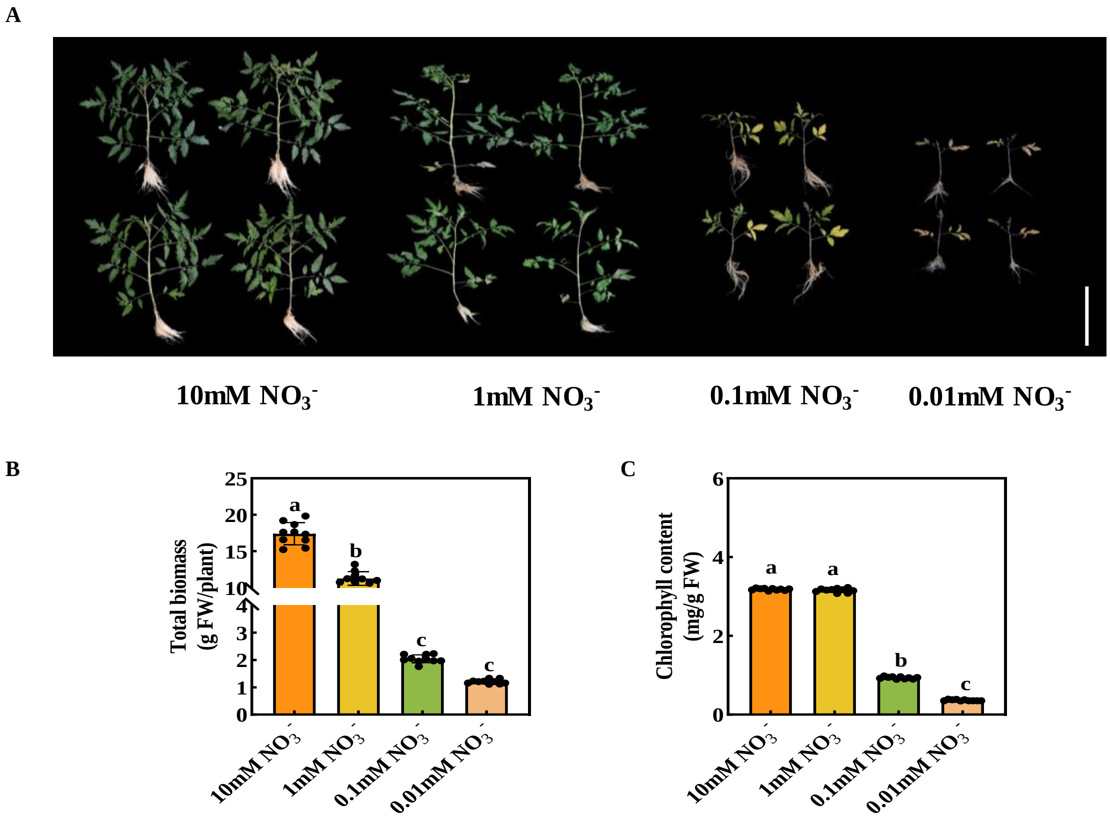

Figure 1 The effect of different NO_3^- concentrations on tomato seedling growth

3. The number of biological replicates is perhaps a little low. The ideal would be to grow at least 8-10 plants per condition taking into account the variability of each plant in their response to the stress and analyse the phenotype even if not all of them are processed in the end. Number of roots analysed by microscopy should be also higher.

Response: Based on your comments, we re-conducted the experiments using 10 biological replicates for each strain and obtained consistent results. The newly obtained results are presented in the revised manuscript (Fig. 1D-E, Fig. 2H-I, Fig. 4A-B, Fig. 7A-B, Supplementary Fig. 9A-C, Supplementary Fig. 10A-B). Additionally, we included images of 10 roots analyzed by microscopy to facilitate comparison of ROS-induced differences among strains.

4. Expression in VIGS is missing in Supplementary Figure 9

Response: Expression data for VIGS is provided in Supplementary Fig. 11 in the revised version.

5. Was it used to determine the different parameters such as chlorophyll, nitrogen, carbon, H2O2, etc., the entire root and the entire aerial part? or certain leaves or leaflets were taken from the aerial part? Please clarify.

Response: Normal functional leaves were used for chlorophyll extraction, the entire aerial parts and roots for carbon and nitrogen content determination, and the entire roots for H_2O_2 determination. This information has been added to the Materials and Methods section (Lines 639, 648-648, and 654) as per your suggestion.

6. For the H2O2 accumulation, were only the root tips used or was it also observed above the root tips?

Response: As shown in Fig. 1H and 1I, H_2O_2 accumulation is observed both in the root tips and above them, with higher accumulation near the root tip under low nitrogen conditions. We detected ROS accumulation in root tips using DAB, DCF, and $CeCl_3$ stainings and quantitatively assessed H_2O_2 content and NADPH oxidase activity in the entire roots. The trends in H_2O_2 accumulation are consistent between root tips and the entire roots.

7. For results where there are two variables, such as genotype and treatment (i.e.: Fig. 2D and E), it would be convenient to use a two-way ANOVA analysis.

Response: Thanks for your suggestion, in the revised version, we have carried out a two-way ANOVA analysis of the corresponding data (Fig. 1G, Fig. 1K, Fig. 1L, Fig. 2F, Fig. 2G, Fig. 3E, Fig. 4D, Fig. 4H, Fig. 4I, Fig. 6D, Fig. 6E, Fig. 7C, Fig. 8J, Supplementary Fig. 10B, Supplementary Fig. 10C, Supplementary Fig. 10D, Supplementary Fig. 18, Supplementary Fig. 20, Supplementary Fig. 21).

Results and Discussion:

8. The image of the plants cannot be distinguished very well, perhaps a white background would help.

Response: After replacing the background with white, we found that the contrast of leaf yellowing phenotypes between different lines was not very apparent under low nitrogen treatment. Therefore, we used a black background to enhance contrast. Additionally, we included multiple plants from the same line in the diagram rather than just one plant, which improved the comparison effect of plant groups and made it easier to distinguish between plants from different lines (Fig. 1D, Fig. 2H, Fig. 4A, Fig. 7A, Supplementary Fig. 9, Supplementary Fig. 10).

9. Tomato has different root plasma membrane nitrate transporters: LeNRT1.1, LeNRT1.2, LeNRT2.1, LeNRT2.2 and LeNRT2.3, may authors explain why they analysed only NRT1.1 and NRT2.1, how are the other transporters behaving. In fact, under low nitrate conditions it has been published that NRT2.1 and NRT2.3 are upregulated while NRT1.1 is downregulated (https://doi.org/10.1111/pce.14212). Please discuss.

Response: Through transcriptome analysis, we found significant differences in the expression of *NRT1.1* and *NRT2.1* between the *rboh1-1* mutants and wild type (WT) under low nitrogen conditions. Their expression levels in roots were notably higher than those of other *NRTs*, leading us to select *NRT1.1* and *NRT2.1* as representative genes for further study (Supplementary Fig. 8). Previous studies involved conditions of high phosphorus and high nitrogen, high phosphorus and low nitrogen, and low phosphorus and low nitrogen (Marro et al., 2021), differing from the low nitrogen conditions in our study. Plants have evolved complex signal regulatory networks to integrate signals from different nutrient elements (Hu et al., 2019; Zhang et al., 2021) , so *NRT1.1* expression might also be influenced by phosphorus levels, leading to observed differences.

10. Pg 7, line 188-189: "this low nitrogen-induced upregulation of NRT1.1 and

NRT2.1 expression was suppressed in snrk1.1 mutants". Perhaps it is better to say that the induction in the snrk mutants is lower than in the WT, as there is not actually a suppression of the induction (Fig. 1E). Similar comment in lines 210-211 with NADPH activity, there is an increase of NADPH oxidase activity, although lower than in WT (Fig. 1I). The same in pg9, lines 270-271 with rboh1 mutants. In Pg. 10, line 289, instead of "completely offset" better say that H2O2 improves phenotype of mutants.

Response: Thanks for your revision suggestion; we have updated these sentences in the new manuscript.

11. Is RBOH1 the so-called RBOHG? Is it induced under LN stress?. How is the comparison of SlRBOH1 and AtRBOHI?, apparently is in the same subgroup (IV) that AtRBOHF and SlRBOH1 (https://doi.org/10.3389/fpls.2015.00463). Please discuss.

Response: As you mentioned, AtRBOHF and SlRBOH1 belong to the same subgroup (IV) (Li et al., 2015b). We have compared the functions of these RBOH homologs across different plants in the Introduction (Lines 101-114). Following your suggestion, we detected *RBOH1* expression during low nitrogen treatment, observing a 3.2-fold increase at 1 day of treatment, which remained consistently and significantly higher from days 1 to 3 compared to the control (Fig. 2B) (Lines 229-231).

12. It is not clear where came from the 3965 DEGs that were analysed in Fig. 3, which comparisons did the authors made to obtain them? Which genes are represented in Fig. 3B? all DEGs upregulated in WT are down-regulated in rboh1? And viceversa? Please explain.

Response: Based on your comments and those of Reviewer #1, we have reanalyzed the data presented in Fig. 3 and clarified the results in the new version. We compared LN *rboh1-1* with LN WT and found that 20642 genes had no significant difference between the two lines, 1123 genes were expressed significantly higher in *rboh1-1*

mutants than that of WT, however, there are 3670 genes in *rboh1-1* mutants were expressed significantly lower than that of WT under low nitrogen stress (Fig. 3B, Supplementary Fig. 7 and Supplementary Data Set 3). So, there are 4793 *DEGs* between LN *rboh1-1* and LN WT, as shown in Figure 3B. We then performed Gene Ontology (GO) functional analysis and Kyoto Encyclopedia of Genes and Genomes (KEGG) pathway analysis on these 3670 *DEGs*, which were expressed significantly lower in *rboh1-1* mutants than that of WT under low nitrogen stress (Fig. 3C-D). Our findings indicate that the absence of RBOH1 under low nitrogen conditions affects carbon metabolism, membrane transport processes (including six nitrate transporters), nitrogen metabolism, various amino acid metabolic processes, and amino acid biosynthesis.

13. It has been shown that TGA1 relies on the oxidation state of Cys residues to mediate the interaction with NPR1 (doi: 10.1105/tpc.012849), to use in the discussion, pg 16, lines 477-481.

Response: Thank you for providing the literature, which has been valuable for the experimental design in our supplementary experiments. We have incorporated this information into the discussion section of the revised manuscript (Lines 552-558). Although TGA1 and TGA4 are highly homologous, our experiments have shown that tomato TGA1 is insensitive to H_2O_2 (Supplementary Fig. 16), which differs from Arabidopsis. However, tomato TGA4 can be oxidized by H_2O_2 (Fig. 8). TGA1 lacks the Cys-334 residue found in TGA4 (Supplementary Fig. 23), which may explain TGA1's insensitivity to H_2O_2.

Reviewer #3 (Comments for the Author):

STPC2024-RA-00167

SnRK1.1-1 mediated RBOH1 phosphorylation positively regulates ROS production and tolerance to low nitrogen stress in *Solanum lycopersicum* L.

In this work, the authors studied the role of SnRK1.1 in the responses of tomato plants

to low nitrogen supply. ROS production is controlled by SnRK1 through the activity of the apoplastic NADP oxidase RBOH1. Authors demonstrate in an elegant way the interaction of SnRK1.1 to activate RBOH1 via the phosphorylation of specific serine residues. Furthermore, transcriptomic analysis reveals that nitrogen transport and metabolism genes were influenced by ROS derived from RBOH1 activity under N deficiency. Among them, authors highlight NRT1.1 and NRT2.1.

In my opinion, the main weakness of the manuscript relies in the relation between the increased apoplastic ROS production activated by SnRK1 under N limitation and the induced expression of genes like NRT1.1 and NRT2.1 associated to the N tolerance responses. More data are required to demonstrate the molecular link between the ROS and the increased expression of the selected target genes. Moreover, the involvement of several transcription factors described to be involved in the regulation of the studied nitrate transporters (e.g. TGA, STOP1, NLP7) or related to ROS signaling (HBI1) should be included in the study. Specifically, since SnRK1 has been recently reported to control NLP7 transcription factor under N depletion conditions.

A revision of the English language would be recommended (E.g. sentence PP192-194).

Response: Thank you for your valuable suggestions regarding our future research direction. We have incorporated additional data and conducted further experiments in response to your comments. Here is an outline of our responses to each point you raised. In response to your suggestions, we initially screened the sensitivity of various transcription factors related to nitrate transporter gene expression (e.g., TGA1, TGA4, STOP1a, STOP1b, TCP20) to H_2O_2 oxidation. We verified that TGA4 and STOP1a are particularly sensitive to H_2O_2 oxidation. Additionally, we found that *NRT1.1* and *NRT2.1* can be transcriptionally regulated by TGA4, but not by STOP1a in tomato. Using LC-MS, we identified the oxidation site Cys-334 on TGA4 and conducted a series of experiments focusing on this site. Our results confirmed that H_2O_2-mediated oxidation of TGA4 enhances its binding to the promoters of *NRT1.1* and *NRT2.1* under low nitrogen conditions, thereby influencing the expression of these genes.

Detailed experimental methods and results are provided in the revised manuscript (Fig.8 and Supplementary Fig. 16-23). In summary, we have found that SnRK1α-mediated RBOH1 phosphorylation positively regulates ROS production. Meanwhile, RBOH1-dependent ROS promote the activity of transcription factor TGA4 by oxidative modification. TGA4 binds to the promoters of *NRT1.1* and *NRT1.2* and upregulates their expression under low nitrogen stress. Thus, the relationship between ROS production, RBOH, SnRK1 and gene expression is clear now. We have summarized a detailed model in Fig. 9.

Minor issues

Abstract

"Nitrogen is one of the most essential mineral elements.": All essential mineral elements are 'equally' essential.

Response: We have revised the description in the revised manuscript (Line 20).

Introduction

PP 58-63: repetitive. Summarize

Response: We have summarized the introduction in the new version as your suggestion.

PP 70: 'systemic glucose' meaning

Response: We have revised the description in the new version, changing 'systemic glucose' to 'glucose' for greater accuracy (Line 74).

PP 90: .".degradation.(Wang.." remove .

Response: We have removed it in the revised manuscript (Line 93).

PP 244: crucial in the responses to low N

Response: We have revised it in the revised manuscript (Line 251-252).

PP 258: transcription, translation

Response: We have revised it in the revised manuscript (Line 270).

Results

PP 173: Changes in C contents among genotypes are related to differences in photosynthesis or partition in the plant. Clarify

Response: Maintaining a balance between carbon and nitrogen is essential for proper plant development. Nitrogen deficiency impairs carbon absorption, affects photosynthesis, and reduces the overall carbon content of the plant (Chen et al., 2016).

Unify the structure of the figures. E.g. Figures 1C-D and 1E-H

Response: We have unified the structure of the figures (Fig. 1E-F, Fig. 1G, Fig. 1K-L; Fig. 2F-G, Fig. 2K-L) in the new version as your suggestion.

Discussion

Section should be improved by reducing its size and focusing around two-three main topics.

Response: Thanks for your advice, we have improved the section and focusing around three topics: 1. SnRK1α1 enhances plant tolerance to low nitrogen stress through multiple mechanisms and promotes the production of ROS in tomato roots under low nitrogen conditions. 2. Under low nitrogen conditions, SnRK1α1 in tomato roots induces ROS production by phosphorylating RBOH1. These RBOH1-induced ROS can affect the expression of several nitrate transporters (such as *NRT1.1* and *NRT2.1*), thereby promoting nitrogen uptake. 3. RBOH1-induced ROS facilitate the binding of the transcription factor TGA4 to the promoters of *NRT1.1* and *NRT2.1* through oxidative modifications, thereby enhancing the expression of *NRT1.1* and *NRT2.1* genes in tomato roots under low nitrogen conditions (Lines 461-573).

PP 442: Not really demonstrated

Response: In the previous version, we lacked sufficient evidence to establish the relationship between ROS and the expression of *NRT1.1* and *NRT2.1* genes. In the new version, we have conducted additional experiments. As previously mentioned, under low nitrogen conditions, ROS modify TGA4 through oxidative post-translational modifications, enhancing its binding to the promoters of *NRT1.1* and *NRT2.1*, which in turn promotes their expression.

Reference

Avila, J., Gregory, O.G., Su, D.Y., Deeter, T.A., Chen, S.X., Silva-Sanchez, C., Xu, S.L., Martin, G.B., and Devarenne, T.P. (2012). The β-Subunit of the SnRK1 Complex Is Phosphorylated by the Plant Cell Death Suppressor Adi3. Plant Physiol **159,** 1277-1290. http://dx.doi.org/10.1104/pp.112.198432

Bi, G.Z., Hu, M., Fu, L., Zhang, X.J., Zuo, J.R., Li, J.Y., Yang, J., and Zhou, J.M. (2022). The cytosolic thiol peroxidase PRXIIB is an intracellular sensor for H_2O_2 that regulates plant immunity through a redox relay. Nature Plants **8,** 1160–1175. http://dx.doi.org/10.1038/s41477-022-01252-5

Bradford, K.J., Downie, A.B., Gee, O.H., Alvarado, V., Yang, H., and Dahal, P. (2003). Abscisic acid and gibberellin differentially regulate expression of genes of the SNF1-related kinase complex in tomato seeds. Plant Physiol **132,** 1560-1576. http://dx.doi.org/10.1104/pp.102.019141

Chapman, J.M., Muhlemann, J.K., Gayornba, S.R., and Muday, G.K. (2019). RBOH-Dependent ROS Synthesis and ROS Scavenging by Plant Specialized Metabolites To Modulate Plant Development and Stress Responses. Chemical Research in Toxicology **32,** 370-396. http://dx.doi.org/10.1021/acs.chemrestox.9b00028

Chen, X.B., Yao, Q.F., Gao, X.H., Jiang, C.F., Harberd, N.P., and Fu, X.D. (2016). Shoot-to-Root Mobile Transcription Factor HY5 Coordinates Plant Carbon and Nitrogen Acquisition. Current Biology **26,** 640-646. http://dx.doi.org/10.1016/j.cub.2015.12.066

Emanuelle, S., Hossain, M.I., Moller, I.E., Pedersen, H.L., van de Meene, A.M.L., Doblin, M.S., Koay, A., Oakhill, J.S., Scott, J.W., Willats, W.G.T., Kemp, B.E., Bacic, A., Gooley, P.R., and Stapleton, D.I. (2015). SnRK1 from Arabidopsis thaliana is an atypical AMPK. Plant Journal **82,** 183-192. http://dx.doi.org/10.1111/tpj.12813

Hu, B., Jiang, Z.M., Wang, W., Qiu, Y.H., Zhang, Z.H., Liu, Y.Q., Li, A.F., Gao, X.K., Liu, L.C., Qian, Y.W., Huang, X.H., Yu, F.F., Kang, S., Wang, Y.Q., Xie, J.P., Cao, S.Y., Zhang, L.H., Wang, Y.C., Xie, Q., Kopriva, S., and Chu, C.C. (2019). Nitrate-NRT1.1B-SPX4 cascade integrates nitrogen and phosphorus signalling networks in plants. Nature Plants **5,** 401-413. http://dx.doi.org/10.1038/s41477-019-0384-1

Jiang, C.F., Belfield, E.J., Mithani, A., Visscher, A., Ragoussis, J., Mott, R., Smith, J.A.C., and Harberd, N.P. (2013). ROS-mediated vascular homeostatic control of root-to-shoot soil Na delivery in Arabidopsis EMBO Journal **32,** 914-914. http://dx.doi.org/10.1038/emboj.2013.43

Kwak, J.M., Mori, I.C., Pei, Z.M., Leonhardt, N., Torres, M.A., Dangl, J.L., Bloom, R.E., Bodde, S., Jones,

J.D.G., and Schroeder, J.I. (2003). NADPH oxidase AtrbohD and AtrbohF genes function in ROS-dependent ABA signaling in Arabidopsis. EMBO Journal **22,** 2623-2633. http://dx.doi.org/10.1093/emboj/cdg277

Li, N., Sun, L.R., Zhang, L.Y., Song, Y.L., Hu, P.P., Li, C., and Hao, F.S. (2015a). AtrbohD and AtrbohF negatively regulate lateral root development by changing the localized accumulation of superoxide in primary roots of Arabidopsis. Planta **241,** 591-602. http://dx.doi.org/10.1007/s00425-014-2204-1

Li, X.H., Zhang, H.J., Tian, L.M., Huang, L., Liu, S.X., Li, D.Y., and Song, F.M. (2015b). Tomato SlRbohB, a member of the NADPH oxidase family, is required for disease resistance against Botrytis cinerea and tolerance to drought stress. Frontiers in Plant Science **6,** 463. http://dx.doi.org/10.3389/fpls.2015.00463

Ma, L.Y., Zhang, H., Sun, L.R., Jiao, Y.H., Zhang, G.Z., Miao, C., and Hao, F.S. (2012). NADPH oxidase AtrbohD and AtrbohF function in ROS-dependent regulation of Na^+/K^+ homeostasis in Arabidopsis under salt stress. Journal of Exprerimental Botany **63,** 305-317. http://dx.doi.org/10.1093/jxb/err280

Ma, Q., Hu, Z., Mao, Z., Mei, Y., Feng, S., and Shi, K. (2022). The novel leucine-rich repeat receptor-like kinase MRK1 regulates resistance to multiple stresses in tomato. Horticulture Research **9,** uhab088. http://dx.doi.org/10.1093/hr/uhab088

Marro, N., Lidoy, J., Chico, M.Á., Rial, C., García, J., Varela, R.M., Macías, F.A., Pozo, M.J., Janoušková, M., and López-Ráez, J.A. (2021). Strigolactones: New players in the nitrogen–phosphorus signalling interplay. Plant, Cell & Environment **45,** 512-527. http://dx.doi.org/10.1111/pce.14212

Mhamdi, A., Hager, J., Chaouch, S., Queval, G., Han, Y., Taconnat, L., Saindrenan, P., Gouia, H., Issakidis-Bourguet, E., Renou, J.P., and Noctor, G. (2010). Arabidopsis GLUTATHIONE REDUCTASE1 Plays a Crucial Role in Leaf Responses to Intracellular Hydrogen Peroxide and in Ensuring Appropriate Gene Expression through Both Salicylic Acid and Jasmonic Acid Signaling Pathways. Plant Physiol **153,** 1144-1160. http://dx.doi.org/10.1104/pp.110.153767

Mittler, R., Zandalinas, S.I., Fichman, Y., and Van Breusegem, F. (2022). Reactive oxygen species signalling in plant stress responses. Nature Reviews Molecular Cell Biology **23,** 663-679. http://dx.doi.org/10.1038/s41580-022-00499-2

Potikha, T.S., Collins, C.C., Johnson, D.I., Delmer, D.P., and Levine, A. (1999). The involvement of hydrogen peroxide in the differentiation of secondary walls in cotton fibers. Plant Physiol **119,** 849-858. http://dx.doi.org/10.1104/pp.119.3.849

Rodriguez, M., Parola, R., Andreola, S., Pereyra, C., and Martínez-Noël, G. (2019). TOR and SnRK1 signaling pathways in plant response to abiotic stresses: Do they always act according to the "yin-yang" model? Plant Science **288,** 110220. http://dx.doi.org/10.1016/j.plantsci.2019.110220

Sagi, M., and Fluhr, R. (2006). Production of reactive oxygen species by plant NADPH oxidases. Plant Physiol **141,** 336-340. http://dx.doi.org/10.1104/pp.106.078089

Su, D.Y., and Devarenne, T.P. (2018). In vitro activity characterization of the tomato SnRK1 complex proteins. Biochemica ET Biophysica Acta-Proteins and Proteomics **1866,** 857-864. http://dx.doi.org/10.1016/j.bbapap.2018.05.010

Wang, J., Wang, A., Luo, Q., Hu, Z., Ma, Q., Li, Y., Lin, T., Liang, X., Yu, J., Foyer, C.H., and Shi, K. (2022). Glucose sensing by regulator of G protein signaling 1 (RGS1) plays a crucial role in coordinating defense in response to environmental variation in tomato. New Phytologist **236,** 561-575. http://dx.doi.org/10.1111/nph.18356

Wang, Y., Wang, L.P., Micallef, B.J., Tetlow, I.J., Mullen, R.T., Feil, R., Lunn, J.E., and Emes, M.J. (2020). AKINβ1, a subunit of SnRK1, regulates organic acid metabolism and acts as a global modulator of genes involved in carbon, lipid, and nitrogen metabolism. Journal of Exprerimental Botany **71,** 1010-1028.

http://dx.doi.org/10.1093/jxb/erz460

Waszczak, C., Akter, S., Jacques, S., Huang, J.J., Messens, J., and Van Breusegem, F. (2015). Oxidative post-translational modifications of cysteine residues in plant signal transduction. Journal of Exprerimental Botany **66,** 2923-2934. http://dx.doi.org/10.1093/jxb/erv084

Wurzinger, B., Mair, A., Fischer-Schrader, K., Nukarinen, E., Roustan, V., Weckwerth, W., and Teige, M. (2017). Redox state-dependent modulation of plant SnRK1 kinase activity differs from AMPK regulation in animals. FEBS Letters **591,** 3625-3636. http://dx.doi.org/10.1002/1873-3468.12852

Yan, M.Y., Xie, D.L., Cao, J.J., Xia, X.J., Shi, K., Zhou, Y.H., Zhou, J., Foyer, C.H., and Yu, J.Q. (2020). Brassinosteroid-mediated reactive oxygen species are essential for tapetum degradation and pollen fertility in tomato. The Plant Journal **102,** 931-947. http://dx.doi.org/10.1111/tpj.14672

Zhang, L., Wang, L., Fang, Y.C., Gao, Y.H., Yang, S.L., Su, J., Ni, J.B., Teng, Y.W., and Bai, S.L. (2024). Phosphorylated transcription factor PuHB40 mediates ROS-dependent anthocyanin biosynthesis in pear exposed to high light. Plant Cell **00,** 1-22. http://dx.doi.org/10.1093/plcell/koae167

Zhang, Z.H., Li, Z., Wang, W., Jiang, Z.M., Guo, L.P., Wang, X.H., Qian, Y.W., Huang, X.H., Liu, Y.Q., Liu, X.J., Qiu, Y.H., Li, A.F., Yan, Y., Xie, J.P., Cao, S.Y., Kopriva, S., Li, L.G., Kong, F.J., Liu, B.H., Wang, Y.C., Hu, B., and Chu, C.C. (2021). Modulation of nitrate-induced phosphate response by the MYB transcription factor RLI1/HINGE1 in the nucleus. Molecular Plant **14,** 517-529. http://dx.doi.org/10.1016/j.molp.2020.12.005

Zhou, H., Huang, J.J., Willems, P., Van Breusegem, F., and Xie, Y.J. (2023). Cysteine thiol-based post-translational modification: What do we know about transcription factors? Trends in Plant Science **28,** 415-428. http://dx.doi.org/10.1016/j.tplants.2022.11.007

Zhou, J., Xia, X.-J., Zhou, Y.-H., Shi, K., Chen, Z., and Yu, J.-Q. (2014a). RBOH1-dependent H_2O_2 production and subsequent activation of MPK1/2 play an important role in acclimation-induced cross-tolerance in tomato. Journal of Exprerimental Botany **65,** 595-607. http://dx.doi.org/10.1093/jxb/ert404

Zhou, J., Wang, J., Li, X., Xia, X.-J., Zhou, Y.-H., Shi, K., Chen, Z., and Yu, J.-Q. (2014b). H_2O_2 mediates the crosstalk of brassinosteroid and abscisic acid in tomato responses to heat and oxidative stresses. Journal of Exprerimental Botany **65,** 4371-4383. http://dx.doi.org/10.1093/jxb/eru217

Prof. Dr. Jie Zhou
Zhejiang University
Horticulture
Hangzhou, Zhejiang
China

Dear Dr. Zhou:

We have received reviews of your manuscript entitled "SnRK1α1-RBOH1 regulates ROS production and the oxidation of TGA4 to enhance low nitrogen stress tolerance in tomato." Thank you for submitting your best work to The Plant Cell. The editorial board agrees that the work you describe is substantive, falls within the scope of the journal, and may become acceptable for publication, pending revision and potential re-review.

We ask you to pay attention to the following points in preparing your revision:

As you can see from the comments, the referees appreciate the study, however, there are some considerations that should be taken into account before the article is published in the journal. First, the BiFC experiment lacks of appropriate controls (expressing unfused YFP fragments is not a sufficient control for BiFC experiments). In this regard, you can find information about best practices for BiFC experimentation in the journal's Author Guidelines, which also points to this article:
https://academic.oup.com/plcell/article/28/5/1002/6098458
In addition, the reviewers note that the manuscript needs improvements in experimental details and language editing. In this way, we strongly encourage the use of a professional editing service to ensure that the work is presented in the best manner and reaches the broadest possible audience. Several editing services have agreed to extend a discount to authors coming to their website via this ASPB page https://aspb.org/aspb-journal-submission-editing-services/.

Please contact us if there are ambiguous comments or if you wish to discuss the revision.

Given the nature of the comments, we are offering you 60 days from when we have issued this decision to complete the revision. If a revision is not returned within this time frame, and if you have not been granted an extension, we will withdraw the manuscript, which will leave you free to submit the work elsewhere. If you need an extension, we encourage you to contact us at any point before the 60 days have passed.

When you are ready to submit the revised version, please upload a highlighted copy that indicates all changes made in response to the editor and reviewer recommendations. Include an itemized list of all changes made in response to each of the reviewer's suggestions in the "Response to Reviewers" section; please note that reviewers do not have access to your cover letter, nor was this decision letter shared with them.

Thank you for the privilege of reviewing your work. We look forward to receiving your revised manuscript.

On behalf of the editorial board,

Emilio Gutierrez-Beltran, Board of Reviewing Editors
Siobhán Brady, Senior Editor
Blake Meyers, Editor-in-Chief

The Plant Cell

Please note the following:

-The Plant Cell now requires authors to complete and submit an author revisions checklist upon submission of a revised manuscript. The aim of the checklist is to aid authors in preparing a high-quality manuscript, facilitate the review and assessment of revised manuscripts, and help to ensure that journal standards are maintained across the board. If your manuscript is accepted, the completed checklist will be published as supplemental material attached to the article online. Please download a copy of the checklist (pdf fillable form) at this link, for submission with your revised manuscript: https://tpc.msubmit.net/html/Author_Revisions_Checklist.pdf.

-Supplemental materials should be restricted to large datasets and tables, presentation of replicates, and validation of reagents, methods, or genotypes. Any data that are used to support the major claims must be in the main manuscript. Supplemental figure legends must indicate what figure in the main manuscript is supported by the supplemental data presented. Please justify how each of the supplemental figures meet the criteria.

-Sampling methods and nature of "biological replicates" should be described precisely (i.e. different plants, parts of plants, pooled tissue, independent pools of tissue, sampled at different times, etc), along with a clear description of and rationale for any statistical analyses conducted. The reader should know exactly what was sampled; what forms the basis of the calculation of any means and statistical parameters reported. This is also necessary to ensure that proper statistical analysis was conducted.

-Want to add this revision deadline to your calendar? Click below!

--- Reviewer comments:

Reviewer #1 (Comments for the Author):

The study by Zhen et al. identifies a SnRK1-dependent mechanism connecting ROS and plant growth under low nitrogen. In the revised manuscript, the authors addressed my comments regarding experiments. Further, they also provided new connections (TGA4) in the revised manuscript. Overall, I am convinced about the experimental part. However, the manuscript needs improvements in language and experimental details in the text.
My comments are given below,
Major comments
1. Why in pRBOH1::GFP-EV, the GFP signal is on the plasma membrane (Supplementary Fig. 15A)? As this is just a GFP protein, it is generally seen everywhere in the cell.
2. No details on the SnRK1α1 endogenous antibody are provided. Is it a commercial antibody? It is important to show control experiments validating the antibody. In general, details on the antibodies used are not provided (for example AMPK T172, Actin). Authors need to include more information such as RRID and the manufacturer. More details on the protein extraction method and buffer used should be described in the methods. Further, it is important to quantify the blots (Figure 1B, C). Why is Supplementary Fig. 3 mentioned in the methods describing SnRK1 activity assays?
Minor comments
1. No details on SnRK1 activation in the abstract
2. There are no details on the phylogenetic analysis in figure legends or methods
3. #207, to investigate
4. #238, significantly reduced

Reviewer #3 (Comments for the Author):

Dear authors,
I would like to thank the work and efforts performed to improve the ms following our comments and suggestions. My requirements have been accomplished and I think that the manuscript can be accepted for publication in TPC, after minor revisions related to typos and English language.

Comments
English language must be revised by a native speaker. Several corrections (e.g.) can be still found, starting from the abstract.
Examples: L22: ..... Expressing the a-catalytic ...
L35: dependent
L248: exhibited

NRT1.1 should be correctly named in the first citation (NPF6.3/CHL1).

Text should be also revised for typos. E.g. L99: ..responses (Gilroy et al... L312: snrk1 1 mutant
L282: (P<0.05). Please check the entire manuscript, including figure legends.

Editorial requests:

1. As you can see from the comments, the referees appreciate the study, however, there are some considerations that should be taken into account before the article is published in the journal. First, the BiFC experiment lacks of appropriate controls (expressing unfused YFP fragments is not a sufficient control for BiFC experiments). In this regard, you can find information about best practices for BiFC experimentation in the journal's Author Guidelines, which also points to this article: https://academic.oup.com/plcell/article/28/5/1002/6098458

Response: Thank you very much for your advice. We have conducted additional appropriate controls to support the key conclusion of the BiFC experiment. According to the article, we have expressed fused RBOH1/C-nYFP, RBOHA-nYFP (RBOHA is closely related to RBOH1), SnRK1α2-cYFP (SnRK1α2 is closely related to SnRK1α1) as sufficient controls for BiFC experiments, which are shown in Fig. 5E of the revised manuscript (Lines 336-343, lines 751-752). The BiFC signal determined that RBOH1/N-nYFP and cYFP-SnRK1α1 interact based on their colocalized at the plasma membrane (Fig. 5E). To test for specificity, BiFC interactions were also assayed between RBOH1/C-nYFP and cYFP-SnRK1α1, RBOHA-nYFP and cYFP-SnRK1α1, RBOH1-nYFP and cYFP-SnRK1α2, as well as RBOH1/N-nYFP and cYFP-SnRK1α2, where RBOHA is closely related to RBOH1 and SnRK1α2 is closely related to SnRK1α1 (Fig. 5E); none of these lines were shown to interact on the plasma membrane (Fig. 5E). And the results were also verified by yeast two-hybrid (Y2H) assays (Supplementary Fig. 13).

2. In addition, the reviewers note that the manuscript needs improvements in experimental details and language editing. In this way, we strongly encourage the use of a professional editing service to ensure that the work is presented in the best manner and reaches the broadest possible audience. Several editing services have agreed to extend a discount to authors coming to their website via this ASPB page https://aspb.org/aspb-journal-submission-editing-services/.

Response: We have addressed the reviewers' comments by providing more detailed

experimental explanations. We have invited Dr. Hannah Rae Thomas to help us revise the language of the manuscript. Please find below our specific responses to their comments.

Reviewer #1 (Comments for the Author)

The study by Zhen et al. identifies a SnRK1-dependent mechanism connecting ROS and plant growth under low nitrogen. In the revised manuscript, the authors addressed my comments regarding experiments. Further, they also provided new connections (TGA4) in the revised manuscript. Overall, I am convinced about the experimental part. However, the manuscript needs improvements in language and experimental details in the text.

Response: Thank you very much for your positive comments and constructive suggestions. We have incorporated the experimental details and made language improvements as recommended. Below, we provide point-by-point responses to each of your comments.

My comments are given below,

Major comments

1. Why in pRBOH1::GFP-EV, the GFP signal is on the plasma membrane (Supplementary Fig. 15A)? As this is just a GFP protein, it is generally seen everywhere in the cell.

Response: Thank you very much for your reminder and valuable suggestions. Due to the thick, cylindrical structure of the tomato root system, cellular activity varies across different layers. When scanning root cells using laser confocal microscopy, the GFP fluorescence signals also differ between layers. In the previous version of the manuscript, we displayed cells with strong GFP signals on the plasma membrane, to maintain consistency with the root cell scans from other strains. Based on your feedback and to avoid any ambiguity, we have now selected cells where GFP signals are clearly expressed throughout all regions of the cell. These updated images have been included in Supplementary Fig. 16A of the revised manuscript.

2. No details on the SnRK1α1 endogenous antibody are provided. Is it a commercial antibody? It is important to show control experiments validating the antibody. In general, details on the antibodies used are not provided (for example AMPK T172, Actin). Authors need to include more information such as RRID and the manufacturer. More details on the protein extraction method and buffer used should be described in the methods. Further, it is important to quantify the blots (Figure 1B, C). Why is Supplementary Fig. 3 mentioned in the methods describing SnRK1 activity assays?

Response: We have provided detailed information on the SnRK1α1 endogenous antibody, AMPK T172 antibody, Actin antibody and other antibodies in the revised manuscript (Lines 627-634). And we have provided more details on the protein extraction method and buffer used in the revised manuscript, following your suggestion (Lines 620-626). For protein extraction, the root samples were ground in liquid nitrogen and homogenized using an extraction buffer composed of 20 mM HEPES (pH 7.5), 40 mM KCl, 1 mM EDTA, 1% Triton X-100, 10% glycerol, 1 mM phenylmethylsulfonyl fluoride, 5 mM DTT, and 25 mM sodium fluoride. For western blotting, proteins were separated by 10% SDS-PAGE gel and then transferred to nitrocellulose membranes. AMPK T172 protein was detected with anti-AMPK T172 antibody (Cell Signaling Technology, 2535T). SnRK1α1 protein was detected with anti- SnRK1α1 polyclonal antibody (Biospring, BSR231211). ACC pS79 protein was detected with anti- ACC pS79 antibody (Cell Signaling Technology, 3661S). The GFP-tagged ACC1 and the GFP-tagged TGA4 proteins were detected with anti-GFP monoclonal antibody (Thermo Fisher Scientific, MA5-15256). The HA-tagged SnRK1α1 protein was detected with anti-HA monoclonal antibody (Thermo Fisher Scientific, 26183). Actin protein was detected with anti-Actin polyclonal antibody (Abcam, ab197345). Additionally, we have quantified the blots as per your advice in the revised manuscript (Figure 1B, C). We made a mistake of describing Supplementary Fig. 2B as Supplementary Fig. 3, so we have revised it in the revised manuscript (Lines 705-706).

Minor comments

1. No details on SnRK1 activation in the abstract

Response: We have provided details on SnRK1 activation in the abstract (Lines 22-23).

> "Herein, we discovered that the expression and activity of the α-catalytic subunit of SnRK1 (SnRK1α1) increased in response to low nitrogen stress."

2. There are no details on the phylogenetic analysis in figure legends or methods

Response: We have provided detailed information on the phylogenetic analysis in Supplementary Fig. 1 legends, following your suggestion. Homologues of SnRK1 proteins were identified using the Sol genomics network (http://solgenomics.net/) for tomato and TAIR (www.arabidopsis.org) for Arabidopsis. A phylogenetic tree was constructed based on their amino acid sequences using the maximum-likelihood method in MEGA5.0 software.

3. #207, to investigate

Response: We have revised it in the revised manuscript (Line 201).

4. #238, significantly reduced

Response: We have revised it in the revised manuscript (Line 229).

Reviewer #3 (Comments for the Author): Dear authors, I would like to thank the work and efforts performed to improve the ms following our comments and suggestions. My requirements have been accomplished and I think that the manuscript can be accepted for publication in TPC, after minor revisions related to typos and English language.

Response: Thank you very much for your help in revising and improving this manuscript and your recognition of our research. We have made minor revisions related to typos and English language as suggested. Our responses to your comments are listed below, point-by-point.

Comments

1. English language must be revised by a native speaker. Several corrections (e.g.) can be still found, starting from the abstract.

Examples: L22: ..... Expressing the a-catalytic ...

L35: dependent

L248: exhibited

Response: We have invited Dr. Hannah Rae Thomas. to help us revise the language of the manuscript. We have also made revisions for your suggestions in the revised manuscript (Line 22, line 35, line 236).

2. NRT1.1 should be correctly named in the first citation (NPF6.3/CHL1).

Response: We have revised it in the revised manuscript (Line 54).

3. Text should be also revised for typos. E.g. L99: ..responses (Gilroy et al... L312: snrk1α1 mutant. L282: (P<0.05). Please check the entire manuscript, including figure legends.

Response: Thank you for your advice, we have revised the typos in the revised manuscript (Lines 95-96, line 293, line 264) and checked the entire manuscript, including figure legends.

Dear Dr. Jie Zhou:

We are pleased to inform you that your paper entitled "SnRK1α1-RBOH1 regulates ROS production and the oxidation of TGA4 to enhance low nitrogen stress tolerance in tomato" has been accepted for publication in The Plant Cell, pending a final editorial review by a science editor. At this stage, your manuscript will be evaluated by a science editor with respect to its presentation of scientific content, compliance with journal policies, and presentation for a broad readership. The Plant Cell has contracted with Plant Editors (planteditors.com) to provide this service to our authors, and you will soon receive additional information on this process.

Please note that each author needs to link their ORCID identifier to their account in the system before your manuscript can be published. If any authors do not have an ORCID linked to their account, they will receive a message with a link to complete this task. Please ensure that ALL of your coauthors have completed this task as soon as possible.

LICENSE INFORMATION Shortly after your paper is sent to production, you will receive an email from our publisher's author support team, **SciPris**, with information on article processing charges, as well as the types of Open Access license you would be able to purchase. The accepted version of your manuscript will not appear on our Advance Articles page until the license is selected and signed, so please look out for an email from SciPris. For more information see the Article Publication Fees section of our author guidelines: https://academic.oup.com/plcell/pages/General-Instructions

The Plant Cell and The Arabidopsis Information Resource (TAIR) are collaborating to collect functional annotation data about Arabidopsis genes from authors. This includes information about the gene's molecular function (e.g., kinase activity, ATP synthetase activity), the biological process/es it is involved in (e.g., endosperm development, threonine biosynthesis), its subcellular location (e.g., nucleus, ER), anatomical or developmental expression pattern (e.g., leaf, ovule, flower stage 10, seedling stage), or its partner in a protein-protein interaction (e.g., AT1G01010 interacts with AT1G01020).

If your paper contains results falling into one or more of these categories for Arabidopsis genes, we request that you now submit these data for inclusion in TAIR by filling in the form provided at the following URL: https://www.arabidopsis.org/doc/submit/functional_annotation/123. If you need further clarification on what types of data can be submitted please contact curator@arabidopsis.org.

Finally, we encourage your submission of artwork for the journal cover. If there is an image or illustration related to your paper that you would like to have considered, please email it in .pdf or .tif format to clowe@aspb.org. An image title, brief, 2-3 sentence description of the image, and the name of the person credited for the image are also encouraged. If your image is selected to appear on the online cover of the journal, you will be contacted with the scheduled issue date and further information.

We look forward to seeing your paper published.

On behalf of the editorial board,

Emilio Gutierrez-Beltran, Board of Reviewing Editors
Siobhán Brady, Senior Editor
Blake Meyers, Editor-in-Chief

The Plant Cell

=====
IMPORTANT REMINDER: PEER REVIEW REPORTS
=====

If you opted to publish a peer review report along with your article during the original submission process, it will be prepared by the editorial staff and publicly posted with your manuscript, inside the zip file that contains any other supplemental material. As a reminder, the peer review report is a public record of all comments from editors and reviewers, as well as your prior responses, as you received them in the decision letters for each draft of your manuscript. If you agreed to publish this report and have changed your mind, or are not sure if you selected this option, please contact the editorial office as soon as possible before signing the license agreement from our publisher.

=====

----FOR ASPB OFFICE USE ONLY (DO NOT EDIT)----

MSID: 41213
Corresponding Author: Prof. Dr. Zhou
